# Condensate corona–nanoparticle complexes transfer functional biomolecules between cells

**DOI:** 10.1038/s41563-026-02534-5

**Published:** 2026-04-16

**Authors:** Laurent Adumeau, Yuchen Lin, Mura M. McCafferty, Silvia Vercellino, Yi-Feng Wang, Xiaoliang Yang, Wei Zhang, David Garry, Filippo Bertoli, Cara Gaffney, Ying Ling Dee, Linlin Song, Ester Canepa, Xia Xiao, Yanqiu Ye, Guohui Huang, Qiwei Wang, Liufang Liao, Zixu Zhao, Koen Evers, Lorenzo Cursi, Vanya Petseva, Zengchun Xie, Aisling Fleming, Emily Sheridan, Ingrid Morera, Kai Liu, Yingxin Li, Marta Saccomanno, Andrea Marcantognini, Yan Yan, Kenneth A. Dawson

**Affiliations:** 1 Centre for BioNano Interactions, School of Chemistry, University College Dublin, Dublin, Ireland; 2 School of Biomolecular and Biomedical Science, UCD Conway Institute of Biomolecular and Biomedical Research, University College Dublin, Dublin, Ireland

**Keywords:** Nanoscale materials, Nanomedicine, Nanobiotechnology

## Abstract

Biological nanoscale assemblies transfer proteins and RNAs between cells and cellular compartments. Nonetheless, it is unclear if exogenous and synthetic nanostructures affect these molecular assemblies and processes. Here we report nanostructure–biological hybrid complexes that are formed by synthetic nanoparticles after being internalized by cells. These nanoparticles, in rare events, acquire an overlaid cell-derived biomolecular condensate corona, afterwards being exported to the extracellular space to be internalized by other cells. The condensate corona is compositionally distinct from extracellular vesicles, containing intact proteins, mRNAs and long RNAs. The condensate corona is mechanically robust in extracellular conditions, becoming fluid within endosomes, where it detaches from the particle core and escapes the endo-lysosomal pathway, redistributing its protein and RNA components to cytosolic and nuclear compartments. Grafting short peptides onto the surface of purified corona–nanoparticle complexes prevents detachment and endo-lysosomal escape, suggesting that recognition interactions at the condensate–endosome lumen interface control intracellular access. Overall, these findings reveal a natural, condensate-mediated route for the transfer of biomolecular machinery including RNA between cells, which could inspire design principles for future delivery systems.

## Abstract (web summary)

After being internalized by cells, nanoparticles can, in rare events, acquire a biomolecular condensate corona. These complexes can reach the extracellular space, where they can deliver their acquired proteins and RNA content to other cells.

## Main

In recent years, many nanoscale mechanisms for biomolecular transfer and cell-to-cell communication have been described^1–4^. These include tunnelling nanotubes, nanoscale cell–cell contact interfaces, and multiple classes of cell-derived, actively exported nanostructures such as extracellular vesicles (EVs) and non-vesicular particles^5–7^. Improved isolation procedures now allow better-defined populations to be recovered^8^, and it is argued that both vesicular and non-vesicular nanostructures could contribute to communication and regulation at distal sites^9,10^. However, the specific interactions and mechanisms underlying such nanostructure-mediated communication remain difficult to clarify^11–13^, in part because the biological regulations of greatest interest involve comparatively rare events occurring within highly heterogeneous nanostructure populations.

Our investigations of nanoparticle–cell interactions have focused on how biological recognition across some tens of nanometres shapes cellular responses^14,15^. The composition and organization of the surface coating (‘biomolecular corona’), together with the particle shape, determine how cells recognize nanostructures and, therefore, their uptake routes and downstream fates^16–20^. Our working hypothesis is that such nanoscale recognition events control critical decisions at apposed surfaces of tens of nanometres, whether between membranes, at corona–membrane interfaces or within cargo-carrying endosomes^4^. In the absence of favourable recognition events, nanostructure-bearing endosomes are expected to follow a default endo-lysosomal pathway towards degradation, protecting the cytosol and nucleus from harmful exposure. As with extracellular nanostructures, our studies led us to conclude that, embedded within complex background populations, a small subset of original corona-coated particles is processed differently, traversing unconventional non-endo-lysosomal pathways to be re-exported in a distinct form that allows privileged biological access^21^. We reasoned that if it were possible to isolate the new nanostructures that participate in these rare functional events, their architectures would encode design principles for biologically permissive access and to inform the engineering of delivery systems.

This paper focuses on the production, isolation, composition and architecture of these ‘rare-event’ nanostructures (‘particle complexes’), and shows that they enable cellular transfer of intact and functional biomolecules.

## Rare exported particles bear overlaid cell-derived coronas

**Fig. 1:**
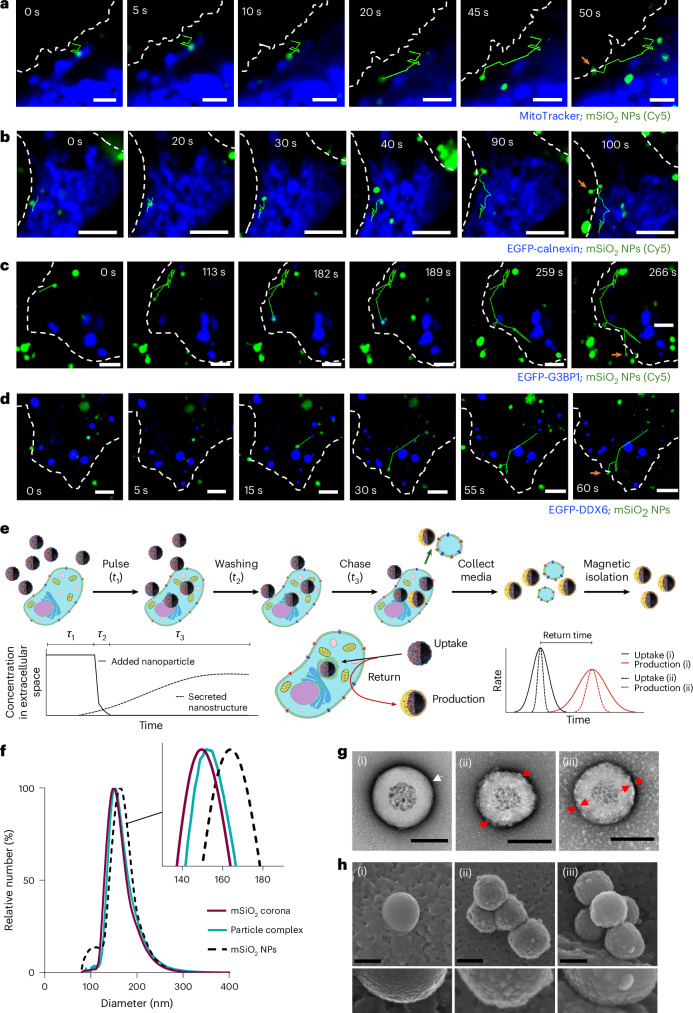
Biogenesis, isolation and characterization of extracellular particle complexes. **a**–**d**, Particle trajectories of mSiO_2_-serum biomolecular corona-coated nanoparticles (NPs) that are re-exported as extracellular particle complexes from HEK293 cells, captured using live-cell confocal microscopy. These particles undergo extended intracellular residence with repeated contacts at the mitochondria (**a**; MitoTracker), ER (**b**; EGFP-calnexin), stress granules (**c**; EGFP-G3BP1) and P bodies (**d**; EGFP-DDX6) before release at the plasma membrane (white dashed line; arrows). Green mSiO_2_ cores (Cy5). Scale bars, 5 μm. Similar exporting trajectories for each marker were observed in numerous (>3) independent experiments. **e**, Schematic of the pulse–wash–chase workflow used to produce particle complexes and limit contamination due to unprocessed particles and other extracellular nanostructures. Typical example, *t*_1_ = 10 min, rapid washing *t*_2_ = 3 × 1–5 min and complexes collection *t*_3_ = 60–240 min using magnetic isolation. *t*_1_–*t*_3_, successive pulse, wash and chase phases; *τ*_1_–*τ*_3_, corresponding durations of these phases. **f**, DCS of bare mSiO_2_ (i), serum-corona mSiO_2_ (ii) and A549-derived particle complexes (iii) after a 10-min pulse and 4-h chase, showing the additional cell-derived layer on complexes. **g**, Representative transmission electron microscopy micrographs of bare mSiO_2_ (i), serum-corona mSiO_2_ (ii) and A549-derived (iii) particle complexes. White arrow, uranyl acetate accumulation at the bare mSiO_2_ surface; red arrows, serum corona (ii) and overlaid cell-derived coating on complexes (iii). Scale bars, 100 nm. **h**, Representative scanning electron microscopy images of bare mSiO_2_ (i), serum-corona mSiO_2_ (ii) and complexes on cell surfaces (iii). The smooth mSiO_2_ surface is clearly distinguished; structural differences between serum coronas and overlaid complexes are more subtle. Similar scanning electron microscopy images were obtained in numerous (>3) independent experiments. Scale bars, 100 nm.

We use magnetic-cored, silica-shelled nanoparticles precoated with a grafted or adsorbed biomolecular corona that serves as a scaffold. After uptake, a subpopulation of these scaffolds acts as an intracellular recognition cue, triggering the deposition of a secondary, cell-derived corona and the resulting hybrid particle complex is then re-exported. We explored a range of core–shell particle designs and producer cell types but since the general behaviour was similar (for example, see Supplementary Videos 1–3), we focus here on magnetic-cored, silica-shelled particles bearing conventional serum-derived non-specific coronas in several cell lines. These model particles are well dispersed and carry internal fluorescent labels in the silica shell, allowing a direct visualization of their intracellular trajectories (Fig. 1 and Supplementary Figs. 1–3). Particle complexes are isolated using magnetic racks and cores optimized for rapid, homogeneous extraction^22^^,23^ (Supplementary Figs. 4 and 5), and corona formation or surface grafting follows established methods^24^.

After internalization, most particles accumulate in lysosomes, but a minority follows rare-event trajectories that culminate in particle hybrid complex re-export at later times (Fig. 1 and Supplementary Fig. 6). Live-cell imaging indicates that endosomes with these particles undergo repeated clusters of collision contacts with intracellular targets, including mitochondria (Fig. 1a, MitoTracker), endoplasmic reticulum (ER; Fig. 1b, EGFP-calnexin), stress granules (Fig. 1c, EGFP-G3BP1) and P bodies (Fig. 1d, EGFP-DDX6). Across different particle surfaces and cell types (Supplementary Videos 1–3), re-exported particles consistently retain much of their original nanoparticle–serum corona but acquire this new, overlaid cell-derived layer that can be measured and analysed (Fig. 1e–h). Intracellular residence and release times span a wide range, with some trajectories extending to many tens of minutes, qualitatively distinct from short-term recycling and characterized by repeated intracellular contacts. Because of this broad distribution, it is difficult to capture full, unperturbed trajectories by live-cell imaging and the examples shown in Fig. 1a–d are characteristic but drawn from shorter round-trip events. To obtain population-averaged kinetics, we, therefore, used pulse–chase protocols followed by the magnetic isolation of exported complexes, using core fluorescence as a proxy for release. The resulting release half-lives are on the order of several tens of minutes (Supplementary Fig. 6), and the kinetic curves suggest that beyond a point of no return, the remaining particles are trafficked irreversibly to lysosomes on an approximately 6-h timescale. The release rate constants vary with the system details and producer cell state but are comparable with other extracellular nanostructures^25^.

## Formation and physical characteristics of overlaid corona

Typical extracellular nanostructure isolation yields complex and unresolvable mixtures of unprocessed particles, secreted nanostructures, cellular debris and particle coacervates (Supplementary Fig. 7). Magnetic extraction isolation alone is insufficient to remove all confounding components. However, by combining magnetic core extraction with an optimized pulse–chase regime (exposure *t*_1_, cell wash *t*_2_ and chase-collection *t*_3_) and post-isolation washing (the schematic is shown in Fig. 1e–h), we obtained highly reproducible particle-complex isolates with minimal background contamination^22^. The overlaid cell-derived coating is reflected in an increased effective size of the released complexes (Fig. 1f) and in the compositional changes described throughout the paper. Differential centrifugation sedimentation (DCS) measurements provide precise, reproducible ‘size fingerprints’ (Fig. 1f) that are based on a single-particle density^26^. Thus, for the purpose of this work, the raw DCS peak shifts (rather than absolute sizes) are used to compare original non-specific and cell-derived overlaid corona complexes across experiments.

The overlaid corona is solid-like, structurally stable and biochemically robust. Electron microscopy of bare silica, silica with serum corona and these extracellular particle complexes suggests that the additional cell-derived layer has a more punctate surface morphology than the serum corona (Fig. 1g,h and Supplementary Figs. 8 and 9), although further high-resolution studies are needed to resolve the surface organization. Particle complexes retain their coating under a range of physiologically relevant conditions, including those typical of late endocytic compartments. Treatments aiming to emulate those conditions fail to detach the layer (Supplementary Fig. 10), implying large disjoining energies at the interface between the serum-derived and cell-derived layers. Consequently, relatively harsh conditions similar to those used to extract serum coronas are required to remove the overlaid corona for detailed analyses.

## Particle complexes have an RNA-granule-enriched proteome

**Fig. 2:**
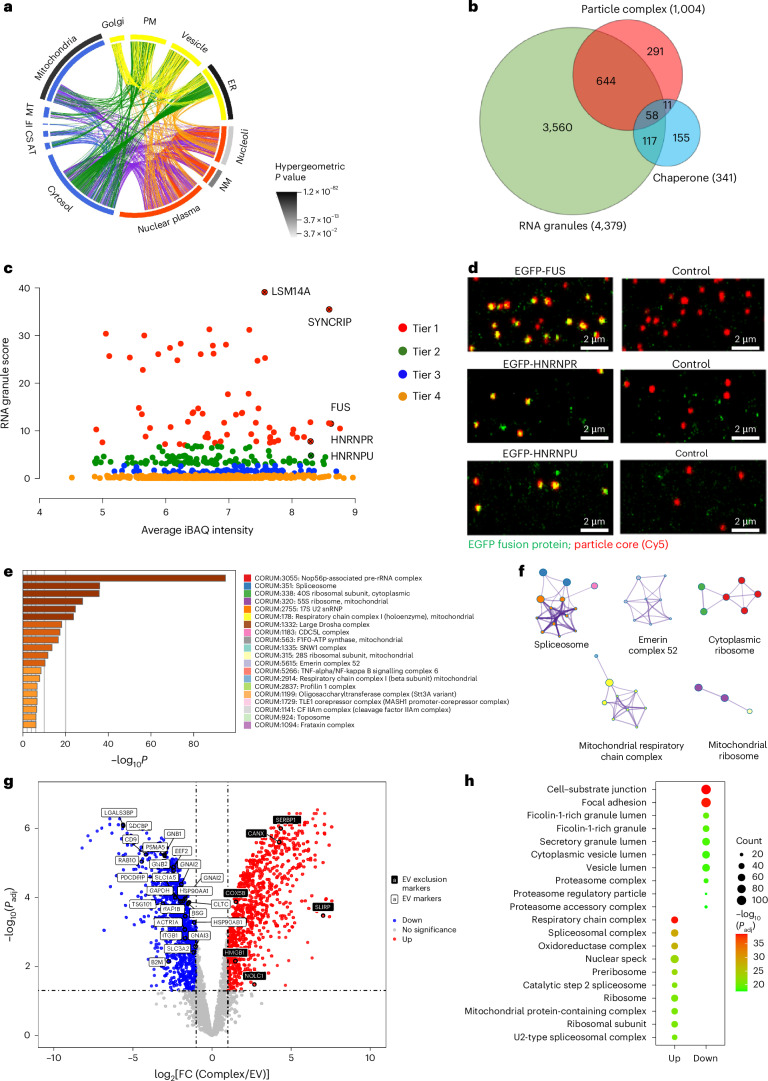
Proteome of cell-derived overlaid corona component of particle complex. **a**, Predicted subcellular locations of proteins identified in A549-derived particle complexes, using Human Protein Atlas annotations and SILAC proteomics (three biological replicates). The inner coloured segments indicate the proportion of the proteome in each compartment; arcs show multilocalized proteins. The outer greyscale segments indicate enrichment significance based on hypergeometric tests. IF, intermediate filaments; AT, actin filaments; MT, microtubules; CS, centrosome; PM, plasma membrane; NM, nuclear membrane. **b**, Venn diagram showing an overlap between the particle-complex proteome (red), RNA granule database^28^ (green) and manually curated chaperone families^29^ (blue). **c**, Granule curation scores^28^ (the confidence with which proteins are considered likely causally linked to RNA granules) for 702 particle-complex proteins annotated as granule proteins (tiers 1–4). Examples of highly abundant granule proteins (for example, LSM14A, SYNCRIP, FUS, HNRNPR and HNRNPU) are labelled. iBAQ, intensity-based absolute quantification. **d**, Particle-by-particle imaging of complexes generated from HEK293 cells expressing EGFP-fusion granule proteins. Red, particle core (Cy5); green, EGFP-fusion. Major granule proteins are present on nearly all complexes. Similar images were obtained in >5 independent experiments. **e**, CORUM enrichment analysis of the A549 particle-complex proteome, ranked by statistical significance. **f**, Network representations of selected enriched CORUM complexes, highlighting interactions between complexes. **g**, Volcano plot showing differentially abundant proteins between HEK293-derived particle complexes and EVs (four biological replicates each). Differential analysis was performed with limma (moderated two-sided *t*-tests; Benjamini–Hochberg adjusted *P* values); points passing false-discovery rate < 0.05 and |log_2_FC | ≥ 1 (FC, fold change) are coloured. EV markers are enriched in EVs, whereas EV exclusion markers are enriched in particle complexes. **h**, Gene Ontology term enrichment (cellular component) of differential proteins between complexes and EVs, showing that complexes are enriched for splicing, translation and mitochondrial complexes, whereas EVs are enriched for extracellular and vesicular lumen (bubble size, count; colour, –log_10_(adjusted *P*)). Source data

Optimized isolation, washing and analysis procedures rendered the protein profiles shown in Fig. 2 highly reproducible across investigators and experiments. Stable-isotope amino acid labelling (SILAC) of the producer cells, the production of labelled nanoparticle–condensate complexes, followed by mass spectrometry allowed us to distinguish cell-derived condensate coronas from residual serum coronas and to analyse the former selectively (Supplementary Fig. 11). Using the Cell Atlas^27^, we found that although the identified proteins can traffic between locations, as a group, they show the strongest associations with ER and mitochondria (outer ring; Fig. 2a), consistent with the intracellular contacts observed during biogenesis. In particular, most of the proteome (around 70%; Fig. 2b) consists of proteins previously associated with mesoscopic intracellular RNA granules^28^ (Fig. 2c), as captured in RNA granule databases and exemplified by P bodies and related structures. Many chaperones and co-chaperones (from curated human chaperone families^29^) were also present. In RNA granules, these have been implicated in reversible condensation melting and in maintaining the protein structure during functional insertion into target organelles^30^. Several well-known RNA granule proteins (for example LSM14A, SYNCRIP, FUS and multiple heterogeneous nuclear ribonucleoproteins) are particularly abundant (Fig. 2c). To assess condensate homogeneity across individual complexes, we prepared particle complexes from cells stably expressing EGFP-tagged RNA granule proteins and imaged them particle by particle. Major proteins are incorporated into nearly all exported complexes rather than being confined to small subpopulations (Fig. 2d), and the corresponding mRNAs are also often detected. The proteome shows highly correlated clusters corresponding to complexes annotated in the CORUM (the comprehensive resource of mammalian protein complexes) database (Fig. 2e,f), many shared across multiple particle and producer cell types, effectively ‘biomarker-complex-like’.

Several identified proteins were validated by western blot (Supplementary Fig. 12). Prominent EV markers such as TSG101 are absent from both proteomics and western blots of particle complexes, prompting a closer comparison of particle complex and EV proteomes. Thus, we compared particle complexes with EVs (standard EV isolation) from the same cell populations (for example, SILAC-adapted HEK293 cells). The two proteomes differ markedly (Fig. 2g,h and Supplementary Fig. 13). As expected, many commonly cited EV markers are strongly enriched in EV samples^31^, but essentially absent in particle complexes, whereas exosome ‘exclusion’ biomarkers^31^ are significantly more abundant in the particle complexes (Fig. 2g). Proteins enriched in particle complexes are strongly associated with mitochondria, spliceosomes, ribosomes and other RNA-granule-like machineries (for example, nuclear speckle and ER protein-containing complex) (Fig. 2h and Supplementary Fig. 14), whereas proteins enriched in EVs are associated with the extracellular matrix and vesicular lumen (Fig. 2h and Supplementary Fig. 14). Comparable differences were observed when nanoparticle complexes were compared with EVs generated under non-starved conditions, suggesting that the marked divergence in proteomes and RNA profiles reflects their fundamental architectures rather than specific EV production conditions (data not shown).

## Particle complexes carry intact granule-linked RNAs

**Fig. 3:**
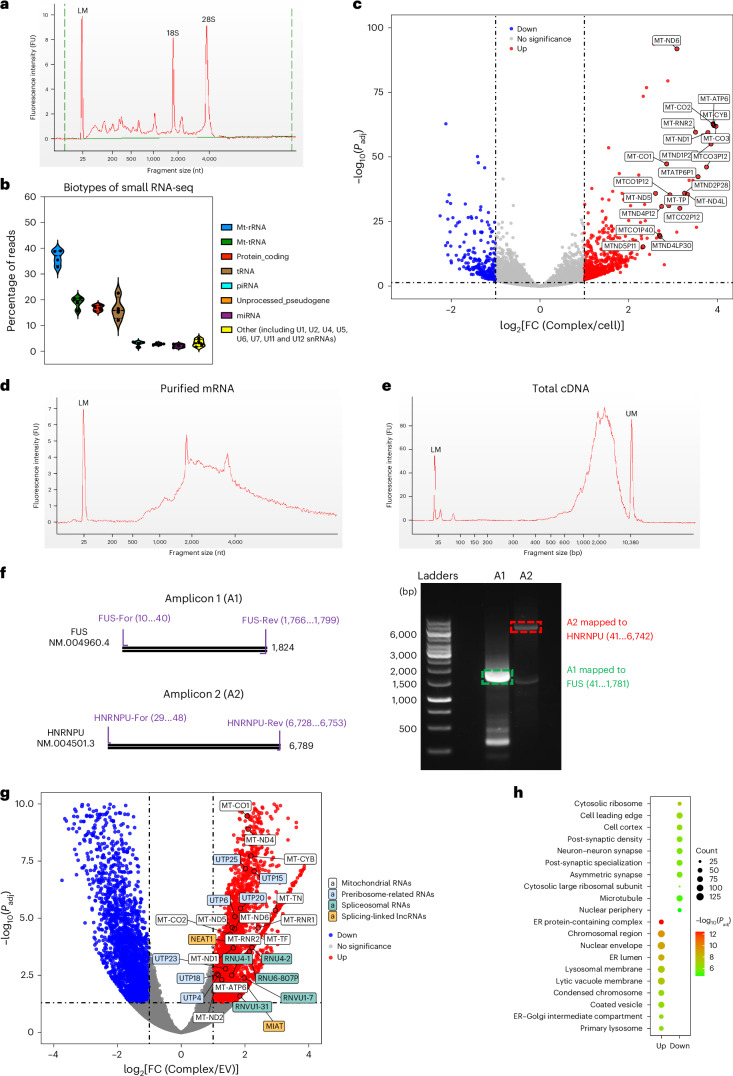
Transcriptome of particle complex. **a**, Total RNA profile of A549-derived particle complexes analysed on an Agilent bioanalyser (RNA 6000 Pico kit), showing small RNA, 18S/28S rRNA and long RNA peaks. LM, ladder marker. Profiles differ from typical EV RNA profiles. FU, fluorescence unit. **b**, Biotypes from small RNA-seq from four biological replicates of A549-derived particle complexes. Violin plots show mitochondrial, spliceosomal RNAs, tRNA, miRNA and other classes; points represent the samples, and the central line indicates the median. **c**, Volcano plot of mRNA-seq comparing particle complexes to parental A549 cells (four biological replicates each). Differential expression was analysed with DESeq2 (two-sided Wald test; Benjamini–Hochberg adjusted *P* values), with significant genes defined as adjusted *P* < 0.05 and log_2_FC(Complex/A549) ≥ 1. The most enriched mitochondrial-associated mRNAs are labelled. **d**, mRNA profile from HEK293-derived particle complexes after poly(A) selection, showing abundant long mRNA species. **e**, cDNA profile of HEK293-derived complexes after full-length mRNA reverse transcription and cDNA amplification, confirming the presence of long transcripts. **f**, Left: schematic of two amplicons (A1 and A2) spanning the coding regions of FUS and HNRNPU. Right: DNA electrophoresis of PCR products generated from reverse-transcribed cDNA using these primer pairs. Gel-purified bands (dotted boxes) were Sanger sequenced and mapped to the expected coding regions; similar results were obtained in two independent experiments. **g**, Volcano plot of RNA-seq comparing HEK293-derived particle complexes and EVs (three and four biological replicates, respectively). Differential expression was analysed with DESeq2 (two-sided Wald test; Benjamini–Hochberg adjusted *P* values); RNAs with adjusted *P* < 0.05 and |log_2_FC(Complex/EVs)| ≥ 1 are coloured. Complex-enriched RNAs (red) include mitochondrial RNAs, spliceosomal RNAs, splicing-linked lncRNAs and preribosome-related RNAs. **h**, Gene Ontology cellular-component enrichment of differential RNAs. Source data

We extracted and profiled RNA from particle complexes (Fig. 3a and Supplementary Figs. 15–21). Bioanalyser profiles of total RNA consistently show strong, small RNA peaks, prominent 18S and 28S rRNAs, and longer RNA components (Fig. 3a and Supplementary Figs. 16 and 17). Because standard RNA workflows are optimized for cellular RNA rather than endogenous nanostructures, we compared several isolation protocols and chip types before sequencing. Although details differed, the overall conclusions were consistent, and we adopted workflows optimized for each sample type (Supplementary Fig. 16). RNA sequencing (RNA-seq) revealed a range of species, including mitochondrial RNAs, tRNAs, miRNAs and mRNAs (Fig. 3b), along with structural RNAs from cytoplasmic and mitochondrial ribosomes and from major and minor spliceosomes, as suggested by the proteomic data. Mitochondrial RNAs are enriched in particle complexes relative to whole-cell RNA (Fig. 3c), consistent with the abundance of mitochondrial proteins in the proteome (Fig. 2). Together with the proteome, the prominence of splicing, translation and mitochondria-related RNAs supports the involvement of ER–nucleus and mitochondrial biogenesis pathways, as discussed earlier (Figs. 1 and 2a).

Given the recent claims that EV mRNAs are predominantly fragmented^32^, we examined particle-complex mRNAs using several approaches, including long-read RNA methods. Poly(A)^+^ RNA was purified from HEK293-derived particle complexes with oligo(dT) beads and analysed using a bioanalyser RNA Pico chip (Fig. 3d). Small RNA chip analysis of samples and supernatants revealed no detectable fragmented mRNA (Supplementary Fig. 18). Bioanalyser profiles identify a pool of long mRNAs (Fig. 3d), with the most abundant transcripts between 2,000 and 4,000 nt and substantial populations at still longer lengths. We next performed reverse transcription and cDNA amplification, and observed cDNAs spanning the expected size ranges (Supplementary Fig. 19). Bioanalyser profiles of the total mRNA and cDNA (Fig. 3d,e) are, therefore, consistent in identifying long mRNAs. Long-read RNA-seq similarly revealed long, apparently full-length transcripts. As Sanger sequencing is often used to verify long-read calls, we provide illustrative examples (Fig. 3f): primers spanning the coding regions of FUS or HNRNPU were used to amplify cDNA, amplicons were gel purified and sequenced, and the products corresponded to the expected coding regions. Comparable results across cell types and particle core scaffolds suggest that particle complexes typically carry long, apparently intact mRNAs (if present, any broken mRNA is below our current detection limits). This supports particle-borne nanoscale condensates as a paradigm for capture, protection and potentially distal transport of intact mRNA.

To further support the distinctiveness of particle complexes, we compared RNA-seq profiles of particle complexes and EVs from identical cell populations. Volcano plots (Fig. 3g), heat maps and principal component analysis (Supplementary Fig. 20) clearly separate the pools, with 4,082 genes enriched in particle complexes and 2,523 in EVs (Supplementary Fig. 21), spanning mRNAs, long non-coding RNAs (lncRNAs) and small RNAs. In interpreting these data, we emphasize that particle-complex mRNAs have been independently validated by long-read approaches and targeted Sanger sequencing (Fig. 3f), and therefore, they can be treated as intact transcripts. By contrast, EV RNA profiles, although consistent with typical results in the literature, are used here primarily to demonstrate differences. We find that particle complexes are strongly enriched for mitochondrial mRNAs, structural RNAs of spliceosomes (including canonical, variant and pseudo U-family genes), ribosome biogenesis-related genes and splicing-linked lncRNAs (Fig. 3g). Gene Ontology cellular-component analysis of particle-complex-enriched transcripts shows strong association with ER, nuclear envelope/peripheral structures and lysosomal membrane (Fig. 3h). Together with the proteome, the co-enrichment of structural RNAs and structural proteins for spliceosomes, ribosomes and mitochondrial RNA granules suggests that the components of splicing and translation machineries are present within the particle complexes, potentially in partially assembled forms. These probably originate from organelle contact sites among endo-membranes, mitochondria and ER, where translation and partial local splicing machineries are deposited, but single-particle analyses will be required to analyse individual rather than ensembles of events.

## Lipid-poor non-enveloped complexes stabilize long RNAs

Particle complexes show no evidence of a continuous protective lipid enclosure around the condensate layer, though some samples display limited CellMask Orange (CMO) staining (Supplementary Fig. 22). Despite this, biomolecules in particle complexes, including long RNAs, are resilient under typical extracellular conditions and degraded only after prolonged exposure to high concentrations of nucleases (Supplementary Fig. 23), indicating incomplete protection. In situ degradation of long RNAs using high levels of RNase yields fragments of around 20 nt (Supplementary Fig. 23), resembling footprints around RNA-binding proteins^33^ and consistent with RNA granule architectures. To directly probe the role of lipids, we compared lipidomic liquid chromatography–tandem mass spectrometry (LC–MS/MS) profiles of EVs and particle complexes from the same cell populations using equal particle/EV numbers (comparable surface area). EVs are dominated by phosphatidylcholine (40%–60%) and sphingomyelins (20%–30%) (Supplementary Fig. 24), which recapitulates previous reports^34^. By contrast, particle complexes contain very little lipid. Furthermore, phosphatidylcholine and sphingomyelins—characteristic of enveloping membranes—are essentially absent. Only trace amounts of specific lipid classes, some previously linked to RNA granules, are detected—insufficient for a continuous lipid coat. Together with the proteomic and RNA analyses (Figs. 2 and 3), the RNA footprinting and lipidomics assays support the conclusion that particle complexes are non-enveloped, lipid-poor condensates that share key molecular and organizational features with RNA granule condensates.

This observation has two immediate implications. First, after endosomal uptake, particle-borne condensates directly contact the endosomal lumen, establishing a nanoscale recognition and delivery mode partly reminiscent of non-enveloped viruses. Second, the striking extracellular resilience of these non-enveloped condensates suggests an architectural strategy (for example, solid-like ‘glassy’ RNA granule) that preserves mRNAs and other biomolecules intact over extended periods under typical extracellular conditions. However, such dense packing does not obviously support intracellular function. In the next section, we show that uptake into cells triggers a change in state of the condensate layer before further processing.

## Particle complexes enable cellular transfer

**Fig. 4:**
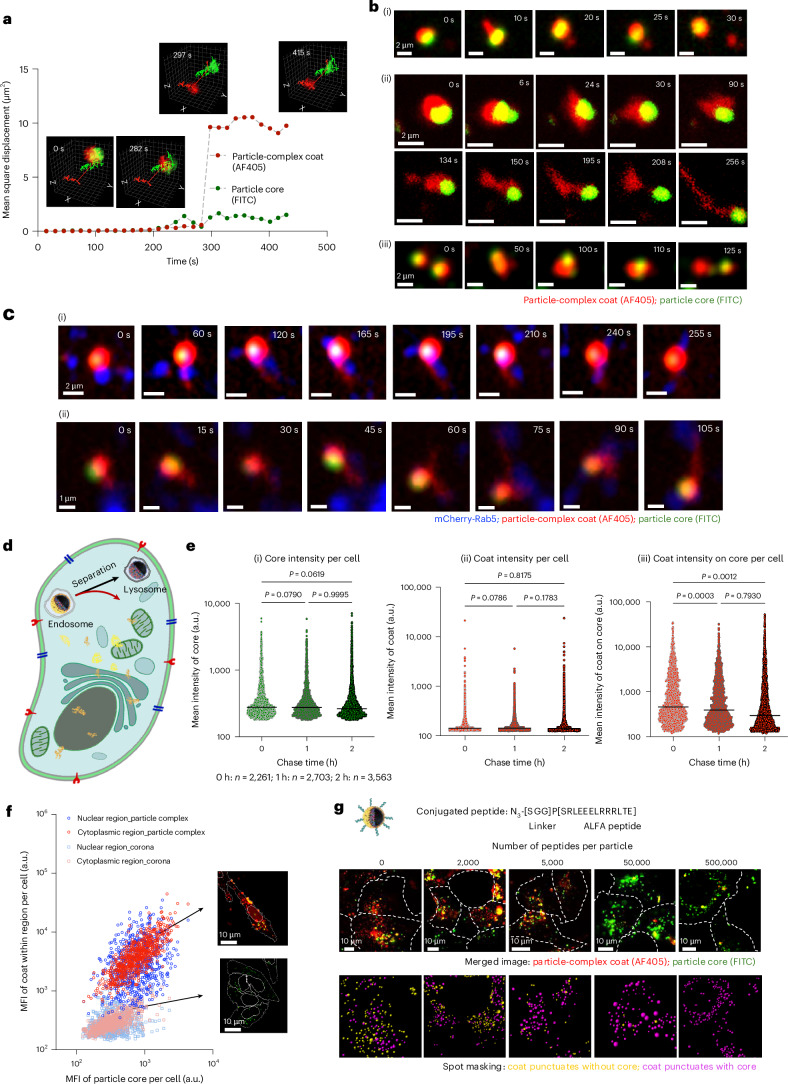
Detachment, separation and escape of particle-complex condensate layer. Cells were pulsed with 40 μg ml^−1^ particle complexes for 2 h. Coat proteins were labelled with NHS fluorophores (AF405 or AF568, red) and cores with FITC (green). **a**, Mean square displacement over time showing the coat detaching from the particle core in A549 cells. Insets: the initial co-movement and subsequent independent movement of the coat and core. **b**, Time-lapse images of coat detachment within HEK293 cells. (i) Part of the coat detaches and separates from the core. (ii) Coat fluidizes first and then flows across the compartment, dispersing locally. (iii) Coats from two separate endosomes are shared and sorted. **c**, Two examples of coat detachment and escape in Rab5-positive compartments (mCherry-Rab5, blue). Each mode of detachment and separation was observed in >5 independent experiments. **d**, Schematic of condensate separation from particle-containing endosomes and the subsequent routing of cores towards lysosomes. **e**, High-content quantification of core and coat fluorescence per cell in HEK293 cells at 0, 1 and 2 h of chase. Each point represents a single cell, and the line indicates median. The evolving violin-plot shapes of the coat on core are consistent with coat rearrangement: a few endosomes gain coat at the expense of others, amid overall coat depletion from endosomes. One-way ANOVA with Tukey’s multiple comparisons test (two-sided). **f**, High-content analysis of A549 cells showing nuclear and cytoplasmic mean fluorescence intensity (MFI) of coat versus core for particle complexes and serum-corona particles after 18 h of pulse and 4 h of chase. Insets: the representative segmented cells from the two populations. **g**, ALFA-peptide grafting on condensate blocks endosomal escape. Top row: merged images of coat (AF405; red) and cores (FITC; green) at increasing ALFA densities per particle; higher grafting causes the condensate to remain with cores and restores lysosomal degradation. Bottom row: masked images distinguishing detached coat puncta without cores (yellow) from the core-associated coat (magenta). The ALFA-functionalized particle complexes were presented to A549 cells for 2 h of pulse and 6 h of chase. For each grafting density, at least ten similar images were obtained. Source data

**Fig. 5:**
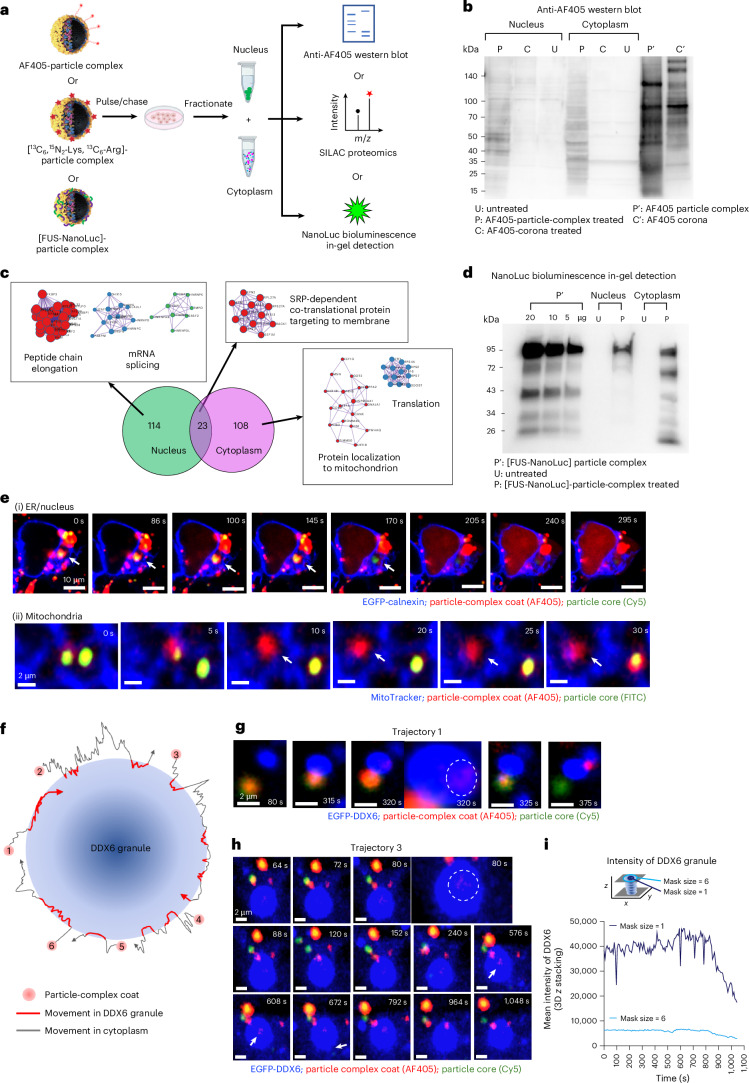
Redistribution of particle-complex condensate in recipient cells. **a**, Workflow to analyse coat redistribution between cytoplasmic and nuclear fractions using three labelling strategies. **b**, Anti-AF405 western blots of nuclear and cytoplasmic fractions from A549 cells treated with AF405-labelled particle complexes or corona (18 h of pulse and 4 h of chase) show intact coat proteins and distinct cytoplasmic versus nuclear banding after particle-complex treatment. Loaded: 60 µg of nuclear lysate, 86 µg of cytoplasmic lysate and lysates from AF405-labelled particle complex and corona. Representative of ≥4 blots. **c**, SILAC proteomics of naïve A549 cells treated with SILAC-labelled particle complexes (18 h of pulse and 4 h of chase) identifies distinct (heavy) proteins delivered to nuclear and cytoplasmic fractions (114 nuclear and 108 cytoplasmic heavy proteins), enriched for mRNA splicing and translation. **d**, NanoLuc in-gel detection of FUS–NanoLuc fusion proteins in lysates from naïve HEK293 cells treated with FUS–NanoLuc–complexes (2 h of pulse and 30 min of chase) detects multiple intact and processed isoforms in cytoplasmic and nuclear fractions. Loaded lysates from varying amounts of particle complexes, 64 μg of nuclear lysate and 46 μg of cytoplasmic lysate. Representative of three experiments. **e**, Live-cell imaging shows AF405-labelled coat condensate depositing on ER ((i); blue: EGFP-calnexin) and mitochondria ((ii); blue: MitoTracker) in HEK293 cells. ER-associated condensate accumulates at the ER–nuclear envelope and then suddenly disperses into the nucleus (170 s onwards). The mitochondrial condensate remains punctate after core loss (10 s onwards) and slowly rearranges and disperses. Observed for multiple times (>10). **f**, Six dynamic coat–P body interaction modes are summarized using AF405-labelled coats from A549-derived particle complexes in HEK293 cells expressing EGFP-DDX6, including transient and prolonged contacts, deposition and intra-P-body dispersion. Trajectories are colour coded by location (cytosol, grey; P body, red). **g**, Trajectory 1 shows coat detachment onto a DDX6 granule and dispersion within the P body (320 s). **h**, Trajectory 3 shows partial detachment reaching the granule core (80, 576 and 608 s) followed by blebbing to form new granules (672 s). Similar events were observed in >5 independent experiments. **i**, EGFP-DDX6 intensity drops after ~800 s for both whole granule and core, consistent with core rearrangement. Source data

Having clarified the architecture and composition of particle complexes produced and exported by cells, we studied if these particle complexes could be part of a programmed pathway of biomolecular and functional transfer. We address this in two complementary ways, depending on whether the condensates remain mesoscopic (fluorescence imaging accessible) or become microscopic and require biochemical assays. First, using bulk imaging and biochemical read-outs after internalization (Figs. 4 and 5a–d), we describe four well-defined post-uptake stages: condensate fluidization, interendosomal sorting, separation from the particle core, and redistribution of condensate cargo between cytosolic and nuclear compartments. When redistributed, they form direct molecular contacts and retain functional activity. Second, using live-cell tracking of mesoscopic condensates (Fig. 5e–i and Supplementary Videos 1–5) and split-reporter strategies, we follow individual separated condensates as they move on cytoskeletal tracks and engage organelles and endogenous RNA granules.

### Condensate detachment and endosomal separation (stages 1 and 2)

By labelling the condensate layer and core separately, we tracked their dynamics independently (Fig. 4a). Shortly after uptake, the condensate fluidizes and detaches from the core. Thereafter, within endosomes, the condensate and core move largely independently, apparently constrained only by the endosomal lumen (for example, see the early time points shown in Fig. 4b(i),(ii)). Without a lipid shell, the melted condensate is directly exposed to the endosomal lumen. Over seconds to minutes, a second phase of interendosomal exchanges and condensate rearrangements is observed (Fig. 4b(iii)), culminating in a partial (Fig. 4b(i)) or complete (Fig. 4b(ii)) separation of condensate from particle-containing endosomes. Thereafter, the separated condensates either persist as resolvable puncta that can be tracked or rapidly disperse below the resolution of fluorescence microscopy. Dispersion often begins as a collective flow along narrow channels (for example, Fig. 4b(ii), *t* = 30–195 s), briefly appearing as a condensate ‘cloud’ before the signal dissipates. If detachment is initially partial, continued interendosomal rearrangements typically lead to complete separation (for example, Fig. 4b(iii)). These processes may reflect progressive interendosomal sorting and rearrangement of condensates until a coordinated recognition threshold enables exit. However, how (or whether) such a threshold operates remains to be established. By the end of stage 2, most core-containing endosomes proceed along the lysosomal route, whereas condensates are released and follow divergent trafficking itineraries. Some separation events appear associated with Rab GTPase scaffolding (for example, Rab5; Fig. 4c). However, if separation is driven primarily by unconventional lumen-facing RNA-granule-like ribonucleoprotein particle (RNP) interactions, our observation that separation kinetics do not easily correlate with conventional endo-lysosomal maturation markers is unsurprising.

The consequences of separation can be quantified by high-content imaging analysis (Fig. 4d,e). Although the mean fluorescence intensities of particle cores and condensate per cell change little over early time points, the condensate intensity associated with particle-containing endosomes decreases appreciably within hours (Fig. 4e(i),(ii)). The evolving shape of the violin plot describing particle-containing endosome-associated condensate intensity (Fig. 4e(iii)) reflects both endosomes that progressively lose condensate and a minority that transiently accumulate more before ultimately losing it, consistent with our description of condensate detachment, interendosomal rearrangements and eventual complete separation.

### Endo-lysosomal departure and redistribution (stages 3 and 4)

Previous work on late-stage biomolecular coronas shows that most nanoparticle coronas traffic to lysosomes and are degraded into peptide fragments over 4–8 h (refs. ^35–38^). By contrast, at late time points (hours to tens of hours), we now find that the condensate is largely separated from the cores and distributed throughout the cytosol and nucleus, with little evidence of degradation. To quantify this difference, we adjusted the condensate and serum-corona labelling so that the condensate protein fluorescence per particle was comparable (Supplementary Fig. 26) and then measured the condensate signal decay using flow cytometry. As expected, serum coronas are almost completely degraded after 6–8 h, whereas the largely detached condensate retains close to its original fluorescence intensity (Supplementary Fig. 27), consistent with high-content analyses. After 22 h, serum-corona signal is nearly undetectable, whereas the condensate remains intact and is distributed between the cytosol and nucleus (Fig. 4f). Cytosolic and nuclear assays (Fig. 5a–d) further confirm serum-corona degradation and condensate persistence. Taken together, these results indicate that condensate architectures largely escape endo-lysosomal degradation.

## Condensate grafting with peptides restores lysosomal degradation

In line with our hypothesis that interaction-governed recognition gates intracellular access, we asked whether departure from the endo-lysosomal pathway could be abolished by progressively disrupting condensate–cell interactions. Although attaching small dyes generally leaves many recognition interactions intact, grafting short peptides onto corona surfaces can qualitatively alter nanoscale recognition^39^. We, therefore, click-grafted ALFA peptides (Supplementary Methods) onto condensate proteins at increasing densities, expecting to obstruct the original condensate–host cell (including luminal) recognition interactions. Under otherwise identical conditions, increasing the ALFA graft density led to progressive condensate degradation after a 2-h pulse and 6-h chase (Fig. 4g, top). Separately masking condensates associated with particle cores (purple, Fig. 4g (bottom)) and condensates that had separated from cores (yellow) showed that as the graft density increased, yellow condensates progressively disappeared in parallel with the loss of endosomal separation events. In essence, higher ALFA densities caused the condensate to remain with the particle cores, abolished departure from the endo-lysosomal pathway and restored conventional serum-corona degradation. These results support a causal link between condensate–host cell molecular interactions, condensate separation from particle-containing endosomes and evasion of terminal lysosomal degradation. Because grafting also changes recognition epitopes at both the plasma membrane and within the endosome lumen, further work will be required to disentangle their relative contributions.

## Condensates reach intracellular targets and remain functional

Having established that the condensate payload escapes the endo-lysosomal pathway, we next examined its intracellular fate. The transferred condensate comprises mRNAs, predominantly RNA-binding proteins, non-coding RNAs and associated RNP assemblies. We tracked these particle-borne RNA granules using three complementary labelling strategies: post-production conjugation, metabolic isotopic labelling and genetic tagging of producer cells by transfection or CRISPR/Cas9. Genetic tagging incorporates both tagged protein and its coding mRNA into particle complexes, enabling the ‘piggybacking’ of functions such as fluorescence or enzymatic activity via precise CRISPR knock-in at selected RNA granule–protein genes. Issues surrounding re-engineering endogenous RNA granule networks and the broader consequences for their transfer are beyond the scope of this Article; therefore, we focus on practically useful modifications (for example, Fig. 5a) and on defining the basic features of RNA granule transfer.

We first developed western blots in which the antibody recognizes the molecular structure of fluorescent dye labels used to ubiquitously post-label particle-complex biomolecules (Fig. 5a and Supplementary Fig. 28), and detected distinct bands for condensate versus serum proteins (right columns labelled P′ and C′; Fig. 5b). Using the same uptake and chase conditions as in Fig. 4f, we then used dye-specific western blots to follow serum (lanes C) and condensate (lanes P) signals in cytosolic and nuclear fractions. Consistent with Fig. 4f, dye-labelled bands from serum-corona proteins were indistinguishable from control in both compartments, indicating near-complete degradation. By contrast, for particle complexes, we detected well-defined condensate bands even at 22 h, well beyond the typical lysosomal timescales, and these bands differed between cytosol and nucleus, suggesting organelle-specific delivery.

We, therefore, prepared isotopically labelled producer cells and confirmed that condensate complexes from them were highly enriched in ‘heavy’ proteins containing Lys (^13^C_6_, ^15^N_2_) and Arg (^13^C_6_). Isotopic labelling allowed condensate-derived proteins to be resolved from the complex cytosolic and nuclear background. In total, 108 heavy proteins were found only in the cytoplasm, 114 only in the nucleus and 23 in both (Fig. 5c), implying selective post-condensate separation trafficking. Network analysis of these fractions revealed protein–protein interaction clusters associated with mRNA splicing, translation and signal recognition particle-dependent targeting (Fig. 5c).

We next asked whether transferred biomolecules remain functional. Using CRISPR/Cas9, we generated particle complexes containing nanoluciferase (NanoLuc)-condensate fusion proteins (for example, FUS; Supplementary Fig. 29). Since NanoLuc bioluminescence has extremely low background (femtomolar detection), we could sensitively monitor the enzyme function transfer. After substrate addition (Fig. 5d; FUS–NanoLuc, 2-h pulse and 30-min chase following magnetic endosome removal), NanoLuc in-gel assays of cytosolic and nuclear fractions revealed discrete bioluminescent bands corresponding to different FUS–NanoLuc isoforms. Similar results with several other NanoLuc knock-in fusions show that intact enzymatic activity can be delivered to recipient cells by condensate-borne cargo.

## Condensate cargo forms molecular contacts in the cytosol

Recipient-cell regulation models require that some cargo reaches the cytosol or nucleus and contacts intracellular targets. We tested for such contact using split NanoLuc complementation, which produces bioluminescence only when two fragments meet at molecular distances. Our full NanoLuc fusion experiments (Fig. 5d) showed robust signals from very small amounts of condensate-borne NanoLuc in recipient cells, indicating sufficient sensitivity to detect rare events. We, therefore, fused LgBiT of NanoLuc to select condensate proteins in producer cells (to generate particle complexes) and expressed the complementary HiBiT peptide in the cytosol of recipient cells. After uptake, bioluminescence above the background is detected only when LgBiT-tagged condensate cargo directly contacts cytosolic HiBiT.

For example, particle complexes from FUS–LgBiT knock-in HEK293 cells were delivered to the target cells expressing HiBiT–V5–Halo. Controls indicated minimal contribution from supernatant bioluminescence (for example, from recycling or export of HiBiT–V5–Halo). To confirm that restored NanoLuc activity arose from viable cytosolic complexes, we also used a cell-permeable pro-substrate that requires intracellular esterases for activation. These data are, therefore, consistent with a small number of FUS–LgBiT molecules within the hybrid condensate cargo, making direct molecular contact with HiBiT (Supplementary Fig. 30). However, split-reporter assays in low-copy fusion proteins embedded in endogenous nanostructures can introduce artefacts that require careful interpretation^40–42^.

Motivated by this evidence consistent with cytosolic access, we examined the fate of transferred mRNA. In an engineered transfer between identical cell types, condensate complexes containing FUS–EGFP mRNA produced by stably expressing FUS–EGFP HEK293 cells were delivered into naïve HEK293 recipients, and reverse-transcription quantitative polymerase chain reaction (PCR) with appropriate primers showed that the exogenous mRNA decayed only over extended times (Supplementary Fig. 31). The kinetic evolution of transferred transcripts, including their length and capacity to be translated, requires further study. More fundamentally, RNA granule architecture, and thus functional delivery, depends on an endogenous, interwoven network of protein, RNA and other nucleic-acid interactions (potentially involving complexes) within the condensate^43^. Engineering isolated network components may, therefore, have unanticipated consequences and will require careful future work^44^.

Finally, global RNA transfer behaviour is more complex. Time-resolved sequencing indicates that the transferred transcripts can exhibit distinct kinetic stabilities (data not shown), consistent with heterogeneous post-transfer fates that may depend on producer/recipient context (for example, cell type) and transcript features (for example, 5′ m^7^G caps, poly(A) tails and regulatory elements).

## Mesoscopic condensates redistribute between organelles

After separating from particle-containing endosomes, some condensates disassembled into microscopic condensates (amenable to the methods above), whereas others remained mesoscopic and could be tracked using fluorescence microscopy. Because standard fixation and permeabilization caused major signal loss (Supplementary Fig. 32), we adopted a milder, condensate-preserving protocol (Supplementary Fig. 33). Separated condensates appeared as puncta that moved along intermediate filaments, microtubules and microfilaments (Supplementary Figs. 34–36). Along these trajectories, they engaged in prolonged contact with other organelles, often ending in abrupt dispersion and apparent merger into the target. In more complex cases, condensates were shuttled between particle, P body and mitochondria before final deposition on mitochondria (Supplementary Fig. 37). The most frequent contacts were with the ER–nuclear envelope (Fig. 5e(i)) and with mitochondria (Fig. 5e(ii)). Early interactions often coincided with pronounced local network remodelling of ER (Supplementary Video 4) and mitochondria (Supplementary Video 5), suggesting considerable local forces. Most striking, a subset of condensates first associated with perinuclear ER, paused at the ER–nuclear envelope boundary (Fig. 5e(i), *t* = 100 s) and then dispersed into the nucleus (Fig. 5e(i), *t* = 170–295 s).

These nuclear-entry events are consistent with high-content imaging (Fig. 4f), nuclear-fraction western blots (Fig. 5b), SILAC proteomics (Fig. 5c) and bioluminescent assays (Fig. 5d), all of which show distinct nuclear signals. In the nucleus, the fluorescent condensate material partially colocalized with Ki-67 (Supplementary Fig. 38), consistent with access to nucleolar and perichromosomal regions. This is consistent with proteomics and RNA-seq, which indicate condensate association with nuclear Sm-like snRNP-rich nuclear condensates (for example, nucleoli and Cajal-body-related structures).

Considering that particle-complex condensates resemble conventional RNA granules, we also examined their interactions with endogenous intracellular DDX6-positive P-body granules. These encounters were typically transient, reversible and sometimes involved multiple partners. Condensates often contacted a given P body several times before depositing a fraction of their material. Figure 5f summarizes six representative cargo-DDX6 time-lapse trajectories. Dwell times were on the order of tens of seconds and usually resulted in partial transfer to the P body (Fig. 5g, *t* = 320 s; Fig. 5h, *t* = 80 s), after which a condensate fragment (by then reduced) departed for other targets. The P bodies themselves also remodelled during these exchanges (Fig. 5i and Supplementary Fig. 39). Some condensate fragments became integrated into the P-body core (Fig. 5h (filled arrow), *t* = 576 s and 608 s), whereas small EGFP-DDX6-labelled structures budded from the highly mobile liquid P-body surface (Fig. 5h (open arrow); *t* = 672 s). As the P bodies are thought to act (partly) as local storage and sorting reservoirs. These observations suggest that particle–condensate cargoes can be delivered to and retrieve components from P bodies during transit. Previous work on P-body interactions with microscopic granules has raised the possibility that such rearrangements are linked to RNA status^45^, but definitive tests will require future study.

## Outlook

We have shown that cell-internalized nanoparticles can, in rare events, acquire an overlaid condensate corona, which allows them to escape lysosomal degradation and be exported into the extracellular space, where they can be internalized by other cells. The majority of trackable particle–condensate complexes in recipient cells follow a common four-stage intracellular processing itinerary. Associated RNAs are mostly intact and functional, with multiple approaches indicating that detectable transcripts are full-length mRNA. These nanoparticle–condensate complexes can make direct molecular contacts consistent with functional delivery. Taken together, our results suggest that the correlated biomolecular composition and architecture of these complex condensates encodes a biomolecular transfer programme, activated by local prompts within recipient cells. It is remarkable that such architectures, built entirely from endogenous biomolecules of producer cells, can embody transfer programmes that overcome most of the challenges faced within nanoscale therapeutics.

The closest known analogue of this behaviour, where condensed RNP granules also directly contact the endosomal lumen, is non-enveloped virus infection, highlighting that endo-lysosomal evasion does not need to rely solely on familiar membrane-breach ‘escape’ routes. In the viral case, RNP granules can reach the cytosol and nucleus via unconventional pathways, including ER-associated retro-translocation from endosome to ER, with only transient cytosolic exposure before nuclear entry. Such unconventional transfer granule programmes remain largely unexplored and could be useful for delivery science.

We are only beginning to explore the full spectrum of higher-level regulatory effects that these transfers exert in recipient cells. Nevertheless, the possibility that intact or partially intact biomolecular machineries involved in translation, splicing and other functions can be transferred raises the prospect that nanoscale granule architectures could move more elaborate, distributed biological functions between cells. Just as viruses move compact functional systems between cells, engineered nanoscale granule organizations could provide a benign counterpart: a platform for modular transfer of entire therapeutic systems, rather than simply individual molecules, between cells.

More broadly, this work highlights a key distinction in approach to bottom-up engineered delivery systems and cell-designed nanoscale architectures that already embody effective design principles. These biological nanostructures have, in effect, evolved (‘learned’) how to use nanoscale recognition to engage host prompts and bypass barriers that bottom-up therapeutic nanoparticles have long struggled to overcome.

We consider it likely that similar biogenesis steps and principled architectures also occur with wholly biologically derived nanostructures. Our synthetic hybrids may, therefore, be revealing a class of unusually protected, long-lived extracellular condensate granules and novel nanoscale processing pathways that are already embedded within living systems.

## Methods

### Biogenesis of particle complexes by live-cell imaging

A549, HEK293 and U87 cells were treated with 800 µg ml^−1^ of mSiO_2_ corona nanoparticles for 10 min, and then washed twice with complete media and twice with phosphate-buffered saline (PBS). The cells were stained with one of the following: CMO (5 µg ml^−1^, 20 min), MitoTracker Red CMXRos (500 nM, 30 min), CellTrace Far Red (2 µM, 20 min). After staining, the dye was removed, and the cells were washed twice with PBS. The cells were incubated in fresh phenol-red-free Minimum Essential Medium and imaged using an Opera Phenix HCS in live-cell mode. The imaging chamber was equilibrated to 37 °C with 5% CO_2_, and images were acquired at 0.33 frames per second (one frame, ~3 s). The same particle exposure and imaging workflow was applied to HEK293 cells transiently expressing EGFP-fusion proteins to visualize the dynamics between EGFP-fusion proteins and biogenesis of particle complexes.

### Production of label-free particle complexes

Approximately 6 × 10^6^ cells were seeded into a T175 flask and cultured for 48 h at 37 °C in a humidified 5% CO_2_ incubator. Nanoparticles were incubated in complete media for 1 h at 37 °C and then 800 µg ml^−1^ of mSiO_2_ was added to the cells. Cells were exposed to 10 ml of nanoparticle suspension for 10 min (pulse). The suspension was removed, and cells were washed twice with complete media and twice with PBS to remove unbound particles. Cells were then incubated in 20 ml of complete medium containing 10% (v/v) fetal bovine serum that had been precleared by centrifugation at 20,000*g* for 30 min, for 4 h (chase). After the chase, the supernatants were collected and clarified at 700*g* for 5 min to remove cell debris. The exported particle complexes were pelleted by centrifugation at 12,000*g* for 40 min at 4 °C. The pellets were resuspended in PBS and subjected to magnetic extraction using our in-house magnetic rack (Supplementary Fig. 4). The resulting particle complexes were resuspended in PBS and quantified by measuring the core-particle fluorescence against a standard curve.

### Production of stable-isotope-labelled particle complexes

The producer cells were first adapted to stable isotope labelling by amino acids in cell culture (SILAC) media following an established protocol^46^. Briefly, HEK293 cells were cultured in SILAC Dulbecco’s modified Eagle’s medium with dialysed fetal bovine serum supplemented with 146 μg ml^−1^ of L-Lys (^13^C_6_, ^15^N_2_), 100 μg ml^−1^ of L-Arg (^13^C_6_, ^15^N_4_) and 100 μg ml^−1^ of unlabelled L-Pro. A549 cells were cultured in SILAC Dulbecco’s modified Eagle’s medium with dialysed fetal bovine serum supplemented with 146 μg ml^−1^ of L-Lys (^13^C_6_, ^15^N_2_), 100 μg ml^−1^ of L-Arg (^13^C_6_) and 100 μg ml^−1^ of unlabelled L-Pro. Once isotopic incorporation exceeded 95% confirmed by proteomics analysis, stable-isotope-labelled particle complexes were produced using the adapted cells as described above, except at the chase phase (4 h) replacing the complete growth medium to SILAC growth medium.

### Proteomic analysis of SILAC particle complexes (A549)

SILAC particle complexes were washed once with PBS by centrifugation and resuspended in 8 M of urea (in 50 mM of NH_4_HCO_3_, 1 mM of EDTA, plus protease inhibitors). Samples were sonicated 2 × 30 s. ProteaseMAX surfactant was added to a final concentration of 0.05% and proteins were reduced with dithiothreitol (DTT; 10 mM, 1 h, room temperature (RT)) and alkylated with iodoacetamide (40 mM, 1 h, RT, dark). Excess iodoacetamide was quenched with DTT (40 mM, 30 min at RT). Proteins were initially digested with lysyl endopeptidase (Lys-C, 1:100 (w/w), 4 h at 37 °C), and then diluted with three volumes of 50 mM of NH_4_HCO_3_ to reduce urea to <2 M and further digested with trypsin (1:100 (w/w), 18 h at 37 °C). The digests were acidified with trifluoroacetic acid (TFA; 0.1% v/v) and clarified (16,000*g*, 10 min). Peptides were desalted using Pierce C18 Tips and resuspended in 2% acetonitrile/0.15% TFA. LC–MS/MS was performed on a Dionex Ultimate 4000 coupled to an Orbitrap Fusion Tribrid MS using a nanoViper Acclaim PepMap 100 column (75 µm × 50 cm, 3 µm, 100 Å). Peptides were separated over 60 min with a linear gradient (solvent A, water + 0.1% TFA; solvent B, 80% acetonitrile/20% water + 0.08% TFA), from 0%–50% B over 45 min and then 50%–90% B over 5 min. MS1 was acquired at a resolution of 120,000 (*m*/*z*, 380–1,500; automatic gain control (AGC), 4 × 10^5^; 50 ms). MS/MS used higher-energy collisional dissociation (28% normalized collision energy; 1.6 *m*/*z* isolation; charge states, 2–7; dynamic exclusion, 60 s) with ion trap rapid scan (AGC, 2 × 10^4^; 35 ms).

### Proteomic analysis and comparison of SILAC particle complexes and EVs (HEK293)

SILAC-adapted HEK293 cells (1.2 × 10^7^) were seeded in T175 flasks and cultured for 24 h, washed twice with prewarmed PBS and incubated for a further 24 h in serum-free medium. Conditioned medium was cleared by centrifugation (2,000*g*, 30 min, 4 °C; then 16,000*g*, 20 min, 4 °C) and EVs were pelleted by ultracentrifugation (160,000*g*, 1.5 h, 4 °C), washed in PBS and repelleted (160,000*g*, 2 h, 4 °C). The final EV pellet was used for proteomics. SILAC particle complexes from HEK293 cells were prepared as described above. Four biological replicates per condition were processed.

Pellets were lysed in SDT buffer (4% sodium dodecyl sulfate, 100 mM of Tris-HCl, 0.1 M of DTT, pH 7.6; 95 °C, 10 min) and clarified (20,000*g*, 10 min). For particle complexes, the remaining pellet was re-extracted in SDT buffer and supernatants were pooled. Proteins were processed by filter-aided sample preparation using passivated 10-kDa-molecular-weight-cut-off filters (0.05% Tween-20 overnight), diluted in 8 M of urea, washed, alkylated with iodoacetamide (50 mM in urea; 30 min, RT, dark) and buffer-exchanged into 50 mM of ammonium bicarbonate. In-filter digestion was performed with trypsin overnight at 37 °C (2 µg per 100 µg protein; for particle complexes, 0.5 µg trypsin per ~200 µg starting material). Peptides were recovered by centrifugation, desalted using Pierce C18 Spin Columns, eluted in 50% acetonitrile/0.1% formic acid, dried (SpeedVac, 45 °C) and reconstituted in 0.1% formic acid. LC–MS/MS analysis was performed on a timsTOF mass spectrometer coupled to an Evosep One system in DDA mode using an 88-min gradient (UCD Proteomics Core Facility).

### Transcriptomic analysis of A549-derived particle complexes

RNA extractions were performed in a Class II biosafety cabinet within a BSL-3 laboratory. Particle complexes were magnetically extracted for 90 min, resuspended in RNase/DNase-free PBS and RNA was isolated using one of the following kits: InviTrap (INVITEK, 1060100300), RNeasy Micro (QIAGEN, 74034), miRNeasy Plasma (QIAGEN, 217204) or miRNeasy Cell (QIAGEN, 217684). RNA yield and integrity were assessed with an Agilent 2100 bioanalyser using RNA 6000 Nano/Pico or Small RNA kits; only samples with RNA integrity number > 7 were used. mRNA-seq (paired end, 150 bp) and small RNA-seq (single end, 50 bp) were performed on an MGISEQ-2000 platform (BGI).

### Isolation and analysis of full-length mRNA from particle complex

Total RNA was extracted from HEK293 cells using the RNeasy Plus Micro Kit (QIAGEN, 74034) and assessed on an Agilent 2100 bioanalyser with the RNA 6000 Pico Kit (Agilent, 5067-1513). Only samples with RNA integrity number > 9 were used. Poly(A)+ mRNA was purified from ~3 µg of total RNA using the RNeasy Pure mRNA Bead Kit (QIAGEN, 180244) under RNase-free conditions (RNaseZap; Invitrogen, AM9780), following the manufacturer’s ‘Purification of PolyA+ RNA from total RNA’ protocol. Input RNA was adjusted to 250 µl with RNase-free water and supplemented with SUPERase•In RNase inhibitor (1 µl, 20 U µl^−1^; Invitrogen, AM2696). Steps 5–10 were performed by centrifugation (≤20,000*g*, RT). To minimize dilution, 20 µl of the first eluate was reapplied for a second elution, and mRNA was stored at −80 °C. mRNA integrity and size distribution were verified with a bioanalyser on the day of extraction (or the following day) using the RNA 6000 Pico and Small RNA kits (Agilent, 5067-1548).

### Generation and amplification of total cDNA

Full-length cDNA was synthesized from 100 ng of total RNA using the SMART-Seq mRNA kit (Takara Bio) under RNase-free conditions (RNaseZap decontamination; Invitrogen, AM9780). Only RNA with RNA integrity number > 9 was used. cDNA synthesis was performed on two biological replicates prepared independently by different operators from separate RNA extractions (Supplementary Fig. 19). A positive control used 100 ng of kit-provided mouse brain total RNA (50 ng µl^−1^; Supplementary Fig. 19b). Double-stranded cDNA was amplified by long-distance PCR (seven cycles), purified with NucleoMag NGS Clean-up and Size Select beads (Macherey-Nagel; Fisher Scientific, 15899167) and assessed on an Agilent 2100 bioanalyser using the High Sensitivity DNA kit (Agilent, 5067-4626).

### Validation of full-length FUS and HNRNPU cDNA by Sanger sequencing

Full-length cDNA amplicons for *FUS* (NM_004960.4) and *HNRNPU* (NM_004501.3) were generated by PCR using gene-specific primers, gel purified (1% agarose) and verified by tiled Sanger sequencing. Primer sequences (5′ → 3′) were as follows.

PCR primers: FUS-For,CGGTACTCAGCGGTGTTGGAAC; FUS-Rev, GGGAACCAGAGGTATAGTTACAATTACATAGTCC; HNRNPU-For, CTCGCGAACTCGGTGAAAGG; HNRNPU-Rev, ATGTACCTGTGGTGCTAATACTAGGC.

Sanger sequencing primers: FUS-For1, GTGGCATGGGCGGAAGTG; FUS-Rev1, TTGCCTCTCCCTTCAGCTTG; HNRNPU-For1, GTGGAGGGGGTAAGCTAAATCA; HNRNPU-For2, GAAGCCATCATGCAAGCCAG; HNRNPU-For3, TCCTTCCCTTGCCTCCCTAA; HNRNPU-For4, AGTCCAAGTGGTAGTGTTTAGCA; HNRNPU-For5, GCTATCCATACCCTCGTGCC; HNRNPU-Rev1, CTGTTCTGTTTTGCCGTCCC; HNRNPU-Rev2, TGCCAAGAATGTTATATTTCCCTGG; HNRNPU-Rev3, ATGTATCAGTTCGTTTTATTTGGGT; HNRNPU-Rev4, AGCTTACAAAGATGGTGGAGTT.

### Transcriptomic analysis and comparison of HEK293-derived particle complexes and EVs

Particle complex and EV pellets were prepared as described above. RNA was isolated in RNase-free conditions using the miRNeasy Micro Kit (QIAGEN, 217084) following decontamination with RNaseZap (Invitrogen, AM9780). Pellets were resuspended in 100 µl of RNase-free PBS (Ambion, AM9624) containing 1% (v/v) SUPERase•In (Invitrogen, AM2696), lysed in 700 µl of QIAzol (QIAGEN, 79306) and processed with chloroform extraction (140 µl; Thermo Fisher, J67241.AP). Phase separation was performed using MaXtract High Density tubes (QIAGEN, 129056; 14,000*g*, 5 min, RT). Large RNA (>200 nt) depleted of small RNAs was recovered per the kit’s Appendix A, and RNA quality/size was assessed using a bioanalyser (Agilent 2100; RNA 6000 Pico Kit, 5067-1513). RNA-seq libraries were prepared from three EV replicates and four particle-complex replicates (50 ng and 100 ng of input RNA, respectively).

Stranded RNA-seq libraries were generated using the Collibri Stranded RNA Library Prep Kit (Invitrogen, A39005024) with rRNA depletion (Collibri H/M/R rRNA Depletion Kit, A39115024). rRNA-depleted RNA was fragmented to ~150-bp inserts, indexed by 13-cycle PCR, and purified with Dynabeads Cleanup beads. Libraries were quality controlled on a bioanalyser using the High Sensitivity DNA Kit (Agilent, 5067-4626) and sequenced as paired-end 100-bp reads on an Illumina NovaSeq 6000 (NU-OMICS DNA Sequencing Research Facility, Northumbria University).

### Labelling proteins in particle complexes with fluorescent dye

Particle complexes and corona–nanoparticle controls were incubated with Alexa Fluor 405 *N*-hydroxysuccinimidyl (NHS) ester (Succinimidyl Ester) or Alexa Fluor 568 NHS ester (Succinimidyl Ester) (Thermo Fisher Scientific) at a w/w ratio of 1:4 dye:corona–nanoparticle or particle complex, at RT for 1 h with gentle shaking. To quench any remaining unreacted dye, glycine was added at a w/w ratio of 1:2 glycine:dye and incubated for 30 min at RT with gentle shaking. The stained mSiO_2_ corona and particle complexes were collected from the dye solution by centrifugation and resuspended in PBS. They were then washed three times in PBS by centrifugation to remove the unbound dye.

### Imaging detachment of the particle-complex coat from the nanoparticle core

A549 and HEK293 cells were incubated with fluorescently labelled particle complexes (40 µg ml^−1^) for defined uptake times, washed twice with PBS and twice with fresh medium to remove unbound complexes and chased in phenol-red-free medium. Live-cell time-lapse imaging was performed on a Nikon Eclipse Ti spinning-disc confocal microscope (×100 oil objective) using a stage-top incubator maintained at 37 °C and 5% CO_2_. Time-lapse data were analysed in Imaris (Bitplane; spot tracking). Coat detachment and spatial distribution were quantified by high-content imaging on an Opera Phenix system (PerkinElmer; 405/488/561/647 nm lasers; ×63 water objective), with image analysis performed in Harmony using experiment-specific segmentation pipelines.

### Functionalization of particle complexes with ALFA peptides

Particle complexes were functionalized with ALFA peptides by copper-free, bio-orthogonal click chemistry using the dibenzocyclooctyne (DBCO)–azide pair. The peptide design (Supplementary Fig. 40) included an *N*-terminal 6-azido-L-lysine for conjugation, a short spacer/proline to preserve ALFA secondary structure and an *N*-terminal carboxyfluorescein (FAM) label for detection. The particle-associated biomolecular layer was first modified with DBCO using DBCO-NHS; the degree of functionalization was tuned by varying the DBCO/nanoparticle ratio (Supplementary Fig. 41 and Supplementary Information). ALFA display on particle complexes was confirmed by flow cytometry following staining with fluorescent anti-ALFA antibodies (Supplementary Fig. 42).

### Nuclear and cytoplasmic fractionation

Nuclear isolation was performed essentially as described in ref. ^47^. Untreated, corona-treated and particle-complex-treated cells were washed with PBS, detached by trypsinization and pelleted (500*g*, 3 min). Pellets were washed once with PBS and resuspended in five pellet volumes of cytoplasmic extraction buffer (10 mM of HEPES, pH 7.4, 2 mM of MgCl_2_, 120 mM of sucrose, complete protease inhibitor). After hypotonic swelling (2 min RT, 10 min on ice), Nonidet P-40 was added to 1% (v/v) and cells were lysed by pipetting. Nuclei were pelleted (700*g*, 3 min, 4 °C) and the supernatant was collected as the cytoplasmic fraction. The nuclear pellet was washed in ten pellet volumes of nuclei wash buffer (10 mM of HEPES, pH 7.4, 5 mM of MgCl_2_, 320 mM of sucrose, 1% of Nonidet P-40, protease inhibitor). To remove particle contamination, the cytoplasmic extracts and resuspended nuclei were subjected to magnetic pull-down at 4 °C overnight. Washed nuclei were repelleted (700*g*, 3 min, 4 °C) and extracted in five pellet volumes of nuclear extraction buffer (20 mM of HEPES, pH 7.9, 1.5 mM of MgCl_2_, 0.42 M of NaCl, 25% glycerol, 1 mM of DTT, protease inhibitor) with vortexing and incubation (30 min, 4 °C, shaking). Soluble nuclear proteins were collected after centrifugation (20,000*g*, 20 min, 4 °C) and stored at −80 °C. Fraction purity was verified by immunoblotting for histone H2B (Supplementary Fig. 43).

### Western blot detection of AF405-labelled proteins delivered by particle complexes

A549 cells were treated with AF405-labelled A549-derived particle complexes (80 µg ml^−1^; 18 h of pulse and 4 h of chase). Nuclear and cytoplasmic fractions were prepared as described above and protein concentrations determined by bicinchoninic acid assay. Equal amounts of protein for particle-complex-treated and corona-treated cell lysate (60 µg nuclear; 86 µg cytoplasmic) and input controls (AF405-labelled particle complexes and corona) were resolved on 4%–12% sodium dodecyl sulfate–polyacrylamide gel electrophoresis (120 V) and transferred to polyvinylidene difluoride membranes (100 V, 60 min). Membranes were blocked for 1 h at RT in TBST (0.1% Tween-20) containing 5% bovine serum albumin and incubated overnight at 4 °C with an anti-AF405 primary antibody (1:3,000). After three washes in TBST (15 min each), membranes were incubated with HRP-conjugated secondary antibody (1:5,000, 1 h, RT), washed three times, developed using enhanced chemiluminescence and imaged on a Gel Doc EZ system (Bio-Rad).

### Mass spectrometry analysis of SILAC proteins delivered by particle complexes

A549 cells were treated with SILAC particle complexes (generated from SILAC A549 cells) at 100 µg ml^−1^ for an 18 h of pulse followed by 4 h of chase. Nuclear and cytoplasmic fractions were prepared as described above. For each fraction, 100 µg of protein was processed by filter-aided sample preparation, and the resulting peptides were quantified by NanoDrop for sample normalization. LC–MS/MS was performed on a Dionex Ultimate 3000 coupled to a Q Exactive quadrupole-Orbitrap (Thermo Fisher Scientific) using a C18 in-house column (75 µm × 15 cm, 3 µm, 100 Å). Peptides from ‘heavy-labelled particle-complex’ samples were separated over 60 min (solvent A: 2.5% acetonitrile/0.5% acetic acid; solvent B: 97.5% acetonitrile/0.5% acetic acid) with 2%–25% B over 58 min; cytoplasmic and nuclear samples were separated over 120 min with 2%–28% B. Data were acquired in DDA (top 12). MS1 settings: resolution, 70,000; *m*/*z*, 350–1,600; AGC, 3 × 10^6^; maximum injection time, 60 ms. MS/MS used higher-energy collisional dissociation (27% normalized collision energy; 2 *m*/*z* isolation; charge, 2–7; dynamic exclusion, 30 s) with resolution of 17,500, AGC of 5 × 10^4^ and maximum injection time of 250 ms.

### NLuc in-gel detection of NLuc fusion protein transferred by particle complexes

Particle complexes were generated from FUS–NLuc–α knock-in cells using the same conditions described for A549-derived particle complexes. HEK293 cells were treated with these particle complexes (50 µg ml^−1^; six-well plates) for 2 h, washed and incubated in fresh medium for 30 min. Cells were then washed twice with cold PBS, harvested by trypsinization, and cytosolic and nuclear fractions were prepared. Equal amounts of protein for treated and untreated cell lysates (64-µg nuclear and 46-µg cytoplasmic) and input controls (lysates from varying amounts of FUS–NLuc-containing particle complexes: 20, 10 and 5 µg) were resolved on 10% sodium dodecyl sulfate–polyacrylamide gel electrophoresis as described above. Gels were washed twice with 25% isopropanol and twice with water to facilitate NLuc refolding, incubated with Nano-Glo Live Cell Assay reagent (prepared as per manufacturer’s instructions) for 3 min in the dark and imaged for bioluminescence using a gel documentation system.

### LC–MS data analysis

Raw files were processed in MaxQuant (v. 2.0.1.0; Andromeda) against the UniProt Homo sapiens database (UniProt Homo sapiens [9606]-203800-202201.fasta). Trypsin/P was specified with up to two missed cleavages.

For SILAC A549 particle-complex data acquired on the Orbitrap, precursor and fragment tolerances were set to 20 ppm and 0.5 Da, respectively. Carbamidomethylation and SILAC labels (Lys8: ^13^C_6_, ^15^N_2_; Arg6: ^13^C_6_) were set as fixed modifications; oxidation (M), deamidation (N/Q) and protein *N*-terminal acetylation were set as variable modifications. Peptide spectrum match and protein false-discovery rate were controlled at 1%. Protein quantification used razor and unique peptides with label-free quantification and ‘match between runs’ enabled.

For SILAC HEK293 particle-complex and EV data acquired by timsTOF DDA, raw files were analysed using a single ‘heavy-only’ SILAC setup (multiplicity, 1) with Arg10 (^13^C_6_, ^15^N_4_) and Lys8 (^13^C_6_, ^15^N_2_), and protein abundances were estimated by label-free quantification with ‘match between runs’ enabled.

Downstream analyses were performed in R (v. 4.5.1) using log_2_-transformed label-free quantification intensities. *Z*-score heat maps were generated with pheatmap (v. 1.0.13), principal component analysis was performed in R, missing values were imputed with missForest (v. 1.5) and differential abundance was assessed with limma (v. 3.64.3). Gene Ontology enrichment was performed with Metascape^48^ and visualized using ggplot2 (v. 3.5.2). Circular plots of multilocalized proteins were generated with PANTHER^49^ and the circlize R package^50^ using the Human Protein Atlas database^27^.

### RNA-seq data analysis

Base calling outputs were demultiplexed and quality-filtered with bclfastq2 (v. 2.20; default settings) to generate FASTQ files. Reads were aligned to the human reference genome GRCh38 (GRCh38.p14.genome.fa) using HISAT2 (v. 2.1.0)^51^, and gene-level counts were generated with featureCounts. Downstream analyses were performed in R (v. 4.5.1): principal component analysis and heat maps (pheatmap (v. 1.0.13)), differential expression and volcano plots (DESeq2 (v. 1.48.2)) and additional visualizations (ggplot2 (v. 3.5.2))^52^. Gene Ontology enrichment was performed with clusterProfiler (v. 4.16.0). Small RNA-seq biotype profiling was carried out with exceRpt^53^ and visualized in GraphPad Prism 9. Biological replicates showed high reproducibility across operators (Supplementary Fig. 44).

### Statistics and reproducibility

All the datasets are highly reproducible, and (unless otherwise stated) contain at least three replicates, but the comparisons and cross-checking by multiple researchers over time ensures a much higher level of reproducibility than that stated. No statistical method was used to predetermine the sample size. No data were excluded from the analyses.

### Reporting summary

Further information on research design is available in the Nature Portfolio Reporting Summary linked to this article.

## Online content

Any methods, additional references, Nature Portfolio reporting summaries, source data, extended data, supplementary information, acknowledgements, peer review information; details of author contributions and competing interests; and statements of data and code availability are available at 10.1038/s41563-026-02534-5.

## Supplementary information

Supplementary Information Supplementary Figs. 1–45, Methods and captions to Supplementary Videos.

Reporting Summary

Supplementary Video 1 Live-cell imaging of releasing internalized FITC-polystyrene nanoparticles (PS-COOH, 100 nm in diameter) from A549 cells. Red,CMO cell membrane staining; green, FITC PS-COOH nanoparticles. The PS-COOH nanoparticles were purchased from ThermoFisher Scientific (catalogue number F8803).

Supplementary Video 2 Live-cell imaging of releasing internalized mSiO_2_ nanoparticles from A549 cells. Red, CMO cell membrane staining; green, FITCmSiO_2_ nanoparticles.

Supplementary Video 3 Live-cell imaging of releasing internalized ApoE-functionalized mSiO_2_ nanoparticles from U87 cells. Red, CMO cell membranestaining; green, FITC ApoE-mSiO_2_ nanoparticles.

Supplementary Video 4 Live-cell imaging of melting and sorting a particle-complex coat along the ER network. Particle complexes were derived fromA549 cells. The particle-complex coat was labelled with NHS-AF405, and the particle core was labelled with Cy5. A549 cellswere treated with the particle complexes at 40 μg ml^−1^. Red, AF405-labelled particle-complex coat; green, Cy5 mSiO_2_ particles;blue, EGFP-calnexin.

Supplementary Video 5 Live-cell imaging of melting and sorting particle-complex coat in the mitochondrial network. Particle complexes were derivedfrom A549 cells. The particle-complex coat was labelled with NHS-AF405, and the particle core was labelled with FITC. A549cells were treated with the particle complexes at 40 μg ml^−1^. Red, AF405-labelled particle-complex coat; green, FITC mSiO_2_ particles; blue, MitoTracker.

## Source data

Source Data Fig. 2 Statistical source data.

Source Data Fig. 3 Statistical source data.

Source Data Fig. 4 Statistical source data.

Source Data Fig. 5 Statistical source data.

## Data Availability

The data supporting the findings of this study are available within the article and its Supplementary Information. Imaging data are available via Zenodo at 10.5281/zenodo.18473314 (ref. ^54^). The mass spectrometry proteomics data have been deposited in the PRIDE repository with dataset identifier PXD071779. The RNA-seq data have been deposited in the EMBL-EBI repository with ArrayExpress accession E-MTAB-16398. Source data are provided with this paper.
