# Supplementary material for: Condensate corona–nanoparticle complexes transfer functional biomolecules between cells
Source: Nat Mater. 2026 Apr 16;25(6):1045–57. doi: 10.1038/s41563-026-02534-5 (PMC13236592; doi:10.1038/s41563-026-02534-5)
Supplement: Supplementary file 1 — Supplementary Figs. 1–45, Methods and captions to Supplementary Videos. [file 41563_2026_2534_MOESM1_ESM.pdf]

nature materials

Supplementary information https://doi.org/10.1038/s41563-026-02534-5

Condensate corona–nanoparticle complexes transfer functional biomolecules between cells

In the format provided by the authors and unedited

Supplementary Methods

Materials

Fluorescein 5-isothiocyanate (≥90%); tetraethyl orthosilicate (reagent grade, 98%), (3 Aminopropyl)-trimethoxysilane (97%), ferric chloride hexahydrate (≥99%), ferrous chloride tetrahydrate (≥99.0%), oleic acid (technical grade, 90%) and cyclohexane (99.5%) were purchased from Sigma-Aldrich. Ethanol (99.8%, HPLC grade), acetone (Reagent grade, 99%), hydrochloric acid 37% w/w (ACS reagent, Honeywell Fluka™) and ammonia (35 % w/w) were purchased from Fisher scientific. Formaldehyde (HT501128), urea (U5378), 40% acrylamide/bis-acrylamide solution (A7802), trizma base (T1503), ammonium persulfate (APS, A3678), N,N,N,N-tetramethylethylenediamine (TEMED, T9281), Triton X-100 (T9284), Tween 20 (P1379), skim milk powder (70166), glycine (G8898), Sucrose (50389), sodium dodecyl sulfate (SDS, L3771), ethylenediaminetetraacetic acid (EDTA, E4884), and DL-Dithiothreitol (DTT, D5545) were all purchased from Merck-Sigma Aldrich. Ethanol (32294) and methanol (34860) were purchased from Honeywell.

Antibodies

Rabbit anti-Glyceraldehyde-3-phosphate dehydrogenase (GAPDH) antibody (14C10) (1:1000 for WB), mouse anti-Lamin A/C antibody (4C11) (1:100 for IF), Rabbit anti-NUP98 antibody (C39A3) (1:400 for IF) were purchased by Cell Signalling technologies.

Rabbit anti-Ki67 antigen (Ki67) antibody (ab92742) (1:500 for IF), Rabbit anti-Lysosomal associated membrane protein 1 (LAMP1) antibody (ab24170) (1:250 for IF), Mouse anti- Vimentin antibody (ab20346) (1:200 for IF), Rabbit anti-Rab11 antibody (ab3612) (1:100 for IF), Rabbit anti-beta COP antibody (ab289) (1:2000 for IF), Rabbit anti-REEP5 antibody (ab167405) (1:200 for IF), Mouse anti-TOMM20 antibody (ab56783) (1:500 for IF), HRP-functionalised Goat anti-Mouse IgG (ab97023) (1:2000 for WB) and HRP-goat anti-Rabbit IgG (ab6721) (1:2000 for WB) were purchased from Abcam.

Anti-alpha Tubulin antibody (236-10501) (1:200 dilution for IF), AlexaFluor® 546-functionalised Goat anti-Rabbit IgG (A11035) (1:1000 for IF), AlexaFluor® 546-functionalised Goat anti-Mouse IgG (A11003) (1:1000 for IF), Rabbit Anti–Alexa Fluor 405/Cascade Blue Dye antibody (A5760) (1:1000 for WB; 1:200 for IF), anti-calnexin antibody (GT1563) (1:500 for IF) were purchased from Thermofisher.

Vectors and stable cell lines

EGFP-G3BP1, EGFP-HNRNPR and EGFP-HNRNPU expressing vector was gateway cloned by LR ligation of destination vector pDEST-CMV-N-EGFP (Addgene, cat. No. 122842) and pENTR-G3BP1

(Addgene, cat. no. 38067), pFRT/TO/HIS/FLAG/HA-HNRNPR (Addgene, cat. no. 127104) or pFRT/TO/HIS/FLAG/HA-HNRNPU (Addgene, cat. No. 38068) respectively.

For Calnexin-EGFP and mcherry-Rab5a expressing vectors, attB flanked calnexin or rab5a encoding sequence was firstly PCR amplified using pCalN-ddGFP-A (Addgene, cat. no. 40290) or pLEX-FKBP-Rab5-Blasticidin (Addgene, cat. no. 120715) as the template, and then gateway cloned by BP ligation with pDONR221 (Thermo Fisher Scientific). The expressing vector was then achieved by LR ligation of the entry clones from last step and pDEST-CMV-C-EGFP (Addgene, cat. No. 122844) or pDEST-CMV-N-mcherry (Addgene, cat. No. 123215), respectively.

HEK293 cell line stably expressing EGFP-G3BP1, EGFP-HNRNPR, EGFP-HNRNPU, EGFP-DDX6 (Addgene, cat. 25033) or EGFP-FUS (Addgene, cat. 60361) was achieved by transient transfection (Fugene 6, Promega) following by antibiotic selection with G418 at 0.6 mg/mL until the cells reached a normal growth speed. The cells were then sorted using flow cytometer to get the EGFP positive populations.

Synthesis of magnetic multicore fluorescent silica shell nanoparticles

The magnetic multicore encapsulated in the fluorescent silica shell were synthesized following a previously described procedure. Briefly, magnetite nanoparticles were synthesized by coprecipitation and then coated with oleic acid to form a stable dispersion in cyclohexane. Then, the magnetite nanoparticles were assembled to form multicores by evaporating an emulsion of the organic ferrofluid in an aqueous solution of sodium dodecyl sulfate prepared by sonication. The prepared multicores were then consolidated by the formation of a silica shell following a sol-gel process in a basic hydro-alcoholic media using Tetraethyl orthosilicate as precursor of the silica. The fluorescent silica shell was then grown in similar conditions, by adding a conjugate of FITC with an aminosilane to incorporate the dye within the silica matrix. In order to obtain endotoxin free nanoparticles, the dispersion of particles was first sterilized by adding ethanol to reach ratio of 70% ethanol, 30% water (v/v), and the particles were then washed twice with LPS free water. Finally, under aseptic working conditions, using pyrogen free consumable and glassware, a dense layer of silica was grown at the surface of the particles in an aqueous dispersion to reduce the leakage of the dye over time in biological media due to silica dissolution.

The endotoxin level of all finished batches was measured with the Pierce LAL Chromogenic Endotoxin Quantitation Kit (Cat. no. 88282) according to the manufacturer's instructions. Particle dispersions (0.8 g/L) were incubated at 37 °C with limulus amebocyte lysate (LAL), followed by incubation with the chromogenic substrate, and absorbance was recorded at 405 nm. A standard curve (0.1–1 EU/mL) and matrix-matched blanks containing nanoparticles (to control for optical/assay interference) were run in parallel. Potential interference was evaluated by spiking known amounts of LPS into nanoparticle-containing samples to confirm recovery.

Colloidal characterisation

Size distribution of the nanoparticles was determined by DLS measurement after dispersion in water, differential centrifugal sedimentation (DCS) in a sucrose gradient (8-24% w/w in water), considering a colloidal SiO2 density of 2 g/cm3 (in the case of the composite magnetic particles the same density was considered meaning that the measured size doesn't correspond exactly to the real size), and transmission electron microscopy (TEM). Measurement of the zeta potential of the nanoparticles dispersed in HEPES 5 mM pH 7.4 was performed on a Malvern Zetasizer ZS series. In the case of the particles coated with biomolecules (Corona, Particle complex), the DLS and the DSC were performed in PBS.

Negative staining protocol for transmission electron microscopy (TEM)

After magnetic pool down, the samples were fixed with 4% Formaldehyde (in 0.1 M sodium cacodylate buffer, pH=7.4) for 90 min at RT in the dark for *Staining n°1* or a mixture of 2.5% Glutaraldehyde + 2% Paraformaldehyde (in 0.1 M phosphate buffer, pH=6.4) for 90 min at 4 °C in the dark for *Staining n°2*. Formar/Carbon Copper grids (200mesh) were hydrophilized (PELCO easiGlow™, negative charge, 1 min, 1 mA) and were immediately incubated with the fixed sample (0.4 µg) for 15 min. The grids were washed six times with Milli-Q water (to remove any salt residue) prior to negative staining with 2% Uranyl acetate for 2 min for the *Staining n°1* or 1% Uranyl acetate + 1% (or 0.1% when indicated) Trehalose for 2 min for *Staining n°2*. The last step was performed on ice and protected from light. The grids were left to air dry and convectional TEM images were obtained on a JEOL 2100 LaB TEM or FEI Tecnai G2 20 Twin both operating at 200 kV.

Scanning electron microscopy (SEM)

SEM imaging was done on Silicon wafers (cleaned using sonication in soapy water, acetone and ethanol, after which the wafer was blow-dried and treated in glow-discharge for 5 minutes).The wafers were placed in a well plate where target cells, after reaching 70% confluency, were incubated with the nanoparticle, corona or complexes for 10 minutes, after which the cells were washed in PBS and fixed using a warm 4% Formaldehyde/2.5% Glutaraldehyde mixture. A dehydration procedure was done using steps of 30%,50%,70%,90%,100% ethanol, and finally HDMS. The wafers were then dried naturally, coated with 12 nm Iridium and imaged in a Zeiss Sigma 300 SEM using an Inlens detector.

Cell culture

A549 non-small lung carcinoma (ATCC® CCL-185), and HEK-293 (ATCC® CRL-1573) cell lines were purchased from ATCC. A549s were cultured in MEM supplemented with 10% (v/v) FBS, 50 units/mL penicillin and 50 µg/mL streptomycin; referred to hereafter as cMEM. HEK-293 cells were cultured in DMEM supplemented with 10% (v/v) FBS, 50 units/mL penicillin and 50 µg/mL streptomycin; referred to hereafter as cMEM Cells were grown at 37˚C in a humidified atmosphere of 5% CO_2 and sub-cultured at 70-80% confluence using trypsin (0.05% in EDTA). Cells were screened

monthly for mycoplasma contaminations using the MycoAlert™ Mycoplasma Detection Kit (Lonza), and all cultures were free of contamination for the duration of experiments reported.

Lipid staining

Particle complex and corona-NP controls (2 µg/ml) were incubated with CellMask Orange Plasma membrane Stain (Thermo Fisher Scientific; 5 µg/ml working concentration) for 30 min at 37°C. The complex and corona-NPs were collected by centrifugation and washed by resuspension in PBS and centrifugation. The corona-NP and complex pellets were finally resuspended in 0.22 µm filtered PBS, for analysis by flow cytometry and microscopy.

Flow cytometry

Nanoparticles and Particle Complexes were analysed using a Beckman Coulter CytoFLEX LX flow cytometer equipped with 6 lasers (UV-Violet-Blue-Yellow-Green-Red). Data was analysed using the CytExpert 2.3.0.84 and FlowJo™ X 10.0.7r2 software. For nanoscale detection, the filters were configured so that the Violet SSC (VSSC) 405/10 nm channel served as the trigger channel to discriminate the noise, and the FITC channel was used to discriminate the core particle population (Gating strategy is illustrated in Supplementary Figure 45).

Immunocytochemistry

For immunocytochemical localisation of lamina A/C and Ki67, the cells were firstly fixed with a 4 % v/v paraformaldehyde (PFA) solution for 15 minutes at room temperature. They were then permeabilised with a 0.1% v/v Triton-X100 solution for 10 minutes at room temperature, followed by blocking with a 1 % w/v bovine serum albumin (BSA) solution for 1 h at room temperature. The samples were then incubated for 1 h with the primary antibody (2 to 5 µg/ml, in a 1 % w/v BSA solution), washed twice and incubated for 1 hour at RT with the secondary antibody (2 µg/ml, in a 1 % w/v BSA solution). All the steps were carried out keeping the samples protected from light.

Immunocytochemistry with optimised permeabilization

To preserve the Particle Complex cargo during immunocytochemical localisation experiments, a mild permeabilization protocol was developed to limit the detergent-induced dissolution that was observed using the standard (previously described) Triton-X100 permeabilization method. The cells were washed in PBS and fixed with 4 % v/v PFA for 15 min at room temperature, followed by two washes with fresh PBS. The permeabilization was performed with a 0.1 % v/v saponin solution in a 1 % w/v BSA solution for 10 min. The samples were then incubated for 1 h with the primary antibody (2 to 5 µg/ml, in 1 % w/v BSA), washed twice in BSA and incubated for 1 h at room temperature with the secondary antibody (2 µg/ml, in 1 % w/v BSA). All the steps were carried out keeping the samples protected from light.

Fixed cell imaging

Fixed cell imaging experiments were carried out in glass bottom 96-well plates, seeded 24 h prior with 5,000 cells per well. The cells were stained with DAPI (2 μg/ml) for 15 min at RT, or with AlexaFluor® 594-conjugated wheat germ agglutinin (5 μg/ml) for 20 min at RT, followed by immunostaining as previously described.

Functionalisation of the particles with DBCO

The stock of Freshly prepared particle complex and corona was centrifuged at 12000 × g for 12 min at 4°C and resuspended in PBS at a concentration of 1 g/L. 150 μL was then used for each condition, and the appropriate amount of Dibenzocyclooctyne-N-hydroxysuccinimidyl ester freshly diluted in 150 μL of PBS was clickily added and the mixture was homogenised by pipetting. The calculation of the ratio of DBCO per particles was done using a particle diameter of 140 nm and a density of 2 $g.cm^{-3}$. The mixture was left to react at room temperature for 1h, protected from the light.

Labelling particle complex with ALFA peptide

Excess of DBCO-NHS (and dye-NHS) was blocked by adding glycine to a final concentration of 1 mM. After 10 min, a solution of ALFA peptide at 5 g/L in DMSO was added to the particles to obtain a ratio of 5×10^4 Peptide/NP. The mixture was kept at room temperature for 1h protected from the light. Finally, the particles were washed 3 times by centrifugation (12 min, 12000 × g, 4°C) with 500 μL of PBS, and resuspended in 20 μL of PBS for further use. The concentration of the particle complexes or corona particles was measured by fluorescence, using a standard curve prepared with the bare particles.

LC-MS/MS lipidomic analysis of particle complexes and extracellular vesicles

Extracellular vesicles (EVs) and particle complexes derived from HEK293 cells were collected and resuspended in PBS. Particle concentration was determined using Nanoparticle Tracking Analysis (Malvern NanoSight NS300) with five repetitions of 60 seconds each. A total of 2×10^{10} particles per sample were used for downstream lipidomic analysis.

To facilitate lipid extraction, the particle dispersion was treated with 1 mg/mL proteinase K at 55°C (1000 rpm) for 30 min to digest the protein coat and release lipids into solution. To minimize lipid oxidation, the sample tube was flushed with nitrogen (N_2) before sealing and enzymatic treatment. Following digestion, the sample was dried using an Eppendorf® Concentrator 5310 (Function 3, 60°C). Derivatization solution (50 μL) was added to the dried sample and incubated for 25 min before being dried again under a nitrogen stream for 60 min. Lipids were then extracted using ammonium acetate (19 mg in 50 mL HPLC-grade methanol) and centrifuged at 500 × g for 2 min. For LC–MS/MS analysis, 150 μL of the eluate was mixed with an equal volume (150 μL) of HPLC-grade water. Additionally, for

flow injection analysis tandem mass spectrometry (FIA-MS/MS), 50 μL of eluate was added to the running solvent. The analysis was conducted using a Sciex ExionLC series UHPLC system coupled with a Sciex QTRAP 6500+ mass spectrometer. A UHPLC column from Biocrates Life Sciences (Innsbruck, Austria) was used, with mobile phases consisting of 100% water (phase A) and 95% acetonitrile (phase B), both containing 0.2% formic acid.

Lipids were identified and quantified in micromolar concentrations using the multiple reaction monitoring (MRM) method. Data quality was assessed by evaluating the accuracy and reproducibility of quality control samples provided with the Quant 500 and P180 assays. Two biological replicates (2×10^{10} particles each) were performed for both particle complexes and EVs.

Generation of FUS-NLuc-α knock-in HEK293 cells using CRISPR Cas9

HEK293 cells were cultured in Dulbecco's Modified Eagle's Medium (DMEM) supplemented with 10% fetal bovine serum (FBS), 100 units/mL penicillin, and 100 μg/mL streptomycin in a humidified atmosphere at 37 °C with 5% CO_2. Single-guide RNA (sgRNA) targeting the stop codon site of FUS (ID: 2521) was designed using an online CRISPR design tool (e.g., CRISPOR, CCTop). The sgRNA sequence (5'-GGGAGCCAGGCTAATTAATA-3') was cloned into a vector containing the Cas9 nuclease through BbsI site. A donor template was synthesized containing left and right homology arms (~800 bp each) flanking nanoluciferase-ALFA sequence (NLuc-α).

HEK293 cells were seeded in a 6-well plate at 3×10^5 per well 24 h before transfection. Transfection was performed using FuGENE 6 according to the manufacturer's instructions. Each well was transfected with 1 μg of sgRNA-Cas9 plasmid and 2 μg of donor template DNA.

After transfection and cell expansion, the cells were seeded at low density to obtain single-cell derived colonies. Colonies were expanded and screened for correct integration of the donor template by bioluminescence assay, PCR and subsequence PCR product sequencing. The incorporation of FUS-NLuc-α fusion proteins were confirmed by western blot and bioluminescence assay (Supplementary Figure 29).

Generation of FUS-LgBiT knock-in HEK293 cell line using CRISPR Cas9

HEK293 cells were cultured in DMEM supplemented with 10% fetal bovine serum (FBS), 100 units/mL penicillin, and 100 μg/mL streptomycin in a humidified atmosphere at 37°C with 5% CO_2. Single-guide RNA (sgRNA) targeting the stop codon site of hFUS (ID: 2521) was designed using an online CRISPR design tool (Chopchop, CCTop). The sgRNA sequence (5'-GGGAGCCAGGCTAATTAATA-3') was cloned into a vector containing the Cas9 nuclease through BbsI site. A donor template was cloned into pUC19 vector containing left and right homology arms (~800 bp each) flanking gene sequence.

HEK293 cells were seeded in a 6-well plate at 200, 000 per well 24 h before transfection. Transfection was performed using FuGENE 6 according to the manufacturer's instructions. Each well was transfected with 1 μg of sgRNA-Cas9 plasmid and 2 μg of donor template DNA. After transfection for 3 d, cells were seeded into 96 well plate with an average of 0.5 cell per well for single clone selection. Genomic DNA were extracted and PCR amplified after the single clone formation to validate the successful knock-in of LgBiT into FUS gene.

Reconstitution of Nanoluciferase by FUS-LgBiT Particle Complexes in HiBiT-Expressing Recipient Cells

Particle complexes were prepared from either FUS-LgBiT knock-in cells or wild-type HEK293 cells under identical conditions. HEK293 cells were seeded in a 96-well plate at a density of 4,000 cells per well.

Two distinct experimental settings were employed for nanoluciferase reconstitution, differing in the sequence of particle complex treatment and HiBiT-V5-HaloTag plasmid transfection:

Setting 1: After 24 hours of seeding, cells were first transfected with HiBiT-V5-HaloTag plasmid (0.2 μg/well) using Fugene 6 for 16 h. The transfection media were then removed, and cells were washed with PBS and rested for 6 h. Subsequently, the cells were treated with particle complexes (200 μg/mL) for 16 h. Following treatment, the media were replaced with fresh media, and cells were incubated for an additional 12 h.

Setting 2: After 24 hours of seeding, cells were first treated with particle complexes (200 μg/mL) for 16 h. The treatment media were removed, and cells were then transfected with HiBiT-V5-HaloTag plasmid (0.2 μg/well) using Fugene 6 for 24 h. After transfection, the media were replaced with fresh media, and cells were incubated for another 24 h.

Finally, Nano-Glo® Live Cell Assay substrate was added following the manufacturer’s protocol, and bioluminescence was measured using a plate reader.

Supplementary Figures

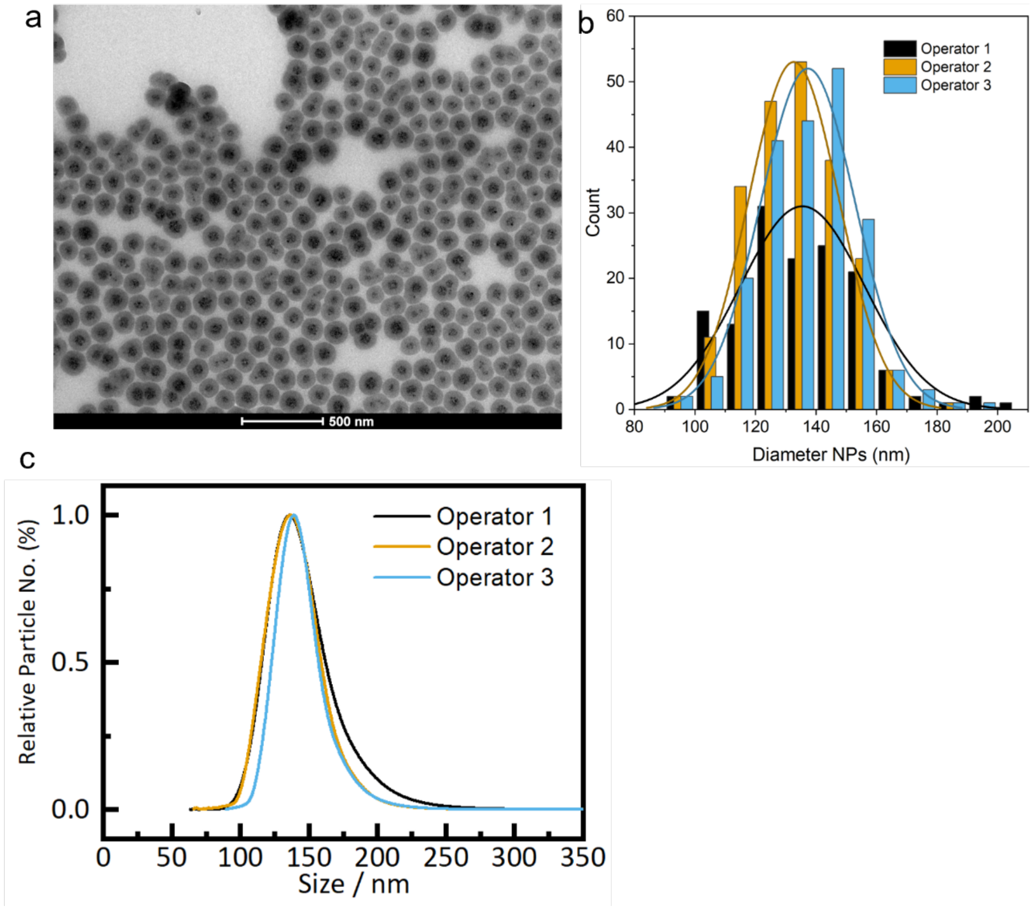

Supplementary Figure 1. Size characterisation and batch-to-batch reproducibility of $mSiO_2$ NPs. a) Representative TEM images of the $mSiO_2$ particles illustrating the magnetic multicore encapsulated in a silica shell. b) Size distribution of 3 independent batches, based on manual counts of particles in ImageJ software from (n ≥ 140 total), and corresponding Gaussian distribution fits. The mean and standard deviation for the batches from operator 1, 2 and 3 are 136 ± 21, 133 ± 15 and 137 ± 16, respectively. c) The average particle size for the same 3 independent batches is ca. 140 nm by DCS, in good agreement with TEM measurements and demonstrating good reproducibility between separate batches by independent operators.

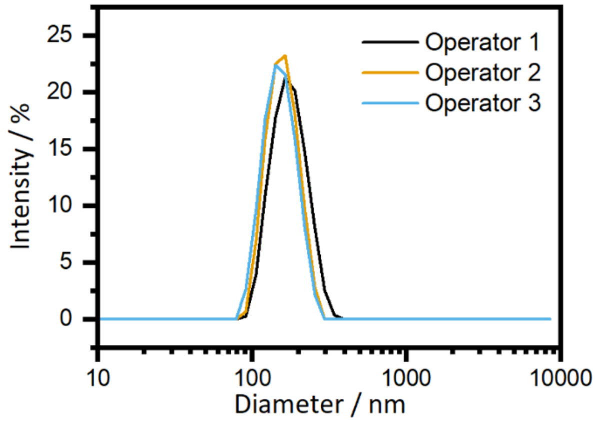

Sample	Z-Ave (nm)	PDI	Zeta Potential (mV)
Operator 1	168 ± 4	0.04 ± 0.02	-38 ± 2
Operator 2	153 ± 1	0.03 ± 0.02	-41 ± 1
Operator 3	148 ± 1	0.02 ± 0.02	-38 ± 1

Supplementary Figure 2. Colloidal dispersion of mSiO_2 NPs colloidal stability for the particles was assessed via DLS measurements. DLS measurements for the same independent batches show that the particles are all of a similar hydrodynamic size around 150-170 nm; the hydrodynamic size is larger than the true diameter determined from TEM, as expected due to the solvation of the particles, but also due to the stronger contribution of the larger particles in the measured scattered intensity. Zeta potential measurements of the particles in 5 mM HEPES pH 7.4 also indicate that the particles have a large negative zeta potential value attributed to the surface silanolate groups. Average of 3 measurements ± SD.

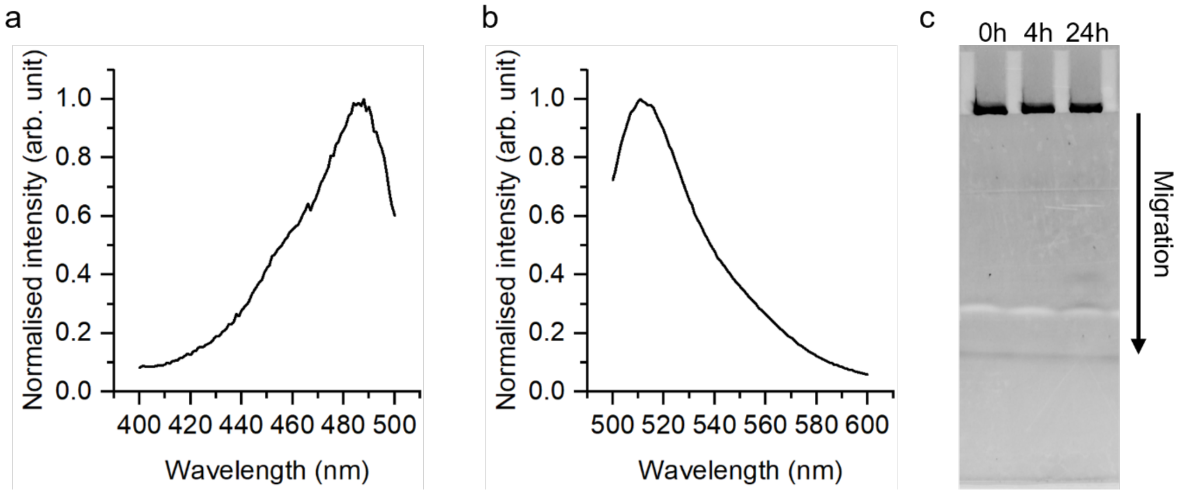

Supplementary Figure 3. Fluorescence characterisation of $mSiO_2$ NPs. Typical excitation (a) and emission (b) spectra of $mSiO_2$ NPs at a concentration of 0.5 g/L in water. The excitation peak maximum of 488 nm when measuring emission intensity at 515 nm and emission peak maximum at *ca.* 511 nm when measuring excitation at 488 nm are typical of FITC dye and indicate that the dye was successfully incorporated into the silica shell. c) Dye leaking test: to both ensure dye stability and verify that incorporated dye does not leach from the particles under experimental conditions, $mSiO_2$ particles were immersed in cMEM at a concentration of 0.1 g/L and incubated at 37°C for different times, to simulate incubation conditions for the particles in cell exposure experiments. Subsequent dilution of exposed particles in SDS loading buffer and PAGE at 130 V for 45 min in 10% acrylamide gel was used to test for dye leakage, as the particles are too large to enter the gel matrix. No FITC dye leakage from the particles could be observed and all detected fluorescence (in black in the figure) was co-localised with particles in the wells.

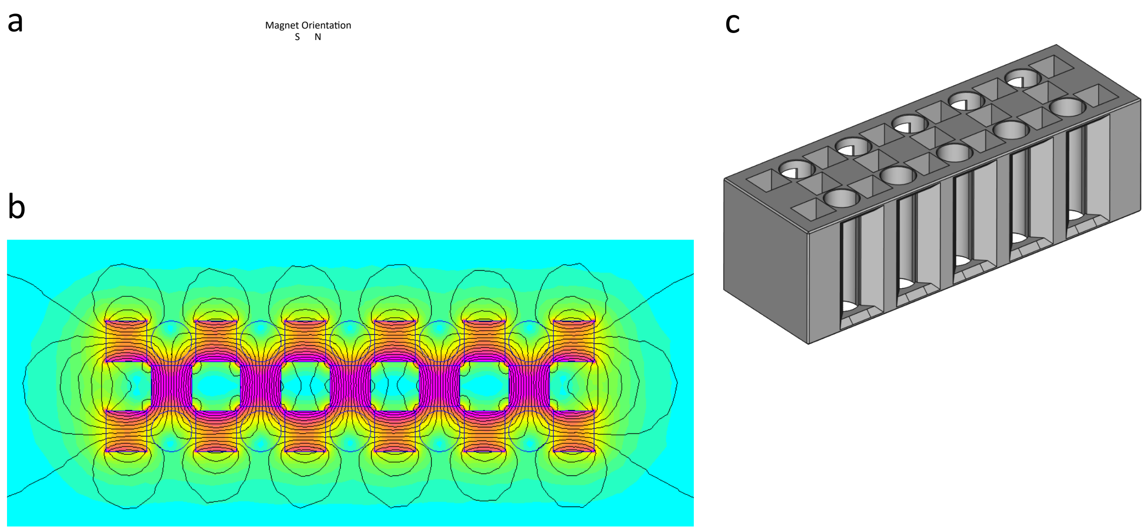

Supplementary Figure 4. Development of the in-house magnetic rack used for $mSiO_2$ NP extraction. a) Schematic of the organisation of the magnet (blue) (Supermagnete, Germany), Material NdFeB, Magnetization N42, 40 × 10 × 10 mm), and position of the tubes (green), dimensions are in millimetre. b) Simulation of the magnetic flux lines and density, realised with FEMM 4.2. This magnet configuration allows the formation of high magnetic gradients that will attract the particles on one side of the tube. c) Three-Dimensional representation of the designed magnetic rack. Gaps of the 3D structure have been included to facilitate the visibility of the samples in the tube during extraction.

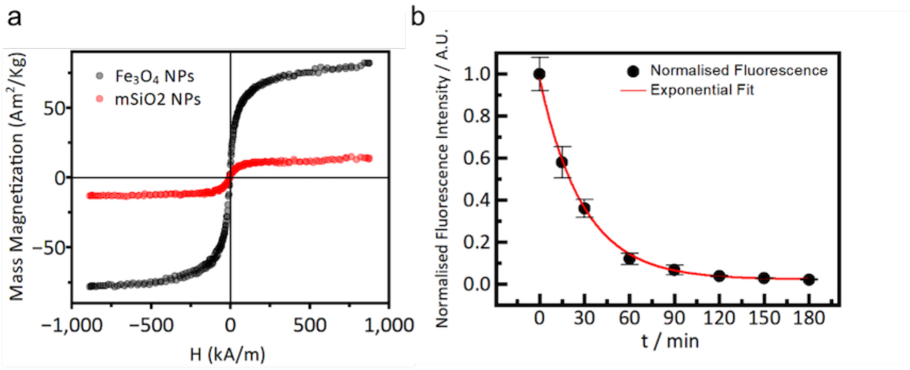

Supplementary Figure 5. Magnetic Properties of $mSiO_2$ NPs. a) Magnetisation curve: the sample presents no coercivity and no remanent magnetization at room temperature indicating that the particles are superparamagnetic. The mass magnetic saturation is lower for $mSiO_2$ compared to the magnetite NPs forming the core because of the presence of the silica shell. Reproduced from (Cursi et al. 2021) with permission from the Royal Society of Chemistry. b) Magnetic pull-down kinetics of particles in the magnetic rack. The particles were diluted to a concentration of 0.5 mg/mL in a 1 mL Eppendorf tube. The progression of the pull down was monitored by measuring the fluorescence of the supernatant exciting at 488 nm and measuring at 515 nm at a Horiba Fluorolog. Fluorescence intensities are reported normalised to the value at t = 0 i.e. before the start of the pull-down experiment. Values are average of 3 independent measurements ± standard error. As it has already been demonstrated (Supplementary Figure 3) that FITC dye does not leak from the silica shell of the MCSP@$fSiO_2$, a decrease in the supernatant fluorescence can be used to assess magnetic precipitation of particles from solution. As shown in the figure, the normalised fluorescence intensity decrease is well-described by an exponential decay curve with the pull down being more than 50% within the first 30 minutes and essentially 100% pull down after t = 120 minutes.

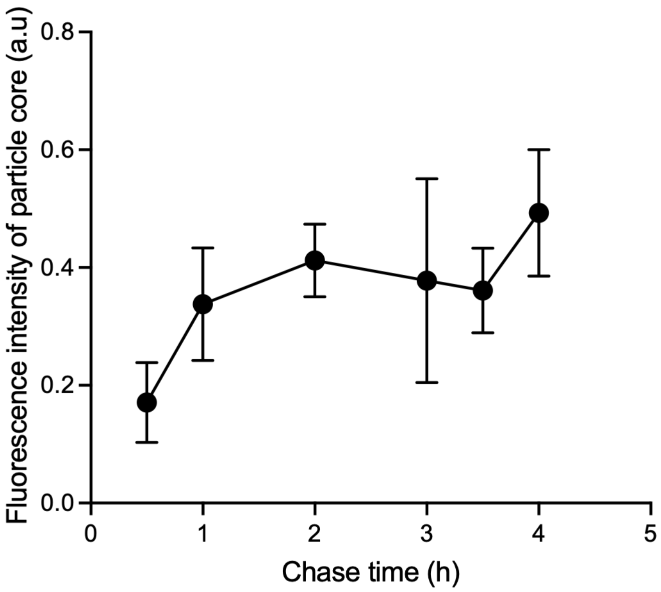

Supplementary Figure 6. Monitoring of particle complex release over time by A549 cells seeded in a 24-well plate. After 10 min pulse with corona nanoparticles (800 µg/mL), adherent A549 cells were washed twice with CMEM media and twice with PBS, and then incubated in complete MEM media for 30, 60, 120, 180, 210 and 240 min. After each chase time, the Cy5 fluorescence intensity of particle cores released in the medium was measured with a plate reader. The error bars represent the standard deviation over 4 replicates.

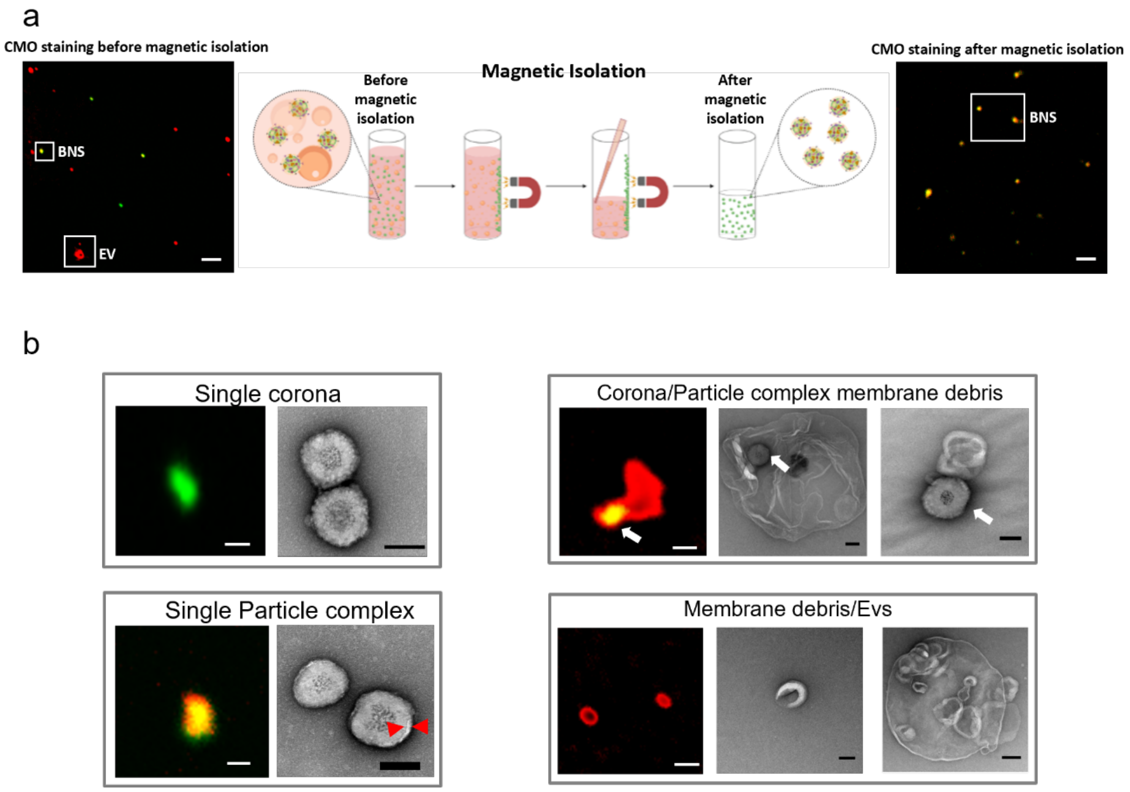

Supplementary Figure 7. Analysis of particle complexes produced using magnetic SiO2 nanoparticles from A549 cells before and after magnetic isolation. (a) Confocal image analysis of the particle complexes (green) and stained with CMO (red) reveal that there are membrane debris/extracellular vesicles (EV) products also collected with the particle complexes during the centrifugation step; these contaminations are stained red, while the particle complexes appear yellow. (b) Confocal and TEM analysis (*Staining n°1*) of the different populations of particles, including particles aggregated with membrane debris, and extracellular vesicles.

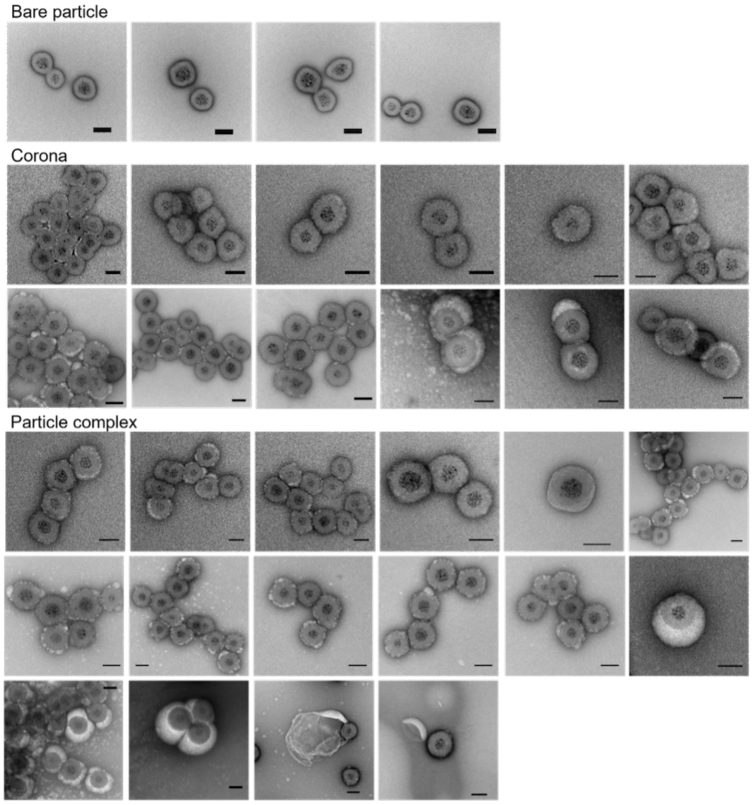

Supplementary Figure 8. TEM analysis of negatively stained (*Staining nº 2 with 1% Trehalose*) $mSiO_2$ NPs, Corona and particle complex derived from A549 cells. For the bare particles, it is possible to observe the electron dense magnetic core and the silica shell but no evident structure is observed at the surface of the nanoparticles. For the nanoparticles with a corona and for the particle complexes, globular structures can be observed at the surface of the nanoparticles. These structures may correspond to the biomolecular corona, however they could be attributed in certain cases (in particular in the case of think staining) to beam damage. It must be noticed the occasional presence of particle complexes aggregated with membranous objects (2 last images). Scale bar: 100 nm

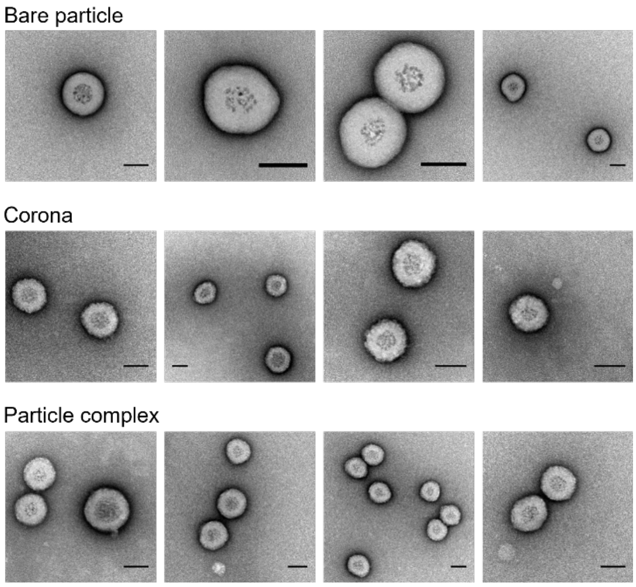

Supplementary Figure 9. TEM of negatively stained (*staining n˚1*) bare $mSiO_2$, Corona and particle complex derived from A549 cells. Trehalose was not used as an additive to the staining agent to prevent artefacts from thick staining and “bubbling”. No structure can be observed at the surface of the bare $mSiO_2$. Globular structures can be observed at the surface of the corona and protein complexes. Scale bar: 100 nm

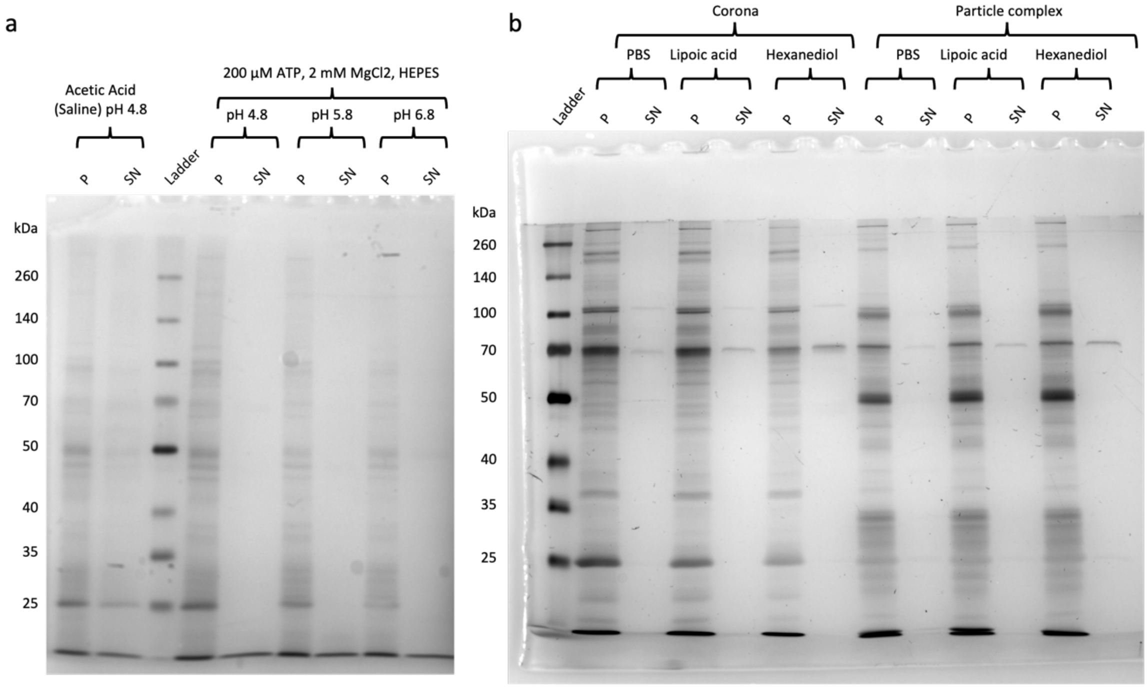

Supplementary Figure 10. Test of detachment of the biomolecular layer. a) In biologically relevant pH conditions. Silver staining of SDS-PAGE of 4 µg of particles after incubation at different pH in presence of ATP and magnesium to allow potential phosphorylation reactions. b) Test of dissolution of the corona and particle complex layer (derived from A549 cells) using Lipoic acid or 1,6-hexanediol, known for dissolving liquid–liquid phase separated biomolecules condensates. Silver staining of SDS-PAGE of 5 µg of particles after 12 h incubation in 1mM Lipoic acid (in PBS), or 5% w/v of 1,6-hexanediol in PBS. The potential proteins released in solution were separated from the nanoparticles by centrifugation and the pellet (P) and supernatant (SN) were loaded separately on the gel after being heated at 95°C for 10 min in presence of loading buffer and DTT. No protein was detected in the supernatant, suggesting that the cell-derived layer didn't detach for the different conditions tested. The visible band in all the supernatants is attributed to a residual contamination from FBS (BSA).

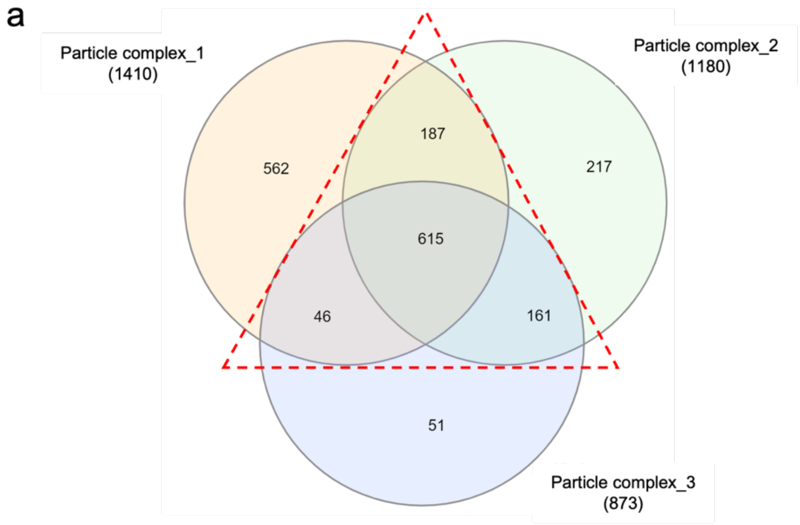

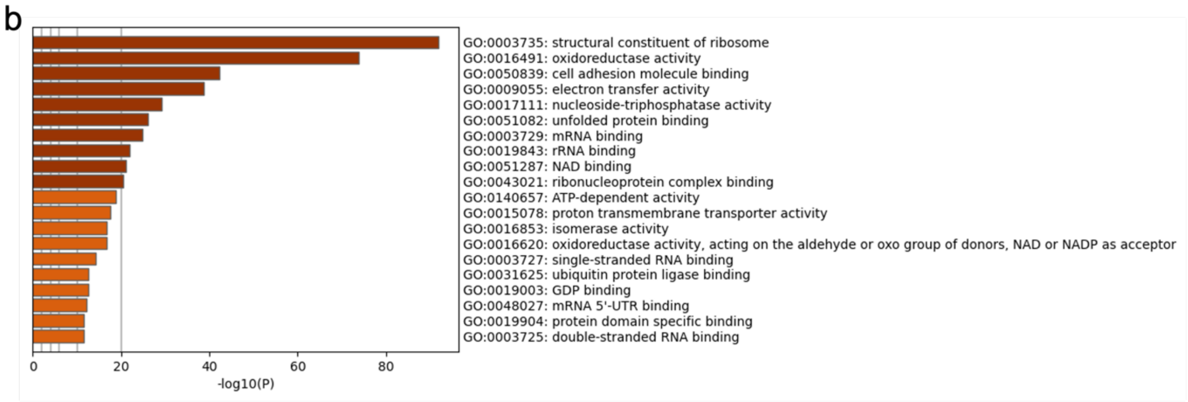

Supplementary Figure 11. Three biological replicates of "heavy" proteins in particle complexes derived from SILAC-A549 cells. a) Venn diagram of "heavy" proteins in the particle complexes. b) GO term analysis of heavy proteins that were identified in at least two biological replicates (within the triangle in a). The GO term (Molecular Function) analysis was performed by Metascape.

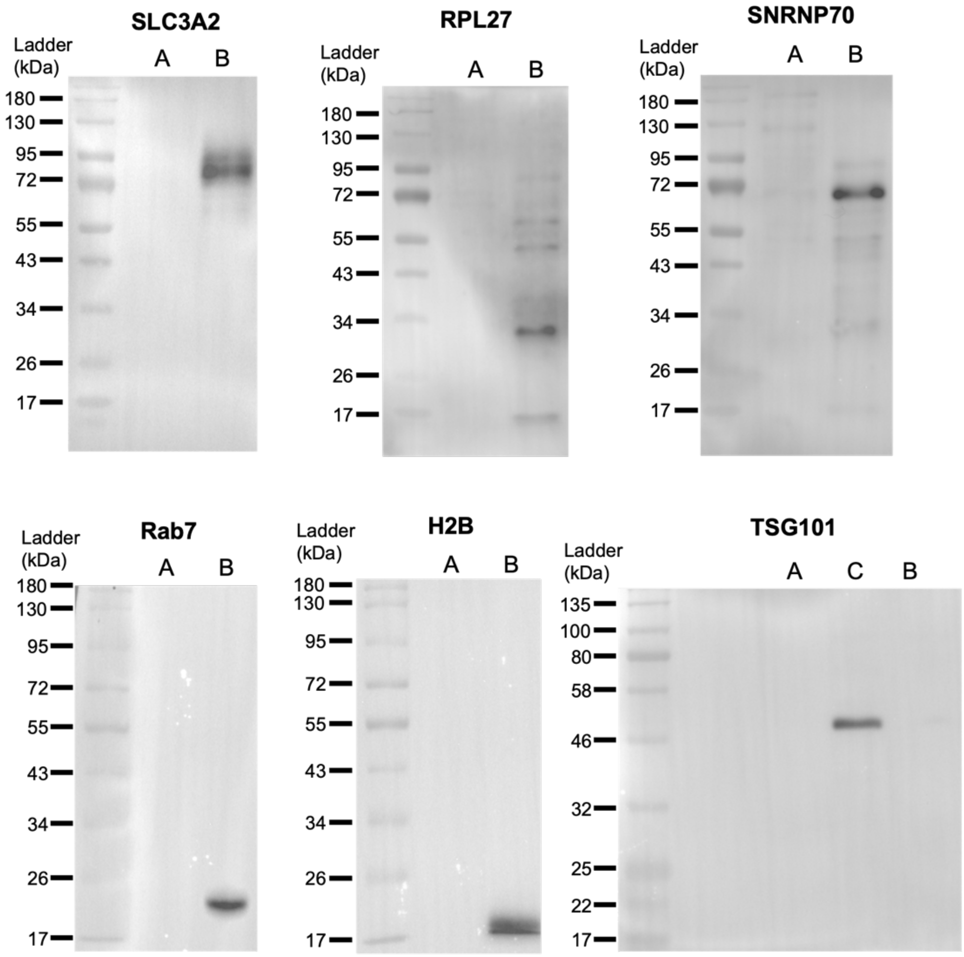

Supplementary Figure 12. Western blot analysis of specific markers in particle complexes and EVs derived from A549 cells. Protein concentrations were determined by microBCA assay, and equal amounts of proteins were loaded in each lane. Sample A is corona, B is particle complex, and C is EVs. The bands correspond to the target proteins detected by the specific antibodies used.

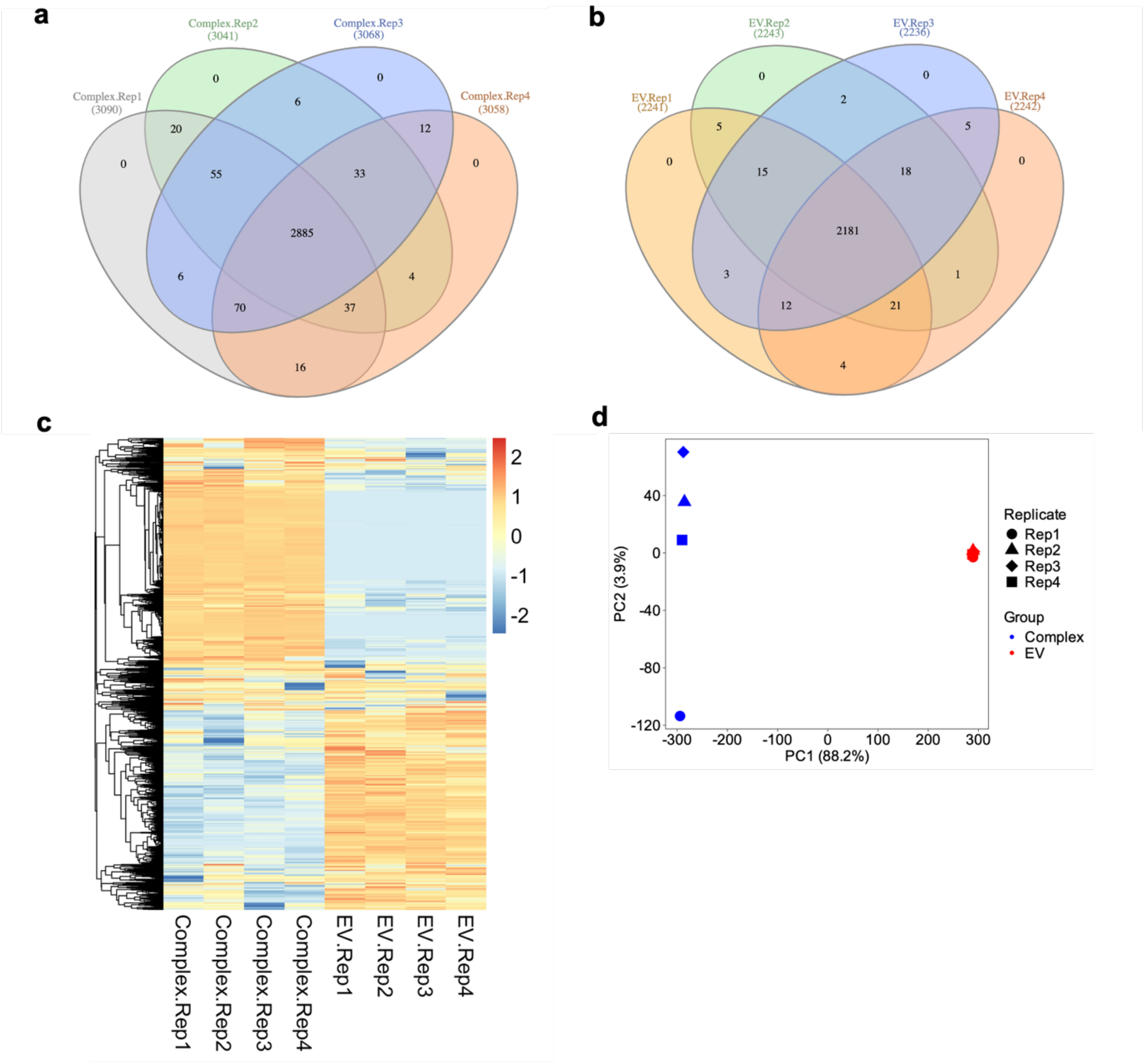

Supplementary Figure 13. Comparison and analysis of proteomes of extracellular vesicles (EVs) and particle complexes derived from SILAC-labelled HEK293 cells. (a, b) Venn diagram showing the overlap of identified 'heavy' proteins in four biological replicates of particle complexes (a) and EVs (b). (c) LFQ intensities were log_2-transformed and then z-score normalised across each protein (row). Columns correspond to individual samples, and rows to proteins included in the analysis. The colour scale represents the resulting row-wise z-scores of log_2 [LFQ intensity], ranging from blue (lowest relative abundance within a protein) to red (highest relative abundance). (d) Principal Component Analysis (PCA) plot illustrating the variance in proteome profiles across different replicates of EVs and particle complexes.

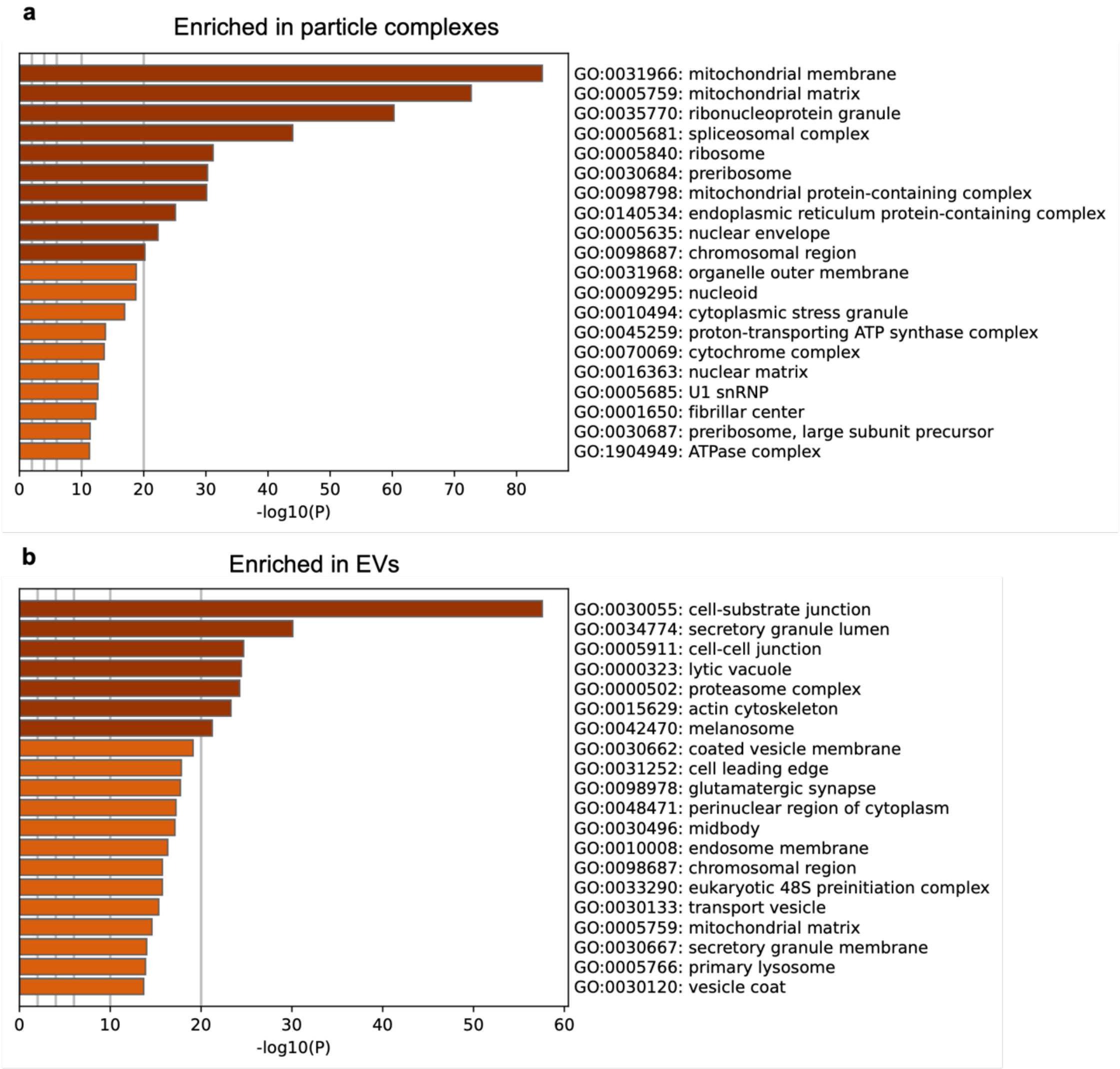

Supplementary Figure 14. Extended enrichment analysis (Go Term - Cellular Component) of differential proteins in extracellular vesicles (EVs) and particle complexes derived from SILAC-labelled HEK293 cells. (a) Bar graph representing the enriched protein complexes in particle complexes (a) or in EVs (b). The GO term analysis was performed by Metascape.

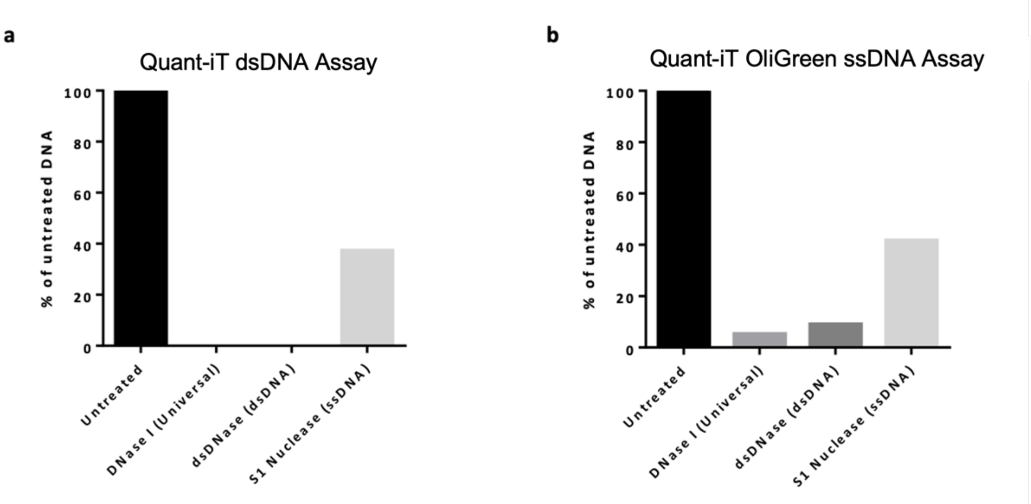

Supplementary Figure 15. Quantification of DNA in particle complexes derived from A549 cells. The DNA was extracted from the isolated particle complexes and treated with different nucleases. The untreated sample was defined as 100%. (a) Quant-iT dsDNA Assay shows the percentage of double-stranded DNA (dsDNA) remaining after treatment with DNase I (Universal), dsDNase, and S1 Nuclease. (b) Quant-iT OliGreen ssDNA Assay shows the percentage of single-stranded DNA (ssDNA) remaining after the same treatments. Please note Quant-iT OliGreen reagent does exhibit fluorescence enhancement when bound to dsDNA.

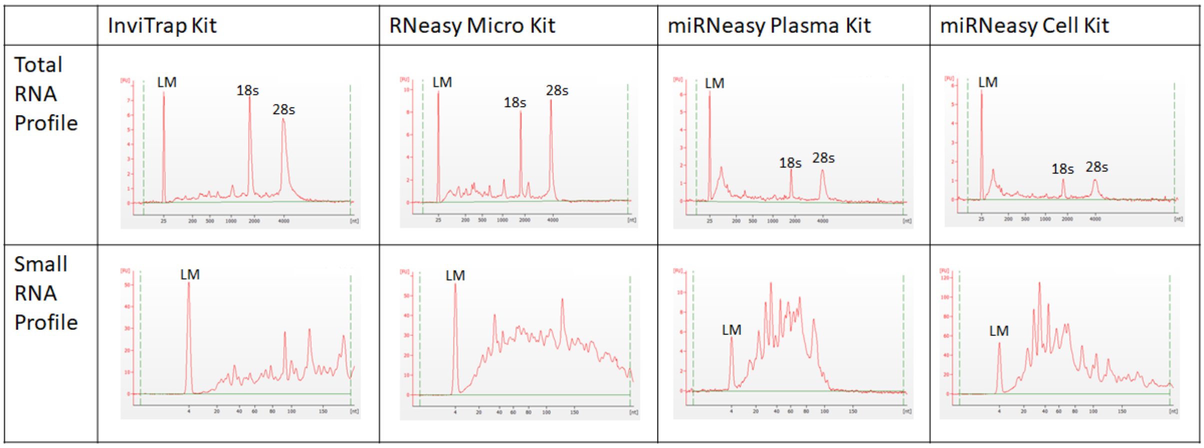

Supplementary Figure 16. RNA profiles for the particle complex derived from A549 cells. The RNA was extracted by using different kits. InviTrap Kit (INVITEK, Cat. NO. 1060100300), RNeasy Micro Kit (Qiagen Cat. NO. 74034), miRNeasy Plasma Kit (Qiagen Cat. NO. 217204), miRNeasy Cell Kit (Qiagen Cat. NO. 217684), LM: ladder marker. Analysis was done by Agilent Bioanalyzer 2100 in total RNA chip and small RNA chip.

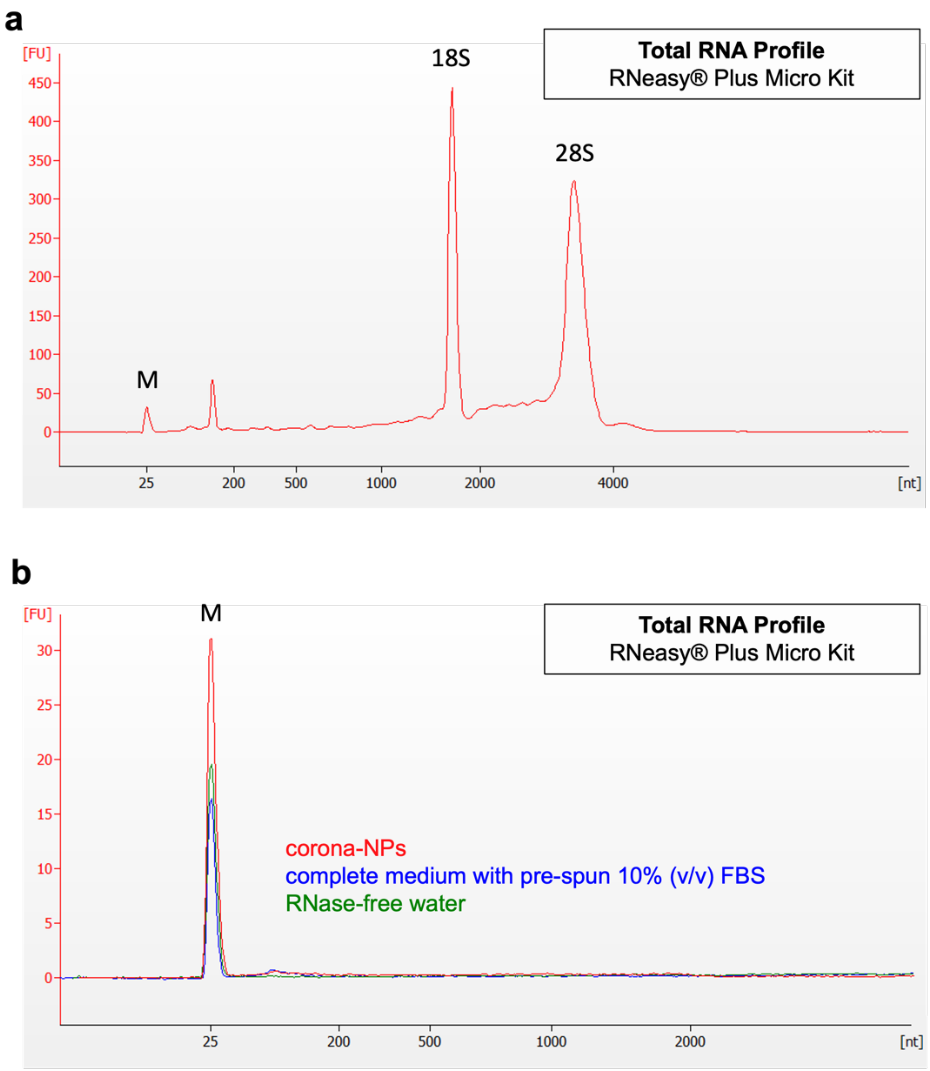

Supplementary Figure 17. Analysis of Total RNA Profiles Using Bioanalyzer. The total RNA was extracted from particle complexes derived from HEK293 cells using the RNeasy® Plus Micro Kit. (a) The RNA profile of particle complexes shows two distinct peaks corresponding to the 18S and 28S ribosomal RNA, as well as long RNAs and small RNAs in lower abundance. (b) The RNA profiles of several control samples, including corona-NPs (red), complete medium with pre-spun 10% (v/v) FBS (blue), and RNase-free water (green). The low signal in the profiles suggests minimal RNA presence. The peak labelled 'M' represents the marker.

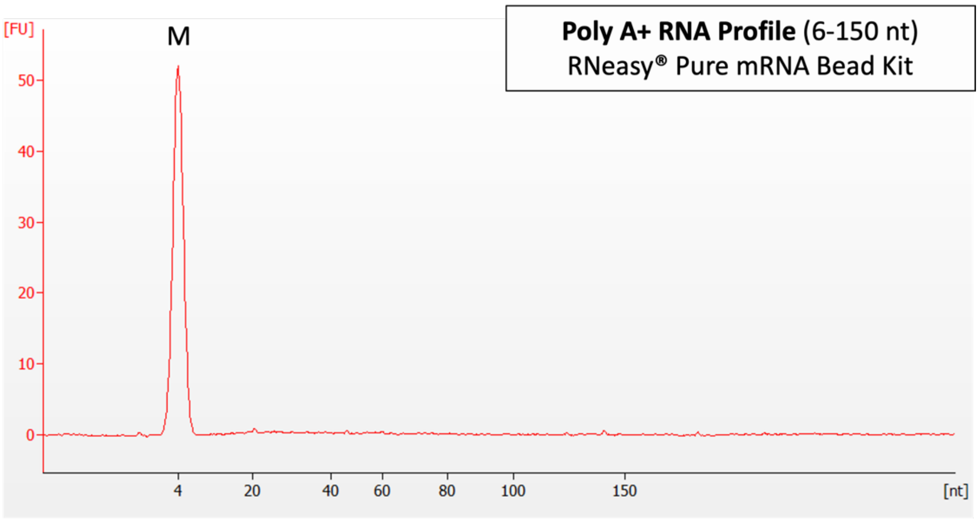

Supplementary Figure 18. Small RNA profile of RNAs purified Using the RNeasy® Pure mRNA Bead Kit. The total RNA extracted from particle complexes derived from HEK293 cells was further purified based on poly A. The purified RNAs were analysed using small RNA chip. The absence of peaks suggests a minimal presence of small RNA.

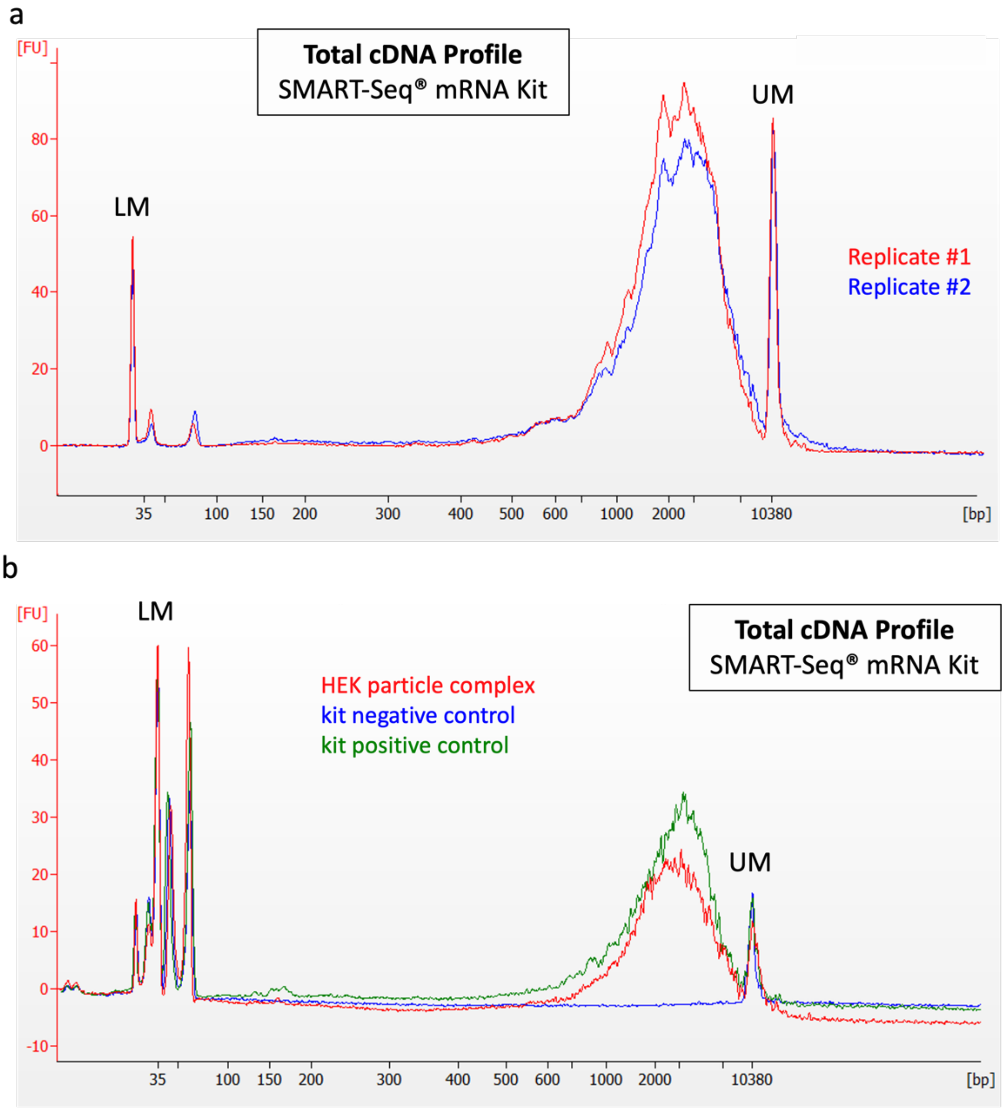

Supplementary Figure 19. Analysis of total cDNA Profiles after reverse transcription. HEK293 cell-derived particle complexes were isolated and the total RNA was extracted. The mRNA was reverse transcribed using SMART-Seq mRNA Kit. (a) The total cDNA profile of particle complexes generated by two independent operators, showing high reproducibility. (b) The total cDNA profiles of several control samples, including particle complex, kit negative and positive controls. LM and UM are the markers.

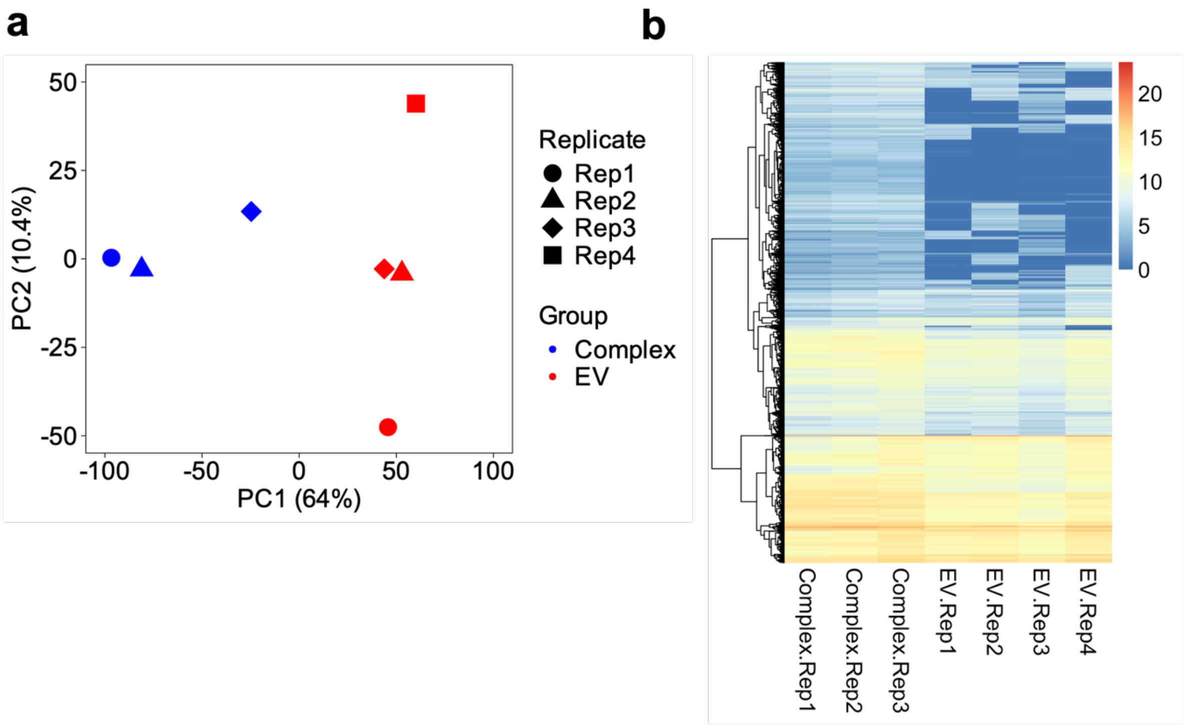

Supplementary Figure 20. Analysis of the RNA-seq of particle complexes and EVs derived from HEK293 cells. (a) Principal Component Analysis (PCA) plot of RNA-seq data showing the separation between two sample groups: Complex (blue) and EV (red). Each shape represents a replicate; three replicates for particle complexes and four replicates for EVs. The distinct clustering indicates differential expression patterns between the Complex and EV groups. (b) Heatmap and hierarchical clustering of the RNA-seq data showing the expression profiles of different samples.

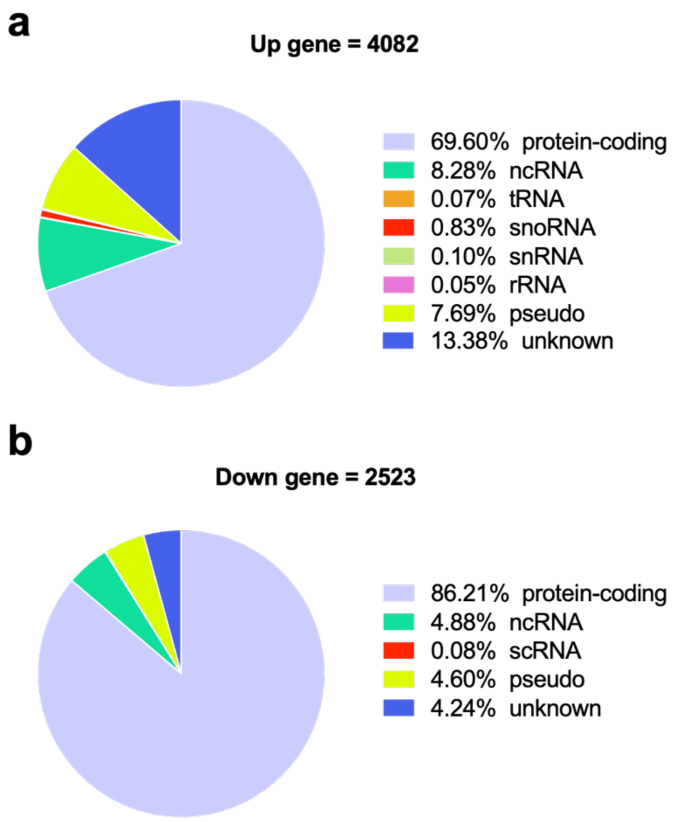

Supplementary Figure 21. Biotyping of the differential RNAs between particle complexes and EVs derived from HEK293 cells.

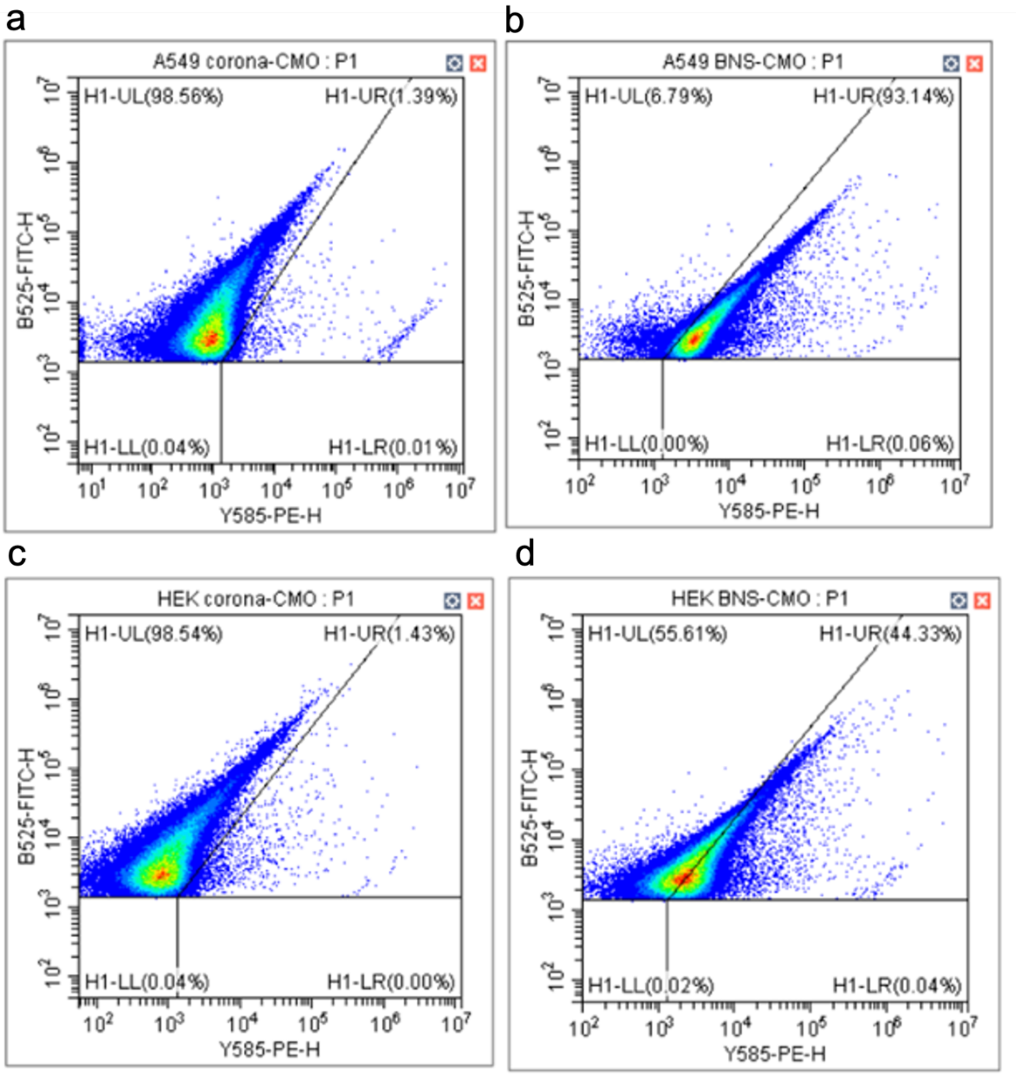

Supplementary Figure 22. Flow Cytometry Analysis of CMO stained particle complexes and corona particles. Serum corona and two types of particle complexes derived from A549 and HEK293 cells were stained with CMO dye to evaluate the lipid abundance. The y-axis (B525-FITC) indicates the core particle fluorescence, and the x-axis (Y585-PE) indicates the CMO staining. (a, c) Two biological repeats of serum corona particles stained with CMO. (b) A549 cell-derived particle complexes stained with CMO. (d) HEK293 cell-derived particle complexes stained with CMO. The quadrant gate was drawn for comparison. The results show particle complexes exhibit higher CMO staining than corona particles, however, CMO staining also differs significantly between the two types of particle complexes, suggesting bilayer lipid may not be universally present.

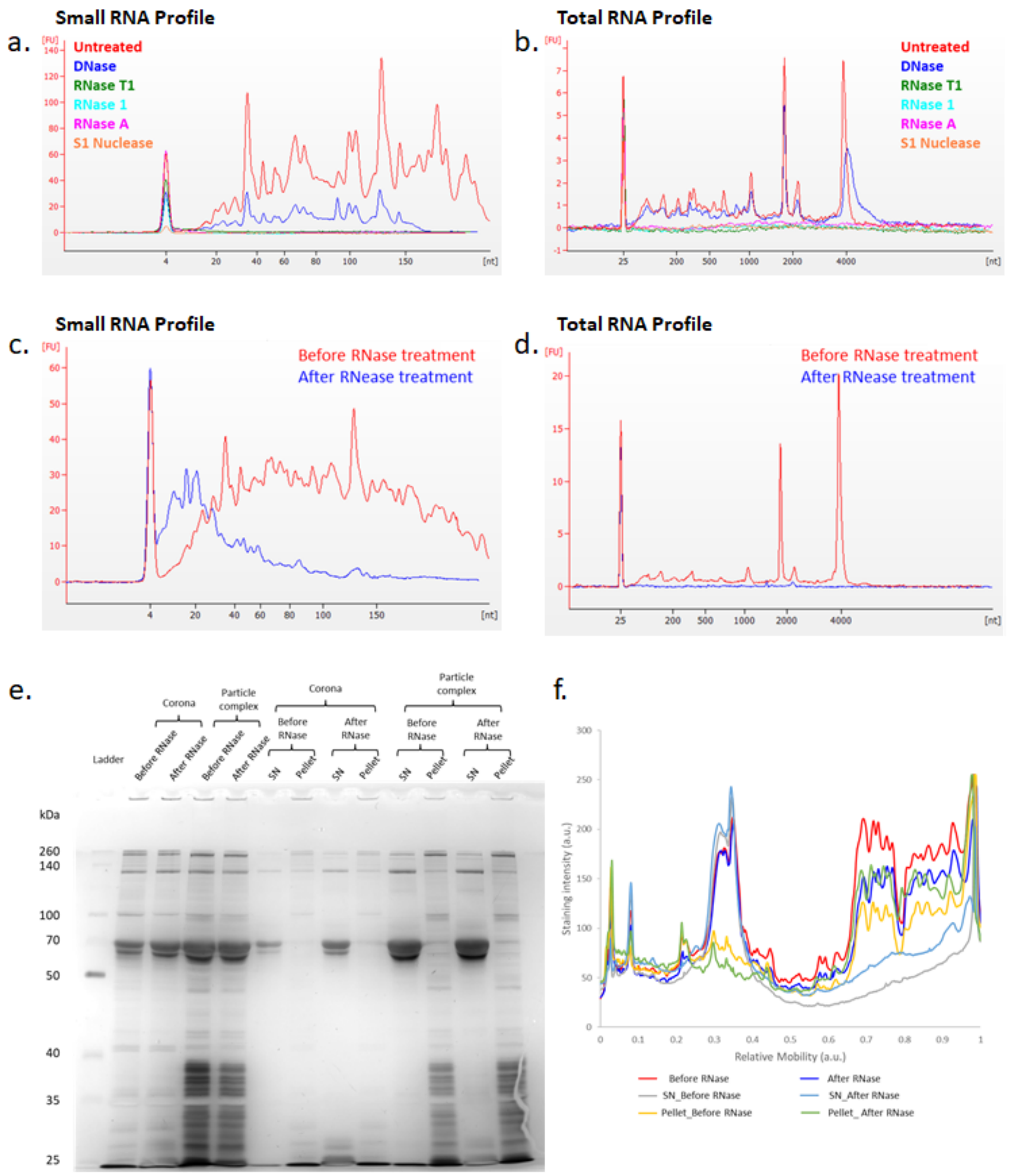

Supplementary Figure 23. Analysis of RNA profiles of particle complexes derived from A549 cells using the Agilent 2100 Bioanalyzer. RNA was extracted from particle complexes and subsequently treated with various nucleases. The small (a) and total (b) RNA profiles were analysed, confirming the purity of extracted RNAs. Intact particle complexes were treated with RNase *in situ,* and the RNA was subsequently extracted and analysed by bioanalyzer. The small (c) and total (d) RNA profiles are completely degraded, suggesting the RNA is accessible on the surface of the biomolecular layer. Following the particle complexes treated *in situ* with RNase, the protein profile was determined by the silver stained SDS-PAGE (e) and the associated densitometry analysis (f), confirming there is no disruption to the protein composition with degradation of the RNA.

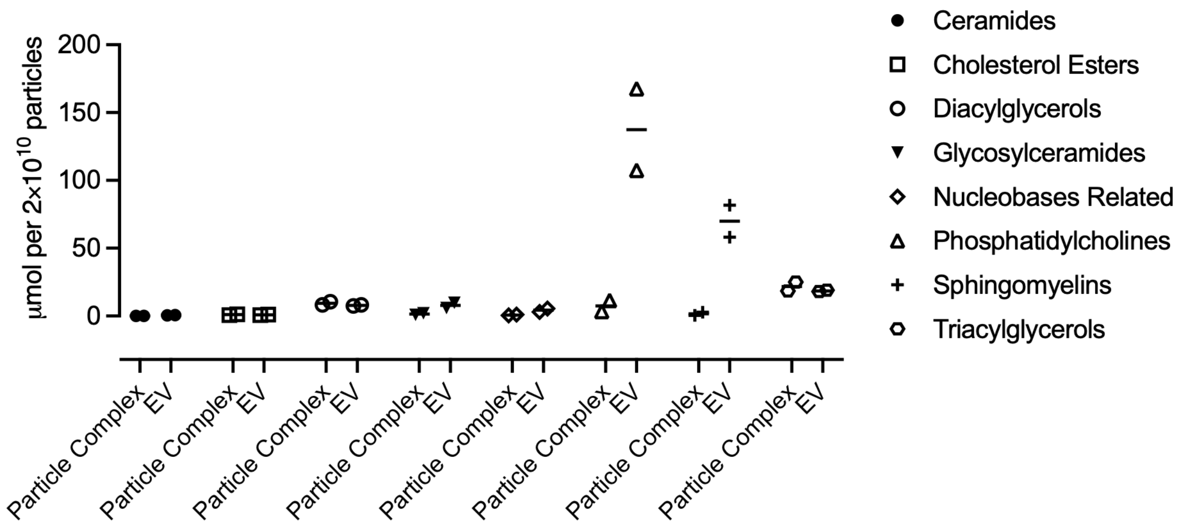

Supplementary Figure 24. Comparative lipid profiles of particle complexes and extracellular vesicles analysed by LC-MS/MS. The particle complexes and extracellular vesicles were derived from HEK293 cells. Lipid concentrations are expressed as micromoles per 2×10^{10} particles. Different symbols represent distinct lipid classes, while short horizontal lines indicate the average values from two biological replicates. Phosphatidylcholines and Sphingomyelins, the two major lipid classes associated with bilayers in extracellular vesicles are essentially absent in particle complexes, while their abundance is as expected for extracellular vesicles. While estimates for population average sizes can be used to extract absolute lipid concentrations, the key fact is that (in distinction to the situation for extracellular vesicles) the ratio of Phosphatidylcholines and Sphingomyelins to all other lipids for particle complexes does not support an enveloping lipid layer.

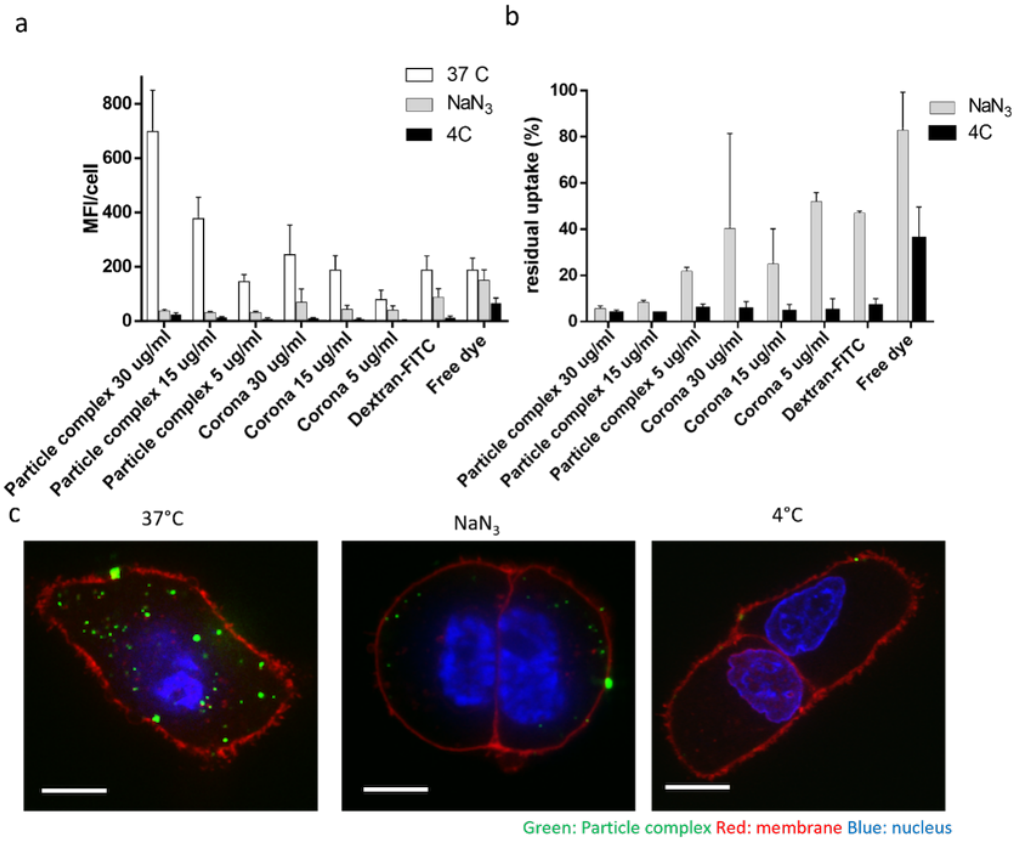

Supplementary Figure 25. The particle complex uptake is receptor mediated and energy dependent. The particle complexes were derived from A549 cells, and the uptake of the particle complexes was carried out in naïve A549 cells. The HCA experiment was conducted on cells depleted in ATP by treatment with sodium azide, and the uptake of particle complex or corona-NPs was compared to the one of cell cultured in regular cMEM (no depletion). The HCA analysis revealed that the uptake is greatly inhibited by the sodium azide treatment (a, b); the results were validated by confocal imaging, showing that only few particles were internalised by the NaN_3 treated cells (c, central image). As a positive control for the inhibition, a set of cells were incubated at 4°C during the particle exposure, and neither the particle complex nor the corona-NPs were internalized, but some particles are stuck on the cell membrane. (n=3, P-value < 0.005). Scale bar: 10 µm.

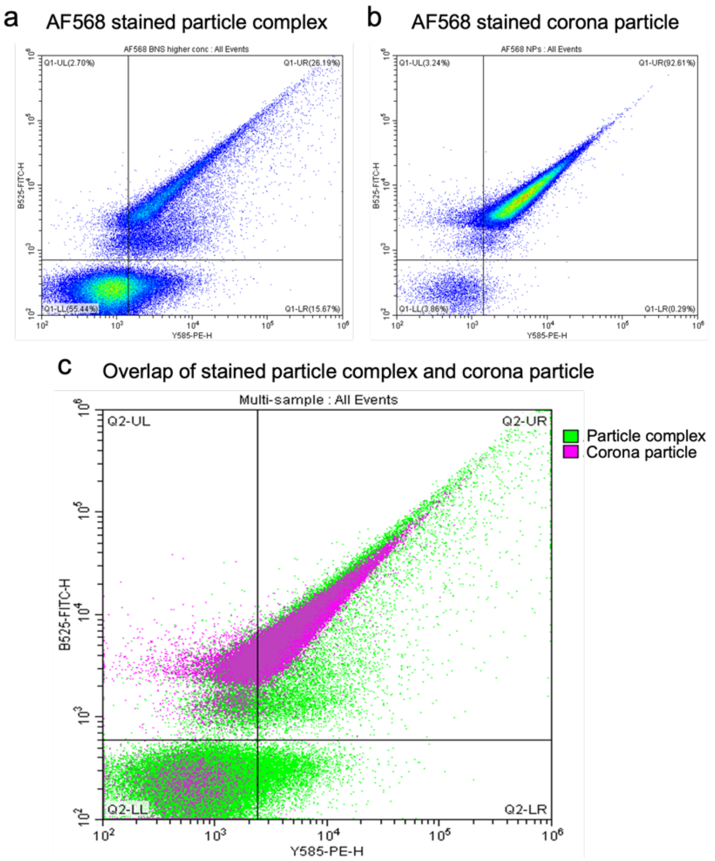

Supplementary Figure 26. Flow cytometry scatter plots depicting the AF568-NHS dye labelling of particle complex derived from A549 cells (a) and corona particle (b). The y-axis (B525-FITC) indicates the core particle fluorescence intensity, and the x-axis (Y585-PE) indicates the AF568 intensity. Events in Q1-UR and Q2-UR are nanoparticles. (c) Overlay of the two samples, indicating both were equally labelled with AF568 dye.

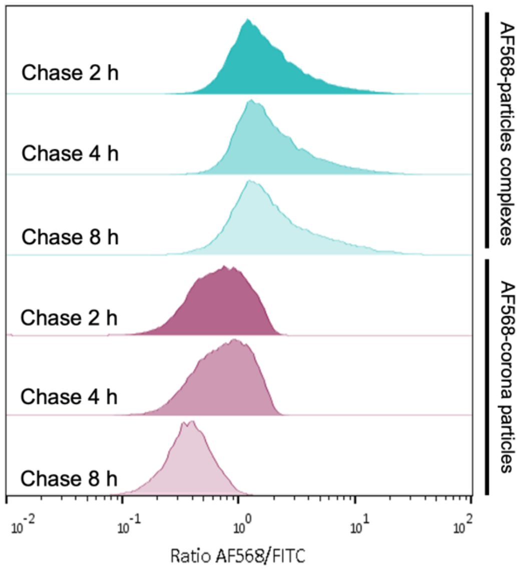

Supplementary Figure 27. Histograms representing the fluorescence intensity changes over the chase time of 8 hours. Particle complexes derived from A549 cells and corona particles (both with FITC core particles) are labelled with AF568. A549 cells were pulsed with either particle complexes or corona particles for 2 h, followed by different chase periods (2, 4, and 8 hours). The x-axis (ratio of AF568/FITC) indicate the ratio between internalized coat proteins and core particles. The top three panels showed little shift as the chase time increases, suggesting that the coat proteins are not degraded in the cells. The bottom three panels showed a noticeable reduction in the ratio distribution at chase 8 h, suggesting significant corona proteins were degraded.

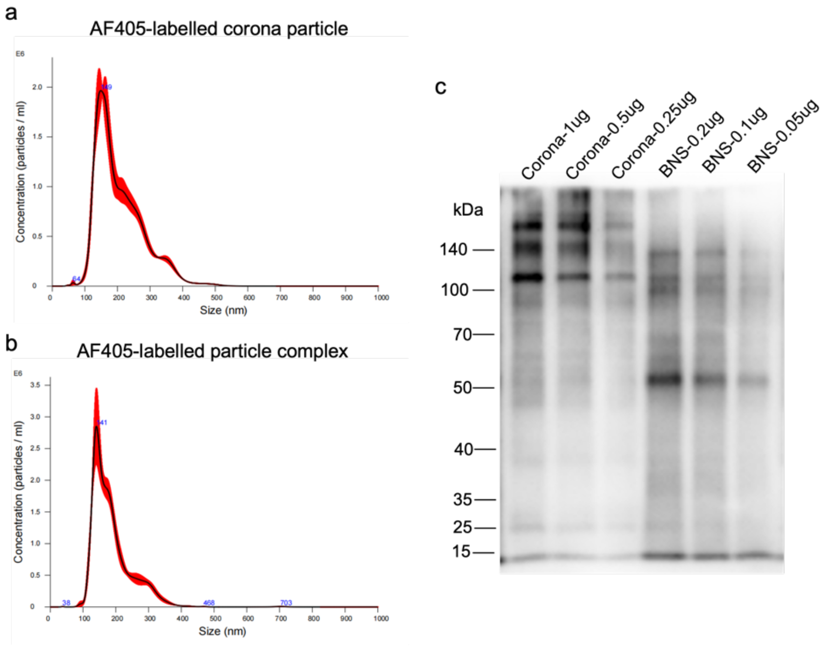

Supplementary Figure 28. Characterization of AF405-labelled corona particles and AF405-labelled particle complexes derived from A549 cells. Nanoparticle tracking analysis (NTA) of the labelled corona particles (a) and particle complexes (b), showing similar primary peak at approximately 140 nm. (c) Western blot using antibodies recognizing AF405 revealing distinct protein profiles between corona particles and particle complexes.

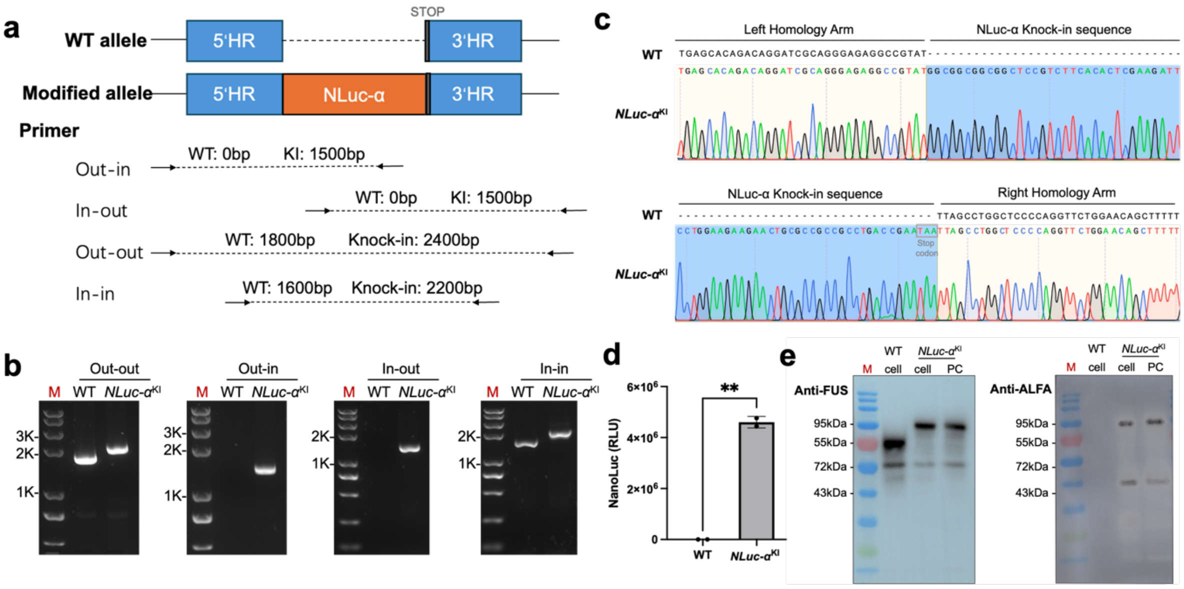

Supplementary Figure 29. Characterisation of FUS-NLuc-α knock-in HEK293 cells and the derived particle complexes. Confirmation of presence of FUS-NanoLuc fusion protein and RNA in the particle complexes derived from mFUS-NanoLuc knock-in cell line. (a) Schematic representation of the FUS knock-in allele. (b) DNA gel electrophoresis of PCR products, confirming the integration of the knock-in cassette. Several pairs of primers were designed and indicated in the scheme. (c) Sequencing results of the PCR product, confirming the correct insertion of NLuc-α to FUS allele. (d) Quantification of bioluminescence activity using the lysates from wildtype and knock-in cell lines, confirming function of fusion protein FUS-NLuc-α. Two-tailed t-test, ** $p<0.01$. (e) Western blots of proteins extracted from wildtype cells, knock-in cells, and the knock-in cell derived particle complexes.

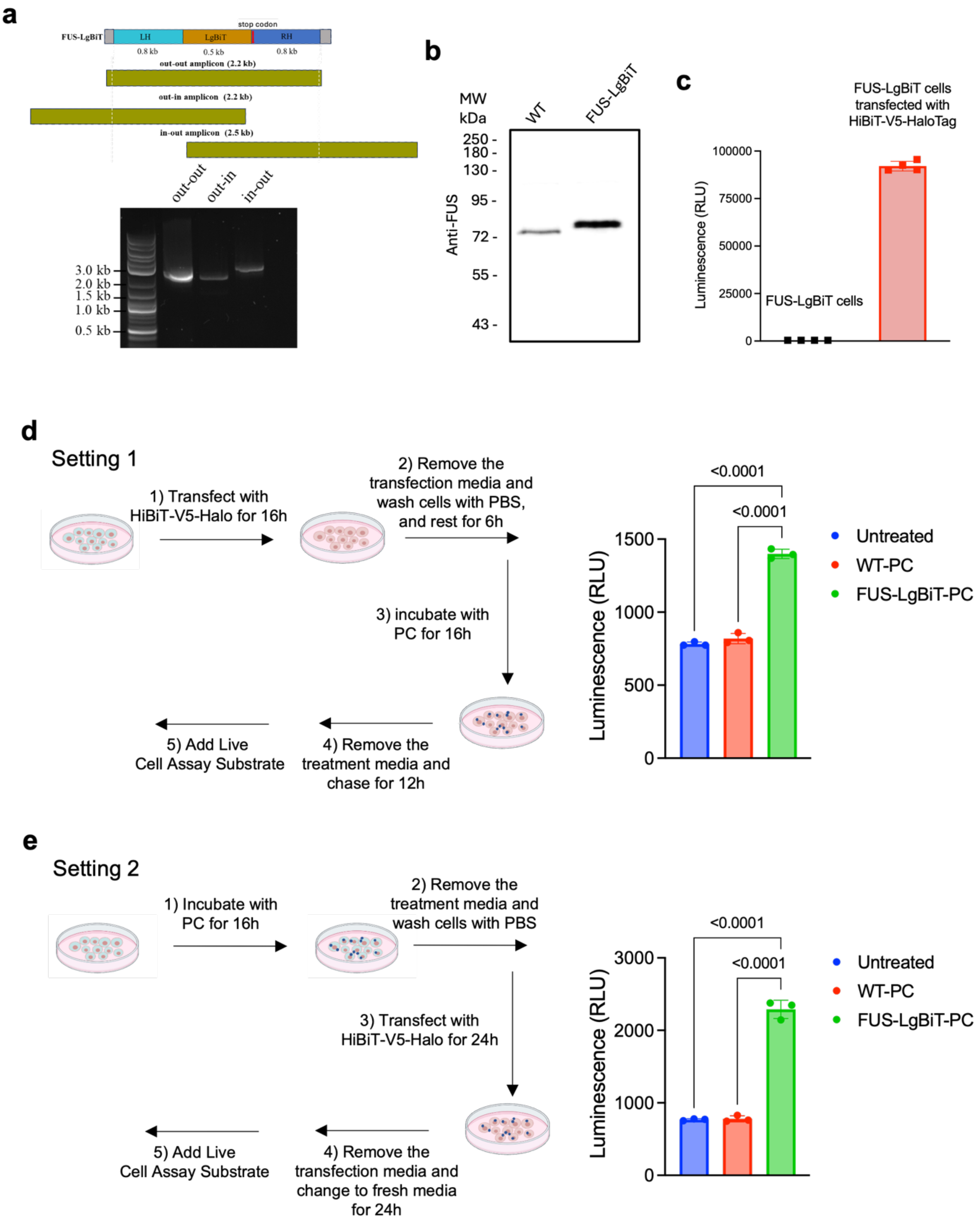

Supplementary Figure 30. Validation of cytoplasmic delivery of FUS protein via split nanoluciferase assay. (a) The LgBiT component of nanoluciferase was knocked into the FUS locus in HEK293 cells using CRISPR-Cas9. PCR was performed to validate the insertion, and the resulting amplicon was sequenced using Sanger sequencing to confirm the knock-in cassette. (b) Western blot analysis

confirmed the successful insertion of LgBiT at the FUS locus. The knock-in cells exhibit a larger apparent molecular weight than the wild-type FUS when anti-FUS antibody was used. (c) Bioluminescence measurement of FUS-LgBiT knock-in cells transfected with HiBiT-V5-HaloTag plasmid, demonstrating robust nanoluciferase reconstitution. (d-e) Schematic workflows and bioluminescence quantification for two experimental settings. (d) Setting 1: Cells were first transfected with HiBiT-V5-HaloTag for 16 hours. The transfection reagents were removed, and the cells were washed with PBS for three time and rested for 6 hours. Cells were then incubated with particle complex (PC) for 16 hours, and chase for 12 hours. (e) Setting 2: Cells were first incubated with particle complexes for 16 hours, followed by HiBiT-V5-HaloTag transfection for 24 hours, and chase for 24 hours. In both settings, FUS-LgBiT-derived particle complexes (FUS-LgBiT-PC) led to significantly higher bioluminescence signals compared to wild-type particle complexes (WT-PC) and untreated controls, which is comparable to an estimated bioluminescene signals per well (around 800-2000) when the FUS-LgBiT protein on particle complexes is 100% delivered to cytosol. The molecular interaction of the two complementary subunits of nanoluciferase confirms the cytosolic delivery of particle complex proteins. Statistical significance was determined using one-way ANOVA ($p < 0.0001$).

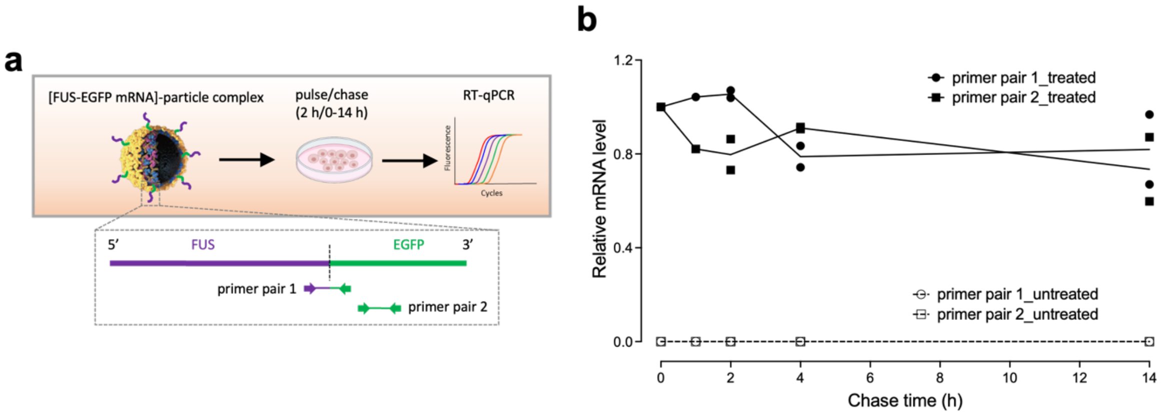

Supplementary Figure 31. Experimental workflow for analysing [FUS-EGFP mRNA]-particle complex using pulse/chase labelling and RT-qPCR. (a) Schematic representation of the experimental design. FUS-EGFP mRNAs were loaded to particle complexes derived from FUS-EGFP stable HEK293 cells confirmed by RT-qPCR. These particle complexes are incubated with naïve HEK293 cells for 2 h and followed by various chase periods. Two pairs of PCR primers are designed to evaluate the kinetics of FUS-EGFP mRNA in recipient cells. (b) The levels of FUS-EGFP mRNA evaluated by RT-qPCR over various chase periods, indicating the mRNA is retained.

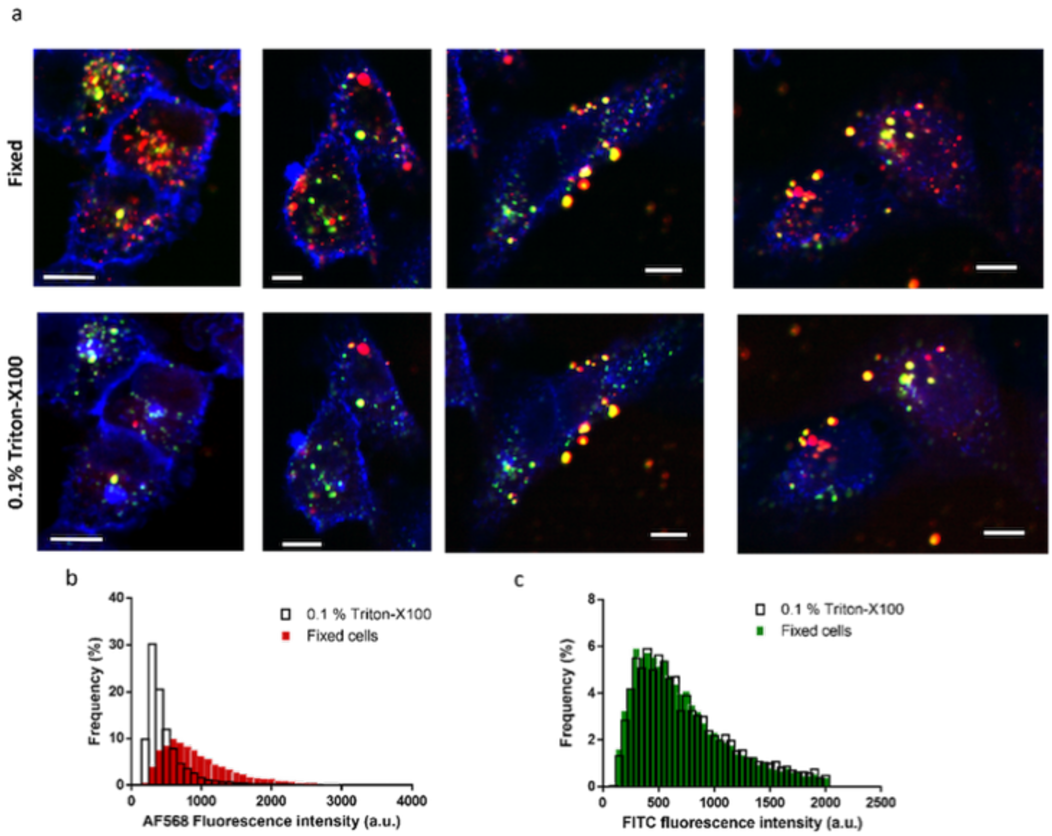

Supplementary Figure 32. The particle complex biomolecular cargo is dissolved by detergents. HCA images (a) and analysis (b, c) showing A549 cells treated with 100 µg/ml AF568-particle complex derived from A549 cells after fixation and the comparison with the same cells after subsequent permeabilization. The standard permeabilization (0.1% Triton-X100, 10 mins) dissolved the vast majority of particle complex cargoes after detachment (red, fluorescence intensity quantification in b), and also partially affected the particle complex complex (appearing yellow). The fluorescence signal of the particle complex core (green, fluorescence intensity quantification in c) is not affected by the treatment. Blue: cell membrane, green: particle complex core, red: particle complex cargo. Scale bar: 10 µm.

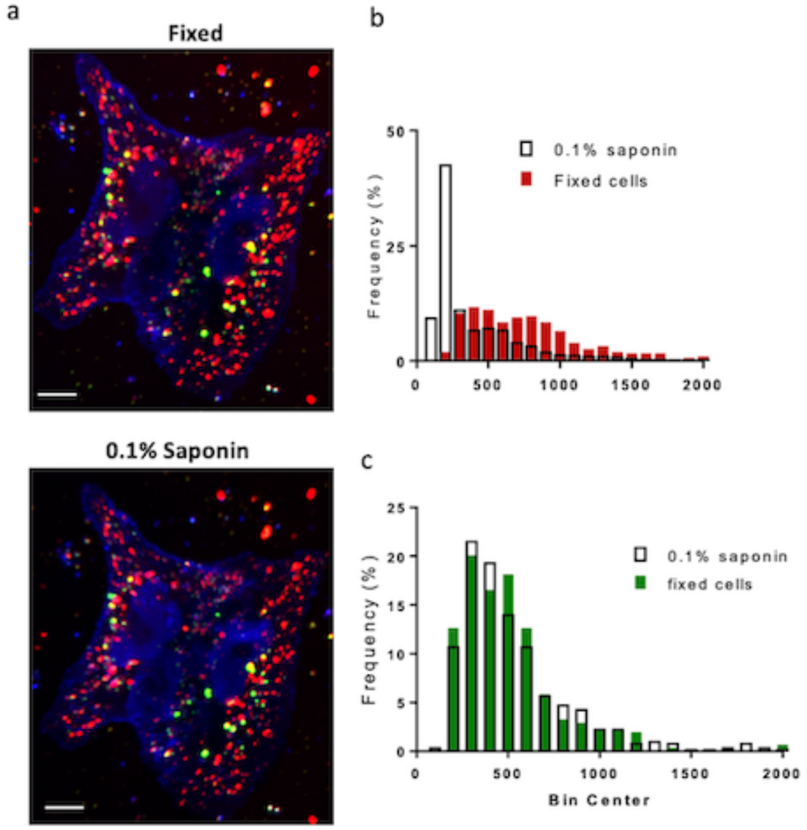

Supplementary Figure 33. The particle complex biomolecular layer is preserved after permeabilization with saponin. HCA images (a) and single cell analysis (b) of cells treated with 100 µg/ml AF568-particle complex derived from A549 cells after fixation (red bars) or after saponin permeabilization (white bars). The mild permeabilization had a lighter impact on the particle complex cargoes loss. Blue: cell membrane, green: particle complex core, red: particle complex cargo. Scale bar: 10 µm.

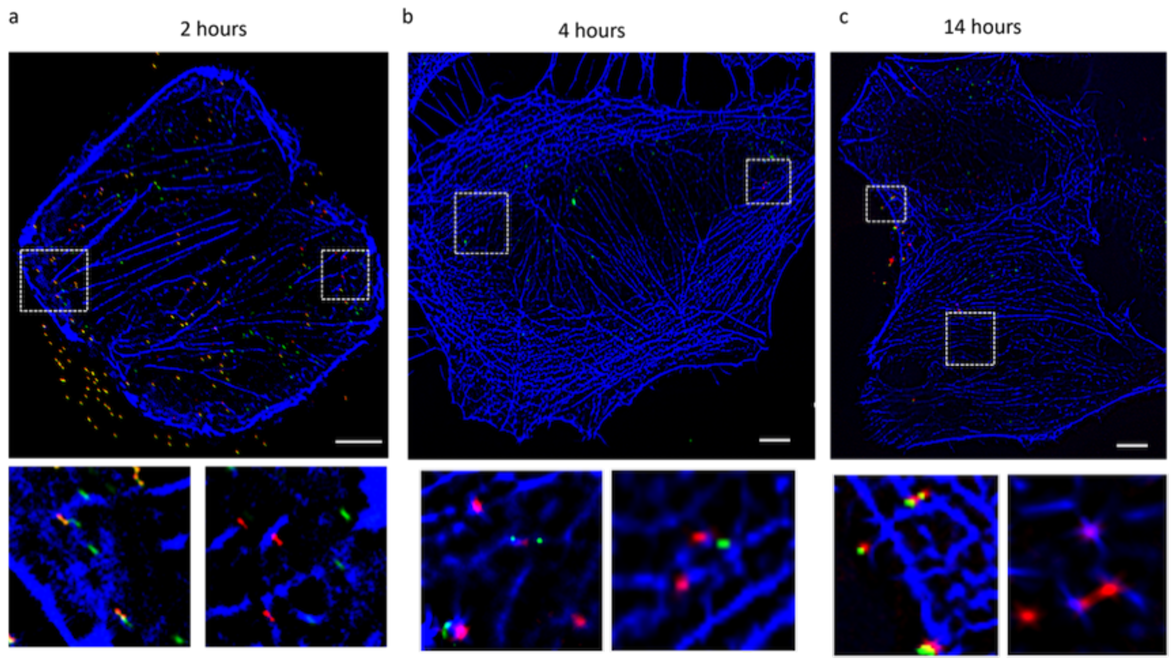

Supplementary Figure 34. Co-localisation analysis of particle complex and actin. A549 were treated with 40 ug/ml of AF-568 particle complex derived from A549 cells for 2 hours and then chased for different times. The cells were thein stain with Phalloidin-TexasRed and imaged in super resolution mode (SRRF). The co-localisation degree of the particle complexes and the cargo alone is significant within the first 4 chase hours. At late timepoint, 14 hours chase, a limited number of cargoes were observed while interacting with the actin. Blue: actin, green: particle complex core, red: particle complex cargo. Scale bar: 10 um.

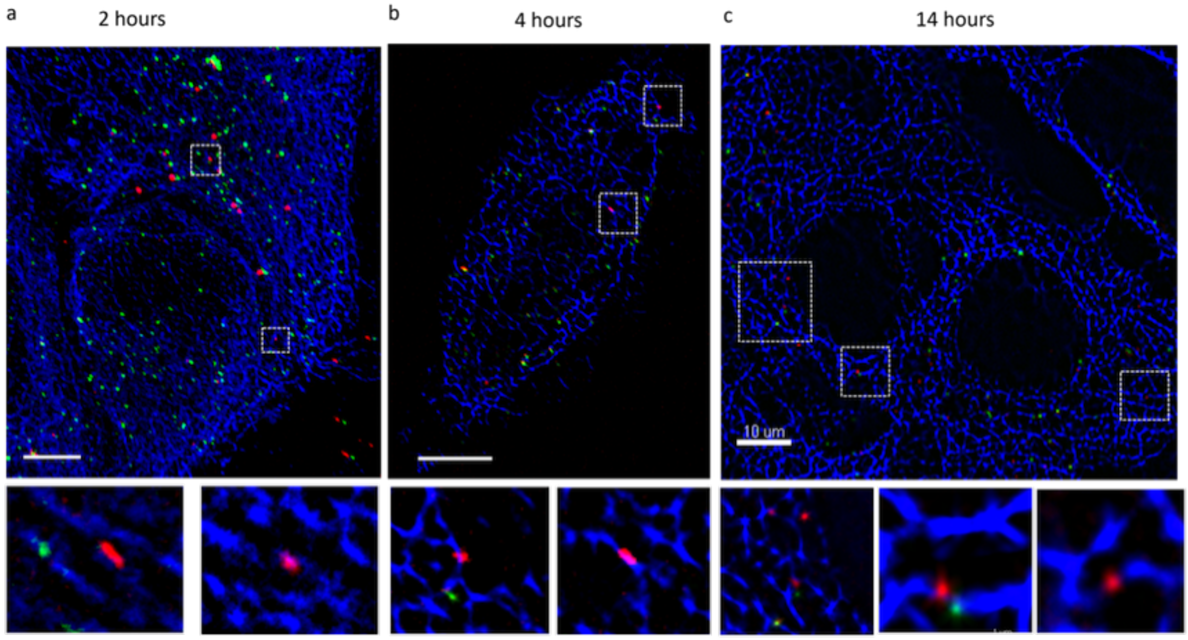

Supplementary Figure 35. Co-localisation analysis of particle complex and tubulin. A549 were treated with 40 ug/ml of AF-568 particle complex derived from A549 cells for 2 hours and then chased for different times (2, 4, 14 hours). The cells were subsequently stained with an anti-tubulin antibody and imaged in super resolution (SRRF). The co-localisation degree of the cargo was constant for the first timepoints (2 and 4), as for the core particles. At late timepoint the co-localisation degree increased. Blue: tubulin (immunostaining), green: particle complex core, red: particle complex cargo. Scale bar: 10 um

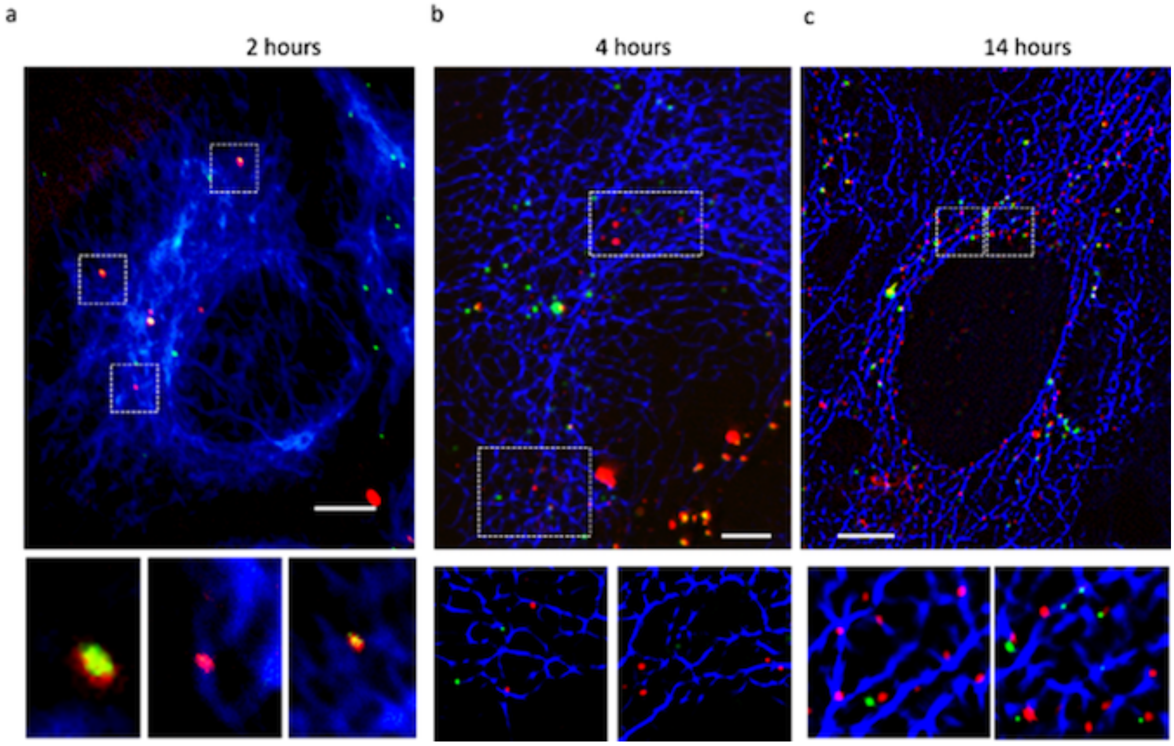

Supplementary Figure 36. Co-localisation analysis of particle complex and vimentin. A549 were treated with 40 µg/ml of AF-548 particle complex derived from A549 cells for 2 hours and then chased for different times (2, 4, 14 hours). a) after 2 hours chase most of the particle complex complexes did not appear co-localised with the intermediate filaments (IF) if not sporadically; b) after 4 hours, most of the complexes are split into cargoes and cores, both partially co-localised. C) After 14 hours chase the localisation of the particle complex cargoes appeared to be increased if compared to the previous timepoints. Blue: vimentin (immunostaining), green: particle complex core, red: particle complex cargo. Scale bar: 10 µm.

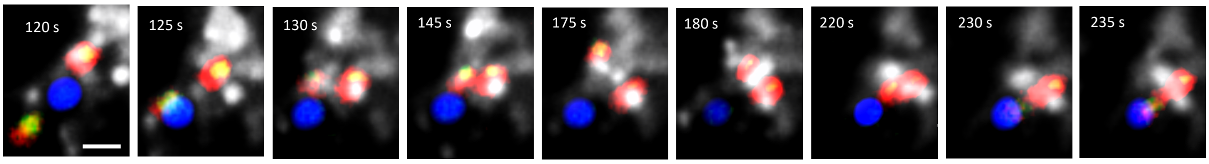

Supplementary Figure 37. Live cell imaging of interactions between particle complex, mitochondria, and P body. A549 cell derived particle complexes were labelled with AF568 and treated HEK293 cells. Blue, EGFP-DDX6; Grey, mitotracker; Red, coat; Green: core particle. Scale bar, 2 μm. The complex coat undergoes multi-centred contacts: exchange between coat (t=145s), contacts with mitochondria (t=180s-220s), and deposition to P body (t=230s-235s).

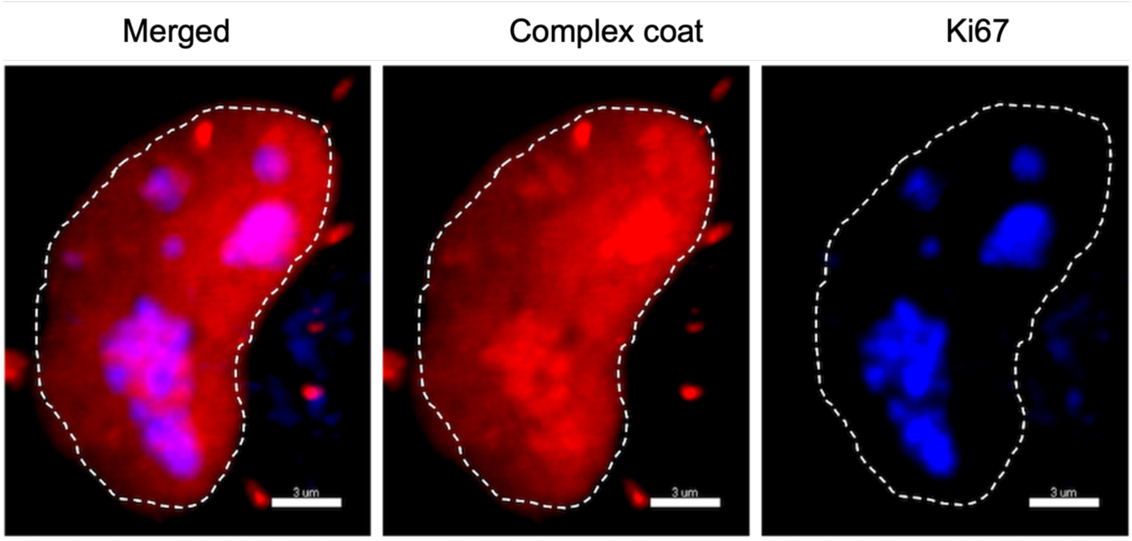

Supplementary Figure 38. The nuclear delivery pattern. A549 cell-derived particle complexes were labelled with AF568 and treated with naïve A549 cells. In part of the cells the nuclear distribution of coats (red) appeared to be associated with nucleolar and Cajal body (blue), however, not all coats exhibit the co-localisation. Red, coat; Blue: Ki67 (immunostaining). Scale bar: 3 µm.

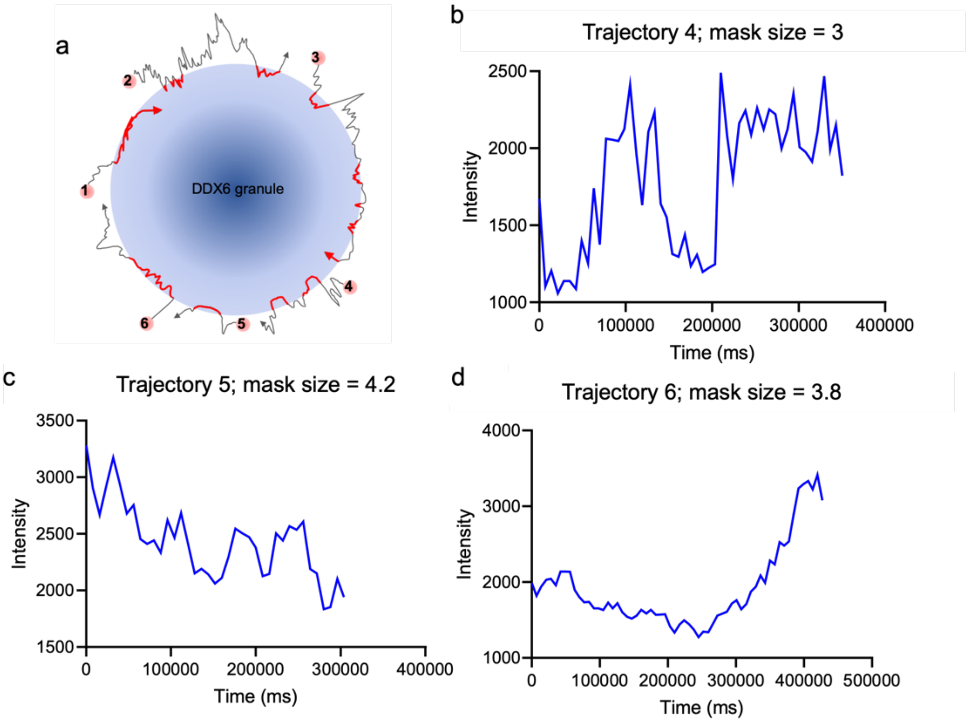

Supplementary Figure 39. 3D mean fluorescence intensity of EGFP-DDX6 over the time course. The coat proteins on particle complexes derived from A549 cells were labelled with AF405. HEK293 cells were transfected with EGFP-DDX6 to label P bodies. After the treatment with the particle complexes, the interaction between particle complex coat and DDX6 was visualized by confocal microscopy. The masks were include the entire granules. The mean fluorescence intensity of EGFP-DDX6 was shown to increase or decrease after multiple contacts with particle complex coat, suggesting that molecular exchange between the coat and DDX6 RNA granule.

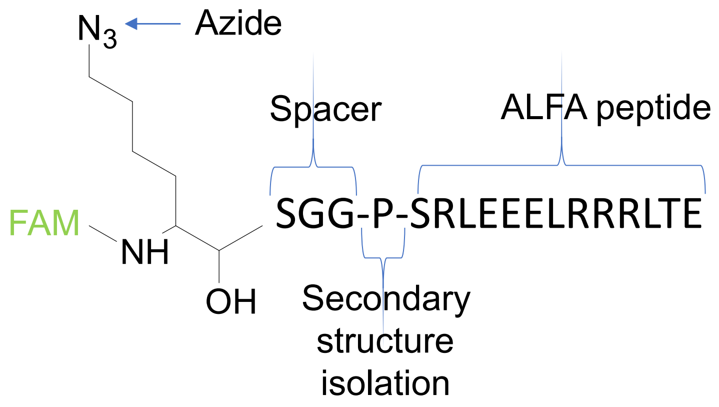

Supplementary Figure 40. Design of the functionalising peptide containing the ALFA sequence, the fluorophore FAM and the azide group for the conjugation to the particle complex surface.

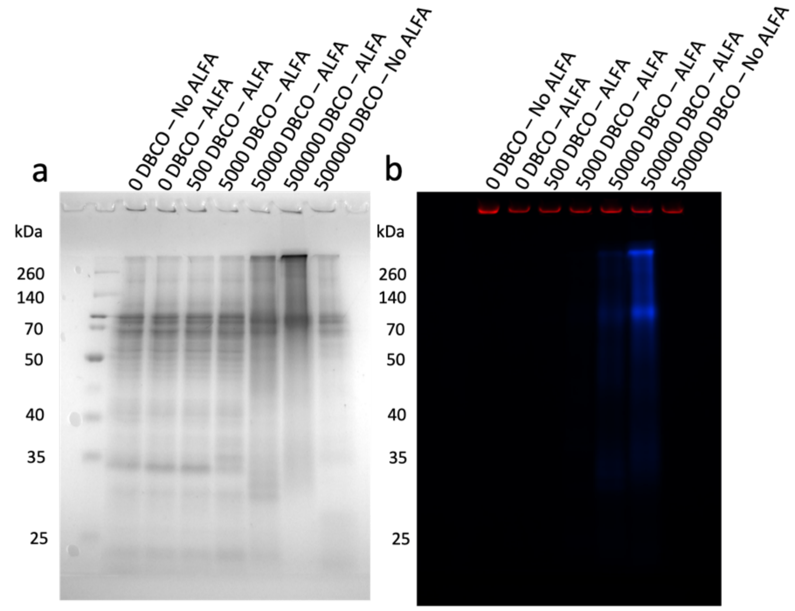

Supplementary Figure 41. SDS-PAGE of corona nanoparticles after their labelling with the fluorescent ALFA peptide using different amount of DBCO-NHS (ratio DBCO/NP is indicated). (a) Coomassie staining revealing the proteins, (b) Florescence imaging. Red: Cy5-NPs, Blue: FAM-ALFA peptide. The colocalization of the proteins and the fluorescent signal from the peptide in for the sample with large amount of DBCO-NHS in denaturing condition indicates the covalent bond between the peptide and the peptide.

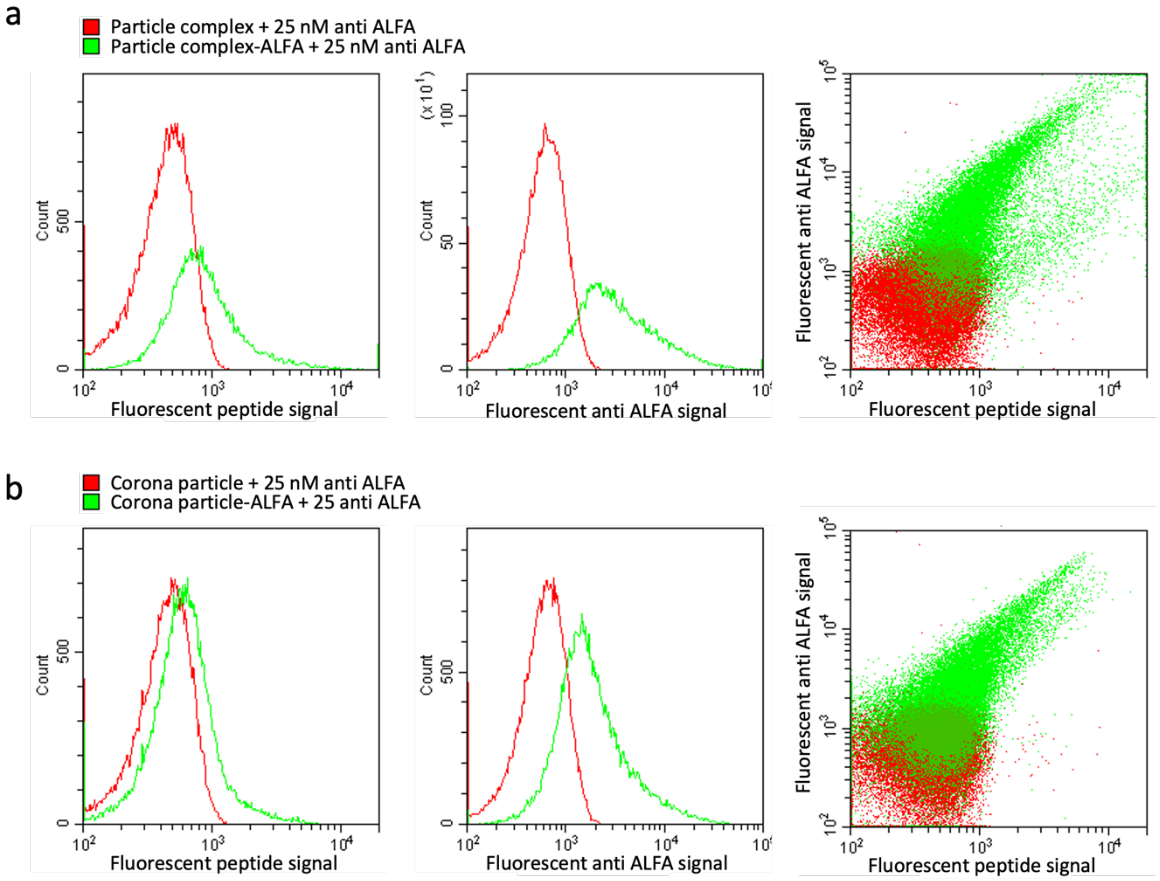

Supplementary Figure 42. Flow cytometry of a) particle complex derived from A549 cells and b) corona particle functionalised or not with ALFA peptide after incubation with 25 nM anti ALFA single domain antibody (anti ALFA) labelled with a Cyanine 3. The shift toward the higher fluorescence intensity of the peptide signal for the functionalised particles compared to the initial particles indicates the binding of the peptide to the particles. The higher fluorescence intensity of the anti ALFA signal for the functionalised particles indicates the accessibility and functionality of the ALFA peptide at their surface. The correlation of the two signals strengthen the conclusion that the signals shits are respectively due to the presence of the peptide and its recognition by the anti ALFA.

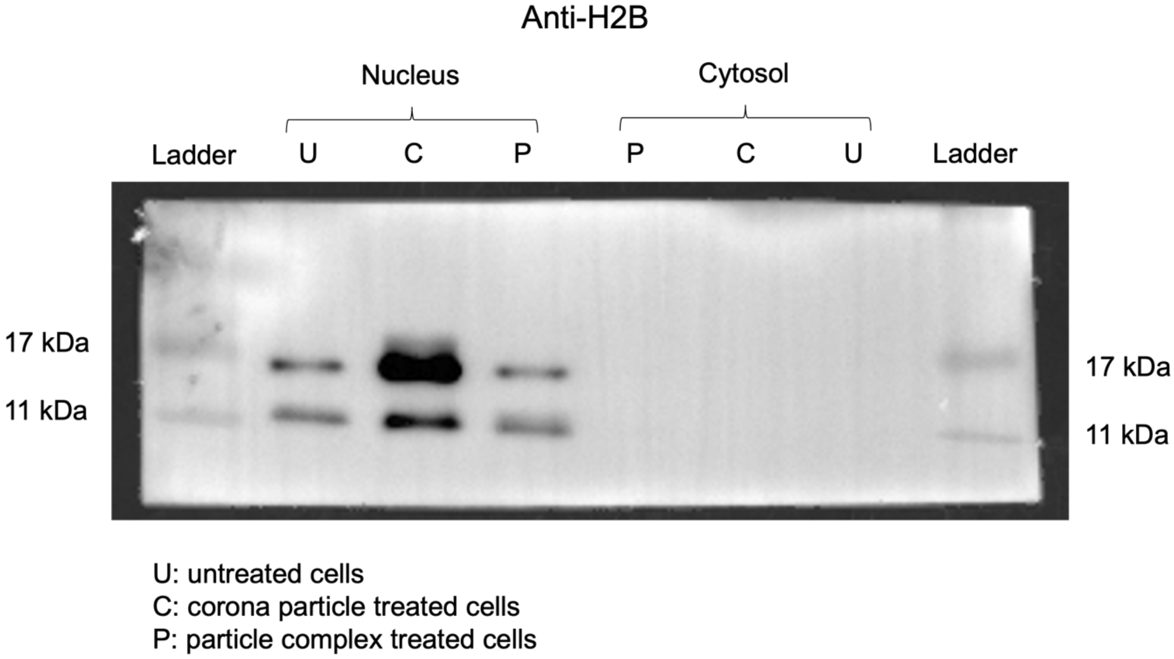

Supplementary Figure 43. Western blot of nuclear and cytoplasmic fractions using anti-H2B antibodies. The cells were fractionated to cytoplamic and nuclear fractions. 3.2 μg of total proteins were loaded to each lane. The blot shows negligible amount of H2B protein were detected in the cytosol fraction, suggesting little contamination from the nucleus to cytosol during the fractionation procedure.

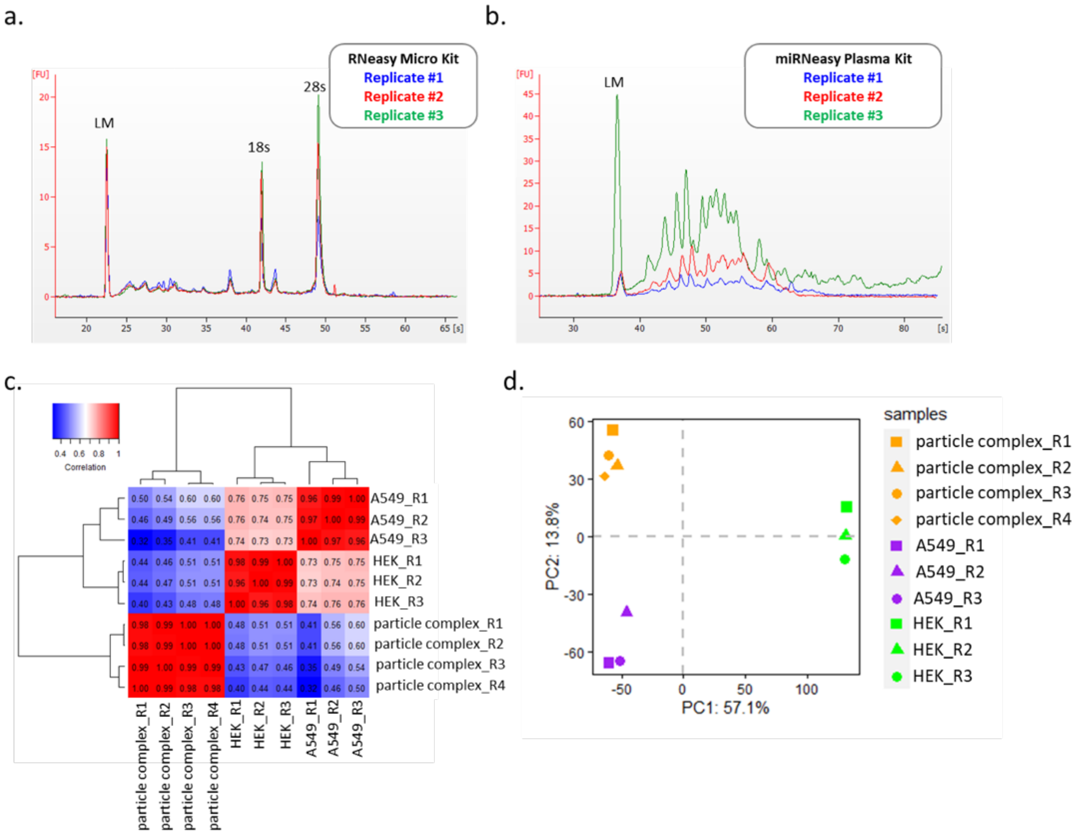

Supplementary Figure 44. Reproducibility of RNA extraction and RNA-seq. (a-b) Bioanalyzer results of three biological replicates of the RNA extracted from particle complexes derived from A549 cells by RNeasy Micro Kit (a) and miRNeasy Plasma Kit (b) operated performed by two independent operators. Correlation (c) and PCA plot (d) of the RNA-seq result for A549 cells, HEK cells, and the A549-derived particle complexes.

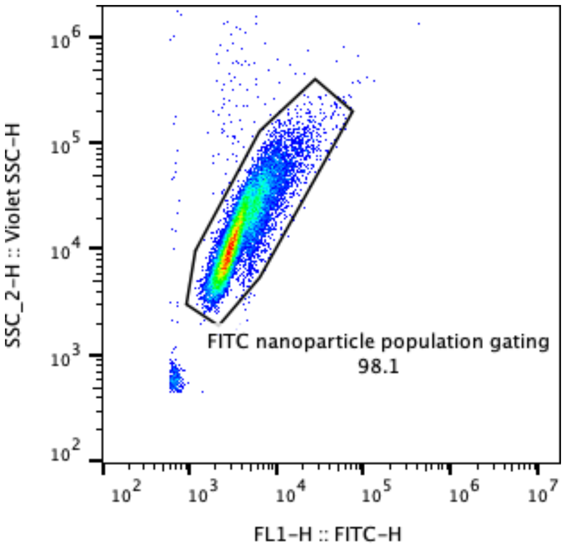

Supplementary Figure 45. An example of gating strategy used to identify fluor-labelled nanoparticle population by flow cytometry. The particle population is gated based on the Violet SSC and particle fluorescence. An example of gating for FITC-labelled nanoparticle is shown.
