# Supplementary material for: Condensate corona–nanoparticle complexes transfer functional biomolecules between cells
Source: Nat Mater. 2026 Apr 16;25(6):1045–57. doi: 10.1038/s41563-026-02534-5 (PMC13236592; doi:10.1038/s41563-026-02534-5)
Supplement: Supplementary file 2 — Reporting Summary [file 41563_2026_2534_MOESM2_ESM.pdf]

nature portfolio

Corresponding author(s): Kenneth A.Dawson & Yan Yan

Last updated by author(s): 10/01/2026

Reporting Summary

Nature Portfolio wishes to improve the reproducibility of the work that we publish. This form provides structure for consistency and transparency in reporting. For further information on Nature Portfolio policies, see our Editorial Policies and the Editorial Policy Checklist.

Statistics

For all statistical analyses, confirm that the following items are present in the figure legend, table legend, main text, or Methods section.

n/a	Confirmed	
☐	☒	The exact sample size (*n*) for each experimental group/condition, given as a discrete number and unit of measurement
☐	☒	A statement on whether measurements were taken from distinct samples or whether the same sample was measured repeatedly
☐	☒	The statistical test(s) used AND whether they are one- or two-sided<br>*Only common tests should be described solely by name; describe more complex techniques in the Methods section.*
☒	☐	A description of all covariates tested
☐	☒	A description of any assumptions or corrections, such as tests of normality and adjustment for multiple comparisons
☒	☐	A full description of the statistical parameters including central tendency (e.g. means) or other basic estimates (e.g. regression coefficient) AND variation (e.g. standard deviation) or associated estimates of uncertainty (e.g. confidence intervals)
☐	☒	For null hypothesis testing, the test statistic (e.g. *F*, *t*, *r*) with confidence intervals, effect sizes, degrees of freedom and *P* value noted<br>*Give P values as exact values whenever suitable.*
☒	☐	For Bayesian analysis, information on the choice of priors and Markov chain Monte Carlo settings
☒	☐	For hierarchical and complex designs, identification of the appropriate level for tests and full reporting of outcomes
☒	☐	Estimates of effect sizes (e.g. Cohen's *d*, Pearson's *r*), indicating how they were calculated

Our web collection on statistics for biologists contains articles on many of the points above.

Software and code

Policy information about availability of computer code

Data collection	Nikon Eclipse TI spinning disc confocal microscope built-in software and Opera Phenix® High Content Screening System (PerkinElmner) built-in software were used to acquire images.<br>Peptides are analyzed using an Orbitrap Fusion™ Tribrid™ Mass Spectrometer (Thermo Fisher Scientific), or on a timsTOF mass spectrometer (Bruker Daltonics) coupled to an Evosep One chromatography system.<br>The mRNA-seq with paired end 150 bp sequence and small RNA-seq with single end 50 bp sequence were performed on the MGISEQ-2000 platform.<br>Illumina™ sequencing was performed using paired-end 100 bp sequencing on the NovaSeq 6000 platform.
Data analysis	Imaris 1.7.6 imaging software (Bitplane) was used to analyze confocal images. Harmony® high-content analysis software was used to analyze HCA data.<br>Venn plot was generated using a web-based tool (http://www.interactivenn.net/).<br>Proteomic data were analysed using MaxQuant software (version 2.0.1.0) using built-in Andromeda search engine. The uniprot database (UniProt Homo sapiens (Human) [9606]-203800-202201.fasta) was used to retrieve data. Data normalisation and missing-value imputation were performed in R using missForest (version 1.5). Principal component analysis was conducted in R (version 4.5.1). Heat maps were generated with pheatmap (version 1.0.13). Differential analysis was carried out with R package limma (version 3.64.3). Gene Ontology enrichment was performed using Metascape and bubble plots were produced with R package ggplot2 (version 3.5.2). Circular plots of multi-located proteins were generated with PANTHER and the circlize R package.<br>The sequencing data were filtered and demultiplexed by bclfastq2 conversion software (v2.20) with default settings, and clean reads were exported in FASTQ format. The clean reads then were mapped to the human reference genome GRCH38 (GRCh38.p14.genome.fa) via HISAT2 (version 2.1.0), and gene-level counts were obtained with FeatureCounts. Principal component analysis (PCA) was performed in R (version 4.5.1). Heatmaps were generated using the R package pheatmap (v1.0.13). Differential expression analysis and volcano plots were produced

with DESeq2 (v1.48.2), and pie charts were generated using ggplot2 (v3.5.2). Gene Ontology (GO) enrichment analysis was carried out with clusterProfiler (v4.16.0). Biotype analysis of clean small RNA-seq data was performed using exceRpt and visualized in GraphPad Prism 9.

For manuscripts utilizing custom algorithms or software that are central to the research but not yet described in published literature, software must be made available to editors and reviewers. We strongly encourage code deposition in a community repository (e.g. GitHub). See the Nature Portfolio guidelines for submitting code & software for further information.

Data

Policy information about availability of data

All manuscripts must include a data availability statement. This statement should provide the following information, where applicable:

- Accession codes, unique identifiers, or web links for publicly available datasets
- A description of any restrictions on data availability
- For clinical datasets or third party data, please ensure that the statement adheres to our policy

Raw images are available from the corresponding authors upon request due to large file sizes. The mass spectrometry proteomics data have been deposited in the PRIDE repository with dataset identifier PXD071779. The RNA-seq data have been deposited in the ArrayExpress repository with accession E-MTAB-16398.

Research involving human participants, their data, or biological material

Policy information about studies with human participants or human data. See also policy information about sex, gender (identity/presentation), and sexual orientation and race, ethnicity and racism.

Reporting on sex and gender	N/A
Reporting on race, ethnicity, or other socially relevant groupings	N/A
Population characteristics	N/A
Recruitment	N/A
Ethics oversight	N/A

Note that full information on the approval of the study protocol must also be provided in the manuscript.

Field-specific reporting

Please select the one below that is the best fit for your research. If you are not sure, read the appropriate sections before making your selection.

☒ Life sciences ☐ Behavioural & social sciences ☐ Ecological, evolutionary & environmental sciences

For a reference copy of the document with all sections, see nature.com/documents/nr-reporting-summary-flat.pdf

Life sciences study design

All studies must disclose on these points even when the disclosure is negative.

Sample size	Every single figure was reproduced multiple times by the same operator and then reconfirmed by multiple operators. No statistical method was used to pre-determine the sample size, but based on the ease of reproducibility, independent sample size was chosen.<br>For proteomic samples, three or four biological replicates were chosen. For RNA-seq samples, three or four biological replicates were chosen based on the standards in the field.
Data exclusions	Data were not excluded from analysis.
Replication	Exceptional efforts were made to establish reproducibility at every stage. This included use of special facilities (such as ultra-clean synthesis and processing facilities), exhaustive sequential cleaning procedures to determining limits of contamination (such as extracellular vesicle contamination for the extraction). Replication was further audited by independent de novo experiments carried out by at least two, more often three, independent operators.
Randomization	Cell culture plates were randomly assigned to experimental conditions.
Blinding	Multiple operators were typically not informed of results from previous replicates. Proteomics and RNA-seq samples were blinded. Extensive audited files were retained and documented by administrative office.

Reporting for specific materials, systems and methods

We require information from authors about some types of materials, experimental systems and methods used in many studies. Here, indicate whether each material, system or method listed is relevant to your study. If you are not sure if a list item applies to your research, read the appropriate section before selecting a response.

Materials & experimental systems

n/a	Involved in the study
☐	☒ Antibodies
☐	☒ Eukaryotic cell lines
☒	☐ Palaeontology and archaeology
☒	☐ Animals and other organisms
☒	☐ Clinical data
☒	☐ Dual use research of concern
☒	☐ Plants

Methods

n/a	Involved in the study
☒	☐ ChIP-seq
☐	☒ Flow cytometry
☒	☐ MRI-based neuroimaging

Antibodies

Antibodies used	Cell Signalling Technologies: Rabbit anti-Glyceraldehyde-3-phosphate dehydrogenase (GAPDH) antibody (Cat. No. 14C10), mouse anti-Lamin A/C antibody (Cat. No. 4C11), Rabbit anti-NUP98 antibody (Cat. No. C39A3)<br><br>Abcam: Rabbit anti-Ki67 antigen (Ki67) antibody (Cat. No. ab92742), Rabbit anti-Lysosomal associated membrane protein 1 (LAMP1) antibody (Cat. No. ab24170), Mouse anti-Vimentin antibody (Cat. No. ab20346), Rabbit anti-Rab11 antibody (Cat. No. ab3612), Rabbit anti-beta COP antibody (Cat. No. ab2899), Rabbit anti-REEP5 antibody (Cat. No. ab167405), Mouse anti- TOMM20 antibody (Cat. No. ab56783), HRP-functionalised Goat anti-Mouse IgG (Cat. No. ab97023) and anti-Rabbit IgG (Cat. No. ab6721)<br><br>Thermofisher: Anti-alpha Tubulin antibody (236-10501), AlexaFluor® 546-functionalised Goat anti-Rabbit IgG (A11035), AlexaFluor® 546-functionalised Goat anti-Mouse IgG (A11003), Rabbit Anti–Alexa Fluor 405/Cascade Blue Dye antibody (A5760), anti-Calnexin antibody (GT1563).
Validation	Rabbit anti-Glyceraldehyde-3-phosphate dehydrogenase (GAPDH) antibody (Cat. No. 14C10), mouse anti-Lamin A/C antibody (Cat. No. 4C11), Rabbit anti-NUP98 antibody (Cat. No. C39A3) from Cell Signalling Technologies have been validated in Western blot and Immunofluorescence imaging by Cell Signalling Technololgies showing on the websites.<br><br>Rabbit anti-Ki67 antigen (Ki67) antibody (Cat. No. ab92742), Rabbit anti-Lysosomal associated membrane protein 1 (LAMP1) antibody (Cat. No. ab24170), Mouse anti-Vimentin antibody (Cat. No. ab20346), Rabbit anti-Rab11 antibody (Cat. No. ab3612), Rabbit anti-beta COP antibody (Cat. No. ab2899), Rabbit anti-REEP5 antibody (Cat. No. ab167405), Mouse anti- TOMM20 antibody (Cat. No. ab56783) from Abcam listed as above were used in immunofluorescene imaging in this study. Abcam has validated their use in immunofluorescence imaging as shown on the Abcam websites.<br><br>Anti-alpha Tubulin antibody (236-10501) and anti-Calnexin antibody (GT1563) from Thermofisher were used in immunofluorescene imaging in this study. Thermofisher has validated their use in immunofluorescene imaging as shown on the website.

Eukaryotic cell lines

Policy information about cell lines and Sex and Gender in Research

Cell line source(s)	A549 and HEK293 cell lines used in this study were purchased from ATCC. Several independent cell batches from ATCC were used during the course of this study.
Authentication	None of the cells were authenticated.
Mycoplasma contamination	Cell cultures were routinely tested for mycoplasma contamination, and no mycoplasma contamination was detected.
Commonly misidentified lines (See ICLAC register)	Cell lines used in this study were not listed in the commonly misidentified lines.

Plants

Seed stocks

Report on the source of all seed stocks or other plant material used. If applicable, state the seed stock centre and catalogue number. If plant specimens were collected from the field, describe the collection location, date and sampling procedures.

Novel plant genotypes

Describe the methods by which all novel plant genotypes were produced. This includes those generated by transgenic approaches, gene editing, chemical/radiation-based mutagenesis and hybridization. For transgenic lines, describe the transformation method, the number of independent lines analyzed and the generation upon which experiments were performed. For gene-edited lines, describe the editor used, the endogenous sequence targeted for editing, the targeting guide RNA sequence (if applicable) and how the editor was applied.

Authentication

Describe any authentication procedures for each seed stock used or novel genotype generated. Describe any experiments used to assess the effect of a mutation and, where applicable, how potential secondary effects (e.g. second site T-DNA insertions, mosiacism, off-target gene editing) were examined.

Flow Cytometry

Plots

Confirm that:

☒ The axis labels state the marker and fluorochrome used (e.g. CD4-FITC).

☒ The axis scales are clearly visible. Include numbers along axes only for bottom left plot of group (a 'group' is an analysis of identical markers).

☒ All plots are contour plots with outliers or pseudocolor plots.

☒ A numerical value for number of cells or percentage (with statistics) is provided.

Methodology

Sample preparation

Nanoparticles or particle complexes are preparation as decribed in the Methods.

Instrument

Beckman Coulter CytoFLEX LX flow cytometer equipped with 6 lasers (UV-Violet-Blue-Yellow-Green-Red)

Software

FlowJo

Cell population abundance

The events were collected more than 50,000.

Gating strategy

The gating strategy of particle population is based on violet-SSC and nanoparticle fluorescence.

☒ Tick this box to confirm that a figure exemplifying the gating strategy is provided in the Supplementary Information.
